# The molecular basis of color vision in colorful fish: Four Long Wave-Sensitive (LWS) opsins in guppies (*Poecilia reticulata*) are defined by amino acid substitutions at key functional sites

**DOI:** 10.1186/1471-2148-8-210

**Published:** 2008-07-18

**Authors:** Matthew N Ward, Allison M Churcher, Kevin J Dick, Chris RJ Laver, Greg L Owens, Megan D Polack, Pam R Ward, Felix Breden, John S Taylor

**Affiliations:** 1 University of Victoria, Department of Biology, Victoria, British Columbia, Canada; 2 Simon Fraser University, Department of Biological Sciences, Burnaby, British Columbia, Canada

## Abstract

**Background:**

Comparisons of functionally important changes at the molecular level in model systems have identified key adaptations driving isolation and speciation. In cichlids, for example, long wavelength-sensitive (LWS) opsins appear to play a role in mate choice and male color variation within and among species. To test the hypothesis that the evolution of elaborate coloration in male guppies (*Poecilia reticulata*) is also associated with opsin gene diversity, we sequenced long wavelength-sensitive (LWS) opsin genes in six species of the family Poeciliidae.

**Results:**

Sequences of four LWS opsin genes were amplified from the guppy genome and from mRNA isolated from adult guppy eyes. Variation in expression was quantified using qPCR. Three of the four genes encode opsins predicted to be most sensitive to different wavelengths of light because they vary at key amino acid positions. This family of LWS opsin genes was produced by a diversity of duplication events. One, an intronless gene, was produced prior to the divergence of families Fundulidae and Poeciliidae. Between-gene PCR and DNA sequencing show that two of the guppy LWS opsins are linked in an inverted orientation. This inverted tandem duplication event occurred near the base of the poeciliid tree in the common ancestor of *Poecilia *and *Xiphophorus*. The fourth sequence has been uncovered only in the genus *Poecilia*. In the guppies surveyed here, this sequence is a hybrid, with the 5' end most similar to one of the tandem duplicates and the 3' end identical to the other.

**Conclusion:**

Enhanced wavelength discrimination, a possible consequence of opsin gene duplication and divergence, might have been an evolutionary prerequisite for color-based sexual selection and have led to the extraordinary coloration now observed in male guppies and in many other poeciliids.

## Background

Understanding the molecular basis of characters shaped by selection is a major goal of evolutionary genetics. Of particular interest are genes that encode conspicuous secondary sexual traits in males and the genes that influence female preference for such traits [1]. Among fish; sticklebacks (genus *Gasterosteus*), cichlids, and poeciliids, including the guppy (*Poecilia reticulata*) and swordtails (genus *Xiphophorus*), are the most important models for the study of sexual selection driven by female choice. In each of these taxa, female mate choice is influenced by male coloration and in each group, male coloration and female preference have a genetic basis [2-6].

Mapping studies designed to uncover genes responsible for species- and population-level color variation in cichlids and sticklebacks are underway [7] but to date none have been identified. While it is also the case that no female preference loci have been uncovered in fish, many cichlid species and some populations possess unique opsin genes that provide strong candidates. Indeed, the only DNA sequences that have been found to differ among the 200 to 500 endemic Lake Victoria haplochromine species are long wave-sensitive (LWS) opsins [8,9]. In the cichlid genus *Pundamilia*, LWS opsin sequence and expression appears to be tuned to specific male color morphs [10]. Thus, it appears that variation in LWS opsin genes influences female mate choice and speciation in this family [11].

Opsin genes encode membrane-bound receptors that are expressed primarily in rod and cone cells of the retina. Each opsin protein is associated with a chromophore and when exposed to light, this complex changes shape leading to rod or cone cell hyperpolarization [12]. The detection of light at the receptor level requires input from just one type of opsin-chromophore receptor. However, discriminating among colors (wavelengths) involves the interpretation of signals from multiple adjacent retinal cone cells expressing different opsins. These different opsins often have names that reflect the wavelength of light to which they are most sensitive. For instance, short wave-sensitive (SWS), middle wave-sensitive (MWS), and long wave-sensitive opsins (LWS) are most sensitive to blue, green and red light, respectively. Gene duplication and divergence has generated this opsin diversity. For example, the human MWS opsin is a duplicate (or paralog) of the LWS opsin locus and now differs at three of the five amino acid positions known to influence wavelength sensitivity [13-16]. Zebrafish also have a pair of LWS opsin genes with different five key-site haplotypes [17].

The purpose of this study was to characterize LWS opsin gene sequence variation in guppies and in closely related species. We focused on this gene because microspectrophotometry (MSP) data indicated that guppies express more than one type of LWS opsin [18,19] and because orange is an important component of female mate choice for these fish [4,20-22]. While two recent studies have reported LWS opsin gene variation in guppies [23,24], one focused only on short amplicons from a single fish and both presented incomplete data on the key-site amino acids known to influence spectral sensitivity. Genomic sequences, transcript expression levels, and data from other poeciliids have also not been reported to date.

We show that guppies (*Poecilia reticulata*) and three species in the guppy sister group (Micropoecilia) have four LWS genes. Sequence variation at the five key sites indicates that three of these LWS opsins are most sensitive to different wavelengths of light providing *Poecilia *with a larger repertoire of LWS pigments than any other fish taxon. One of the guppy LWS opsins appears to be a single-exon gene, likely arising from a retrotransposition event. This gene was sequenced in all poeciliids surveyed except *Tomeurus gracilis *and has also been reported in the killifish, *Lucania goodei *(family Fundulidae). Two LWS opsins are linked, oriented in a tail-to-tail fashion, and separated by approximately 3.3 Kbp. The fourth is found only in the genus *Poecilia*. This is a hybrid or mosaic sequence in the guppies surveyed here from Cumaná Venezuela. All four LWS opsins in the guppy were amplified from RNA isolated from adult eyes, but qPCR experiments show much variation among these duplicates in the level of expression.

## Methods

### Genomic PCR and sequencing

All species surveyed are in the family Poeciliidae. Long wave-sensitive (LWS) opsin genes were amplified from DNA isolated from *Tomeurus gracilis *(one individual), *Xiphophorus pygmaeus *(one individual), and from four species in the genus *Poecilia*: *P. reticulata *(14 individuals), *P. picta *(four individuals) *P. parae *(three individuals) and *P. bifurca *(three individuals). The genus *Tomeurus *is the sister group to a clade that includes *Poecilia *and *Xiphophorus*, and most other poeciliids [25,26]. *Poecilia picta*, *P. parae*, and *P. bifurca*, occur in the sister taxon to the guppy [27]. They were in a separate genus previous to Rosen and Bailey's [28] revision of the poeciliids and we refer to them collectively as Micropoecilia. *Poecilia reticulata *(the guppy) was sampled from a population collected in Cumaná, Venezuela and bred in our laboratory aquarium. The Cumaná guppy has also been referred to as Endler's guppy, but is closely related to other guppy populations [29]. PCR reactions were run using genomic DNA isolated from fish euthanized with buffered MS222 (Sigma^® ^A5040) or from specimens preserved in 95% ethanol.

Initially, PCR and sequence data were obtained using primers ForBeg, Fw1a and Rev5, which are complementary to conserved regions of fish LWS opsin genes in exon I (ForBeg), exon II (Fw1a), and exon V (Rev5) (see Additional files 1 and 2). After uncovering multiple LWS opsin sequences in *Poecilia*, we attempted to PCR-amplify DNA between guppy opsin genes. The between-gene PCR experiment was initiated because LWS opsins occur in tandem in human, zebrafish, and medaka. It employed the reverse complement of a forward primer close to the 5' end of the gene (Fw1a Comp) and the reverse complement of a reverse primer close to the 3' end of the gene (Rev8 Comp). Sequence data from amplicons derived from primers ForBeg, Fw1a and Rev5, and the success of between-gene PCR allowed us to develop gene-specific primers, including reverse primers complementary to 3' UTR sequences. Primers complementary only to guppy LWS 'variant 6' were designed from sequence data recently published by Weadick and Chang [24]. PCR amplicons were cut and purified from agarose gels using a QIAquick^® ^Gel Extraction Kit and were cloned using the pGEM^® ^– T Easy Vector System II kit (Promega™). Sequencing of insert-positive clones utilized labeled M13 forward and reverse primers and a LI-COR sequencer at the Centre for Biomedical Research at the University of Victoria. A list of PCR primer sequences, PCR reaction conditions and a primer map can be found in Additional file 1, Additional file 2, and Additional file 3, respectively.

### Southern Blot Hybridization

DNA from one lab-reared Cumaná guppy was extracted using a Qiagen^® ^DNeasy Tissue Kit and digested with the four-cutter restriction enzyme *Bfa*I (New England Biolabs^®^). 423 bp DIG labeled probes were prepared from guppy genomic DNA using the PCR DIG Probe Synthesis Kit (Roche^®^) and primers Fw100 and Rev4 (see Additional file 1). The amplicons from this PCR reactions were purified using the QIAquick^® ^Gel Extraction Kit (Qiagen^®^). *Bfa*I does not cut any of the LWS opsins in the region complementary to the probes. Southern blot hybridization was carried out using a modified protocol from the Roche^® ^DIG application manual for filter hybridization. Digested DNA was blotted onto a Bio-Rad™ Zeta-Probe^® ^Blotting membrane using the Bio-Rad™ Model 785 Vacuum Blotter. This was followed by UV exposure (120 mJ) and probe hybridization at 40°C overnight. The blot was washed in 2× SSC at room temperature and then in 1× SSC at 65°C and visualized using the DIG Luminescent Detection Kit for Nucleic Acids (Roche^®^).

### LWS opsin gene expression using RT-PCR

Prior to quantitative PCR (qPCR) experiments (see below) we tested the hypothesis that all four LWS opsin loci were expressed using reverse transcriptase (RT)-PCR. Three guppies were euthanized in buffered MS222. A single eye from each individual was placed in 1.0 mL PureZOL™ (Bio-Rad^®^) with 3 mm tungsten carbide beads and homogenized for five minutes in a Retsch MM301 Mixer Mill. Total RNA was extracted using the Aurum™ Total RNA Fatty and Fibrous Tissue kit from BioRad^®^. The iScript™ kit (Bio-Rad^®^) was used to generate single-stranded cDNA. LWS opsin transcripts were PCR amplified and cloned using the pGEM^® ^– T Easy Vector System II kit (Promega™) and then sequenced with labeled M13 primers. Primers reported by Meyer and Lydeard [25] that amplify *XSrc *were used as a positive control. Primer sequences and PCR conditions can be found in Additional files 1 and 2.

### Quantitative PCR

Data from cichlids and zebrafish indicate that cone opsin expression is highest at the end of the day [30,31]. Guppies were maintained in a 14:10 hr light and dark cycle and qPCR experiments were performed on cDNA samples obtained from three fish in the last hour of the subjective day. Total RNA was extracted from the eyes of these fish (one adult male and two adult females) using the Retsch MM301 Mixer Mill and the Aurum™ Total RNA Fatty and Fibrous Tissue kit from BioRad^®^. Synthesis of cDNA for qPCR experiments utilized the SuperScript™ III First-Strand Synthesis SuperMix kit for qRT-PCR (Invitrogen™) and 1 μg of total RNA from the three samples. To determine the concentration of each transcript in the three cDNA samples, we used the Invitrogen™ SYBR^® ^GreenER™ qPCR SuperMix Universal kit to prepare triplet qPCR reactions. qPCR was carried out in a Stratagene^® ^Mx4000^® ^Multiplex Quantitative PCR machine with the following locus-specific primer pairs: a/sExon2 and LWS1IntRev; A180SpecFwd and rev8; pExon2 and LWS2IntRev; and fw100 plus revA (see Additional file 1). A 1:10 ROX Reference Dye normalized the fluorescent reporter signal. qPCR conditions consisted of 1 cycle at 95°C (9 minutes); 50 cycles of 95°C (15 seconds), 60°C (30 seconds), 72°C (45 seconds); 1 cycle of 95°C (1 minute); and a 40-step melting curve analysis (initial temperature 55°C, increasing 1°C every 30 seconds). Each gene was also PCR amplified, cloned using the pGEM^® ^– T Easy Vector System II kit (Promega™), sequenced to confirm identity, and then utilized for qPCR at concentrations of 1 ng, 1 × 10^-3^ng and 1 × 10^-5^ng per 16 μL reaction. Ct values from these plasmid templates were then used to generate a standard curve and to estimate qPCR efficiency (qPCR_eff _= [10^(-1/slope) ^– 1] × 100; Table 1). The plasmid template reactions were also run in triplicate. Dissociation curves (Fluorescence [-R'(T)] over T°C) and gel electrophoresis confirmed the presence of single amplicons in all qPCR reactions.

**Table 1:** Key-site haplotypes for LWS opsin duplicates in fish and humans.

Key Site Position	Guppy LWS S180	Guppy LWS A180	Guppy LWS P180	Guppy LWS S180r	Killifish LWS A	Killifish LWS B	Rice fish LWS A	Rice fish LWS B	Zebrafish LWS 1	Zebrafish LWS 2	Cave fish LWS g101	Cave fish LWS g103	Human LWS	Human MWS
180	**S**	**A**	**P**	**S**	**S**	**S**	**S**	**S**	**A**	**A**	**A**	**A**	**S**	**A**
197	**H**	**H**	**H**	**H**	**H**	**H**	**H**	**H**	**H**	**H**	**H**	**H**	**H**	**H**
277	**Y**	**Y**	**F**	**Y**	**Y**	**Y**	**Y**	**Y**	**Y**	**F**	**F**	**F**	**Y**	**F**
285	**T**	**T**	**A**	**T**	**T**	**T**	**T**	**T**	**T**	**T**	**A**	**A**	**T**	**A**
308	**A**	**A**	**A**	**A**	**A**	**A**	**A**	**A**	**A**	**A**	**A**	**A**	**A**	**A**

Expected λ_max _(nm)	~560	~553	~531 +P	~560	~560	~560	~560	~560	~553	~546	~531	~531	~560	~531

The λ_max _of each LWS opsin is estimated from the spectral shift predicted by each key-site substitution. The influence of the proline residue at position 180 is not known.

### Phylogenetic analyses

The *Tetraodon nigroviridis *LWS opsin amino acid sequence AAT38457.1 was employed as a query sequence in a BLASTp search [32] to identify homologs in the NCBI nr database. All hits with bitscores > 300 were aligned with one another and with the new data using the MPI version of ClustalW [33,34]. Subsequent sequence manipulations including multiple sequence alignments, toggle translations, hand editing, and delimitation of intron/exon boundaries utilized BioEdit v.7.0.5.3. [35]. A short nucleotide multiple sequence alignment (390 bp) that included coding sequences from exons IV and V was used to determine relationships among our new guppy LWS opsin genes and those from guppies of the Oropuche and Quare Rivers in Trinidad reported by Hoffmann et al. [23]: OR6-4 D09/DQ168660.1 and OR6-3 EO8/DQ168659.1 and QUEm5 LO6/DQ168661.1, and from the Paria River (Trinidad) guppy reported by Weadick and Chang [24]: DQ865167.1, DQ865168.1, DQ865169.1, DQ865170.1, DQ865171.1, and DQ865172.1. Maximum parsimony (MP) and Neighbor-joining (NJ) trees [36], which were based upon Tamura-Nei [37] distance estimates were reconstructed using MEGA v.4 [38]. Both analyses utilized all codon positions. Support for nodes was assessed using 1,000 bootstrap reiterations.

The MP and NJ analyses were repeated using an alignment of nucleotide sequences that varied in length from 619 to 1095 bp. LWS opsin sequences in this analysis included the following species and acquisition numbers: Zebrafish (*Danio rerio*), AB087803.1 and AB087804.1; Medaka (*Oryzias latipes*) AB223051.1 and AB223052.1; Bluefin killifish (*Lucania goodei*) AY296740.1 and AY296741.1; Blind cave fish (*Astyanax mexicanus*) M90075.1, U12024.1, and U12025.1; Sea chub (*Girella punctata*) AB158261.1; Nile tilapia (*Oreochromis niloticus*) AF247128.1; Fugu (*Takifugu rubripes*) AY598942.1; Spotted green pufferfish (*Tetraodon nigroviridis*) AY598943.1; Turbot (*Scophthalmus maximus*) AF385826.1; Winter flounder (*Pseudopleuronectes americanus*) AY631039.1; Goldfish (*Carassius auratus*) L11867.1; Coho salmon (*Oncorhynchus kisutch*) AY214145.1; Ayu smelt (*Plecoglossus altivelis*), AB098702.1 and AB107771.1; Atlantic halibut (*Hippoglossus hippoglossus*) AF316498.1; Carp (*Cyprinus carpio*) AB055656.1; human (*Homo sapiens*) NM_020061.3 and NM_000513.1; Arctic lamprey (*Lethenteron japonicum*) AB116381.1; and our new sequences from the Cumaná guppy (*Poecilia reticulata*), Picta or 'swamp guppy' (*Poecilia picta*), Parae (*Poecilia parae*), Bifurca (*Poecilia bifurca*), the Pygmy swordtail (*Xiphophorus pygmaeus*) and Tomeurus (*Tomeurus gracilis*).

## Results

### Hybrid or mosaic sequences

A large number of LWS opsin-like sequences (up to 17 per guppy) were uncovered after cloning and sequencing the products of PCR reactions utilizing primers Fw1a and Rev5. These sequences included suspected recombinants, that is, sequences that could have been generated by the ordered concatenation of fragments of other sequences produced in the same PCR reaction. Template switching during PCR and/or mismatch repair of cloned heteroduplex molecules has been shown to generate such artefacts [39-42]. To test the hypothesis that PCR and cloning could generate LWS opsin sequences not found in the guppy genome, we used primers Fw1a and Rev5 to re-amplify DNA from a two-sequence template (i.e., two insert-bearing plasmids). Five different sequences were uncovered from the two-template PCR reaction; one copy of each of the two templates and three recombinant sequences. These two-template experiments confirmed speculation by Hoffmann et al. [23] and Weadick and Chang [24] that LWS opsin genes in poeciliids are susceptible to PCR and/or cloning artefacts that generate artificial hybrid sequences.

To determine the minimum number of genuine LWS opsin sequences in our dataset we first considered variation at polymorphic positions. Two sequences (e.g., from two different loci or from two alleles at one locus) could serve as a PCR template for the generation of an enormous diversity of hybrid sequences via template switching or mismatch repair. However, among such a set of hybrid sequences there would be only two variants (substitutions or indels) at a given polymorphic site. LWS opsin sequences derived from individual fish using primers Fw1a and Rev5 included three different intron II haplotypes and a position in exon III that was polymorphic for three different nucleotides. Remarkably, this exon III variation translated into amino acid variation at position 180; the first of the five sites known to influence spectral sensitivity (see below). Gene duplication is the only explanation for the occurrence of three haplotypes in a single individual. We set out to strengthen this evidence for LWS opsin gene duplication by amplifying DNA between the genes (see next section).

### Between-gene PCR and sequencing

PCR using primers designed to amplify between-gene DNA (Fw1a Comp and Rev8 Comp) produced a ~4 Kbp product in the guppy and in the three species of the guppy sister group Micropoecilia (*P. picta*, *P. parae*, and *P. bifurca*). These amplicons were cloned in three of these four species (cloning of this amplicon was unsuccessful in *P. bifurca*) and approximately 1500 bp were sequenced from each end of the clone insert. Each end of the insert contained the last intron and exon of an LWS opsin gene and approximately 790 bp beyond the stop codon. The explanation for this sequence pattern, confirmed by subsequent PCR experiments using only Rev8 Comp, was that this fragment was amplified with Rev8 Comp acting as a forward and a reverse primer and that it contained the ends of two LWS loci oriented in an inverted (tail-to-tail) fashion. The between-gene fragment did not amplify from *X. pygmaeus *or *T. gracilis*. In the guppy, additional primers were designed and the entire intergenic sequence was characterized. It was 3329 bp long, 66% A/T, and contained a short compound microsatellite; (TGGA)_10_(TA)_9_.

### LWS opsins in the family Poeciliidae

Given the evidence for the artificial generation of opsin sequence variation during PCR or cloning, and the observation that artefacts produced by template switching and/or mismatch repair do not appear to be reproducible [41,43], only haplotypes recovered from multiple independent PCR and cloning experiments were assumed to represent genuine opsin sequences. Additional primers were designed from these reliable sequences and from the sequences obtained by the between-gene PCR experiments described above (see Additional file 3).

Initially, three different LWS opsin sequences were identified in guppy (*P. reticulata*). These three genes were delimited by variation at codon 180: TCT (serine), GCT (alanine), and CCT (proline), and by unique intron II and intron V mutations. Codon 180 is one of the five key positions that influence wavelength sensitivity [16] and the variation uncovered here is reflected in the names we have given to each of the loci; *LWS S180 *(S for serine), *LWS A180 *(A for alanine), and *LWS P180 *(P for proline).

**Figure 1:**
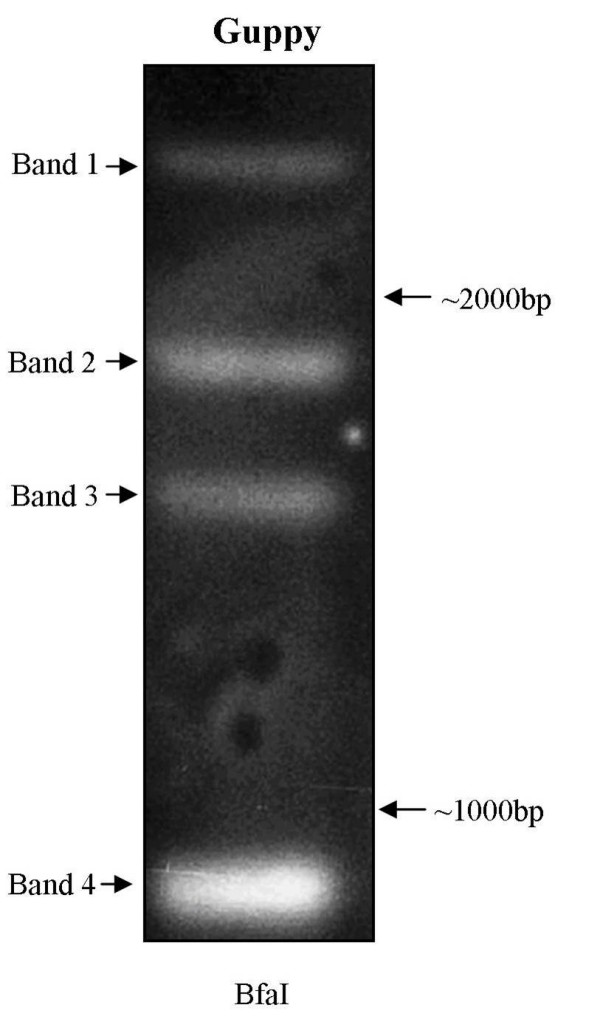
**Southern blot hybridization for determination of LWS opsin copy-number in Poecilia reticulata**. Four bands (labeled 1–4) correlate with four LWS loci from a single Cumaná guppy. *BfaI *(New England Biolabs^®^) was the restriction enzyme used. A generic DIG-labeled probe was designed to target all LWS loci (see methods). Two size markers (given in base-pairs) are shown to the right of the blot.

Seven *LWS S180 *sequences were obtained from six different Cumaná guppies with five of these including the start codon, all exons and introns, and part of the 3' UTR (see Additional file 3). Thirteen *LWS A180 *sequences were obtained from seven guppies. Only one was full-length but six included sequence from exon II to the 3' UTR. The *LWS A180 *sequence appears to be a naturally occurring hybrid in the Cumaná guppy; the first five exons and four introns are most similar to *LWS S180*, whereas the last intron and exon are identical to the *LWS P180 *locus. As the two regions of this hybrid *LWS A180 *sequence will give conflicting phylogenetic signals, the *LWS A180 *sequences were truncated in the phylogenetic analyses reported below (i.e. only the first five exons were utilized). Seven *LWS P180 *sequences were obtained from seven guppies (see Additional file 2). The ForBeg primer, which includes the start codon, combined with any of the reverse primers, did not amplify the *LWS P180 *locus. Therefore, *LWS P180 *sequences spanned exon II to the 3' UTR. In addition to the proline residue at site 180, the *LWS P180 *locus has amino acids substitutions at two other key sites. In guppies, *LWS P180 *also possessed a variable-length tetranucleotide microsatellite in intron III. PCR experiments using Fw100 and a primer complementary only to Weadick and Chang's [24] variant 6 (RevA) uncovered a fourth LWS opsin gene. While we did not amplify or sequence the first exon or intron, we show that the rest of this gene is intronless, suggesting that variant 6 is a single-exon gene, arising from a retrotransposition. It has a serine at position 180 (codon: TCG) and is renamed *LWS S180r *(S for the serine at position 180 and r for retrotransposition). Finally, our southern blot shows four bands (Fig. 1), consistent with the PCR-based hypothesis that the Cumaná guppy has four LWS loci. These four LWS opsins encode the following five key-site haplotypes: SHYTA (*LWS S180 *and *LWS S180r*), AHYTA (*LWS A180*), and PHFAA (*LWS P180*) and are thus expected to be most sensitive to three different wavelengths of light [16] (Table 1).

Long portions of the four LWS genes found in the guppy were also amplified and sequenced from *P. picta *and *P. bifurca*. Three of these opsins, *LWS S180*, *LWS S180r*, and *LWS P180*, were sequenced from *P. parae*. We did not obtain the 3' end of *LWS A180 *from any of these three species. Therefore the mutation producing the hybrid sequence consistently recovered from Cumaná guppy cannot yet be mapped onto the poeciliid phylogeny. *Xiphophorus pygmaeus *had three LWS opsin genes: *LWS S180*, *LWS S180r*, and *LWS P180*. Only one LWS opsin sequence (*LWS S180*) was recovered from *Tomeurus gracilis*. All sequences have been deposited in GenBank under accession numbers EU329428 – EU329486 (see Additional file 2).

### Phylogenetic analysis of LWS opsin gene duplication in Poeciliidae

The guppy LWS opsin sequences obtained here and those reported by Hoffman et al. [23] were added to the 390 bp alignment reported by Weadick and Chang [24]. Phylogenetic analyses sorted these guppy LWS opsins into three well-supported clades; *LWS S180r*, *LWS P180 *and *LWS S180 *plus *LWS A180*. Weadick and Chang's [24] variant 6 clustered with the single exon gene *LWS S180r*, and variant 5 clustered with the *LWS P180 *gene. This last result was anticipated before phylogenetic reconstruction because both sequences encode a phenylalanine at position 277 and an alanine at position 285. The remaining guppy LWS opsin genes; variants 1–4 from Weadick and Chang [24], the three LWS opsins from Hoffman et al. [23] (LWS_OR6-4_D09, LWS_OR6-3_E08, LWS_QUEm5_L06) and our *LWS S180 *and *LWS A180 *genes, formed the third clade. Over this 390 bp alignment, these genes are almost identical (mean percent identity = 98.6%). We suspect that the Weadick and Chang [24] variants 1–4 and the three Hoffman et al. [23] LWS genes include alleles at the *LWS A180 *and *LWS S180 *loci. This guppy-only LWS opsin sequence comparison also revealed that Weadick and Chang's [24] variant 5 is a recombinant or mosaic sequence; the first 221 bp are identical to variant 4, and the last 170 bp are identical to our *LWS P180 *sequence. In our phylogenic analysis, this gene occurred in the *LWS P180 *clade because the region where it is identical to variant 4 has few phylogenetically informative characters.

**Figure 2:**
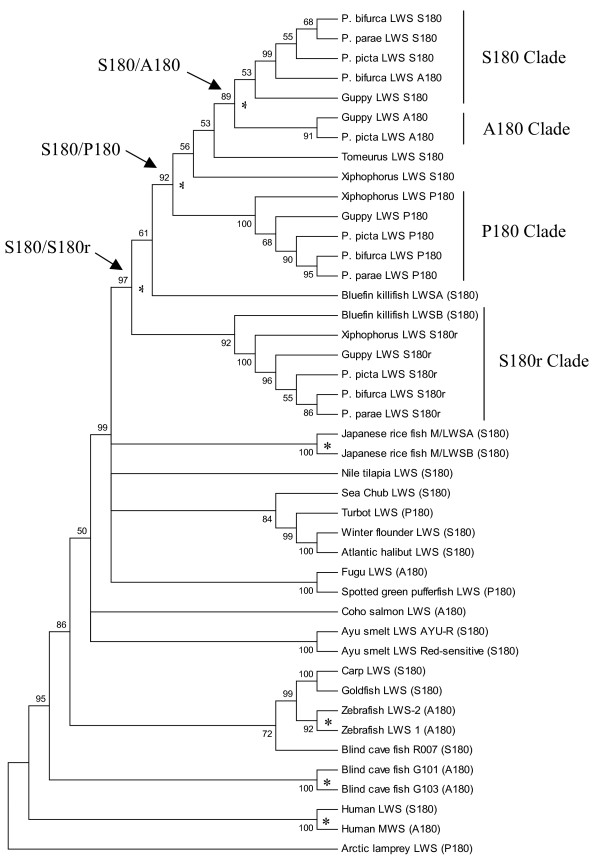
**Phylogenetic analysis of LWS opsin genes**. A Maximum Parsimony (MP) bootstrap consensus tree of long wavelength-sensitive (LWS) opsins from representative ray-finned fish lineages. The percentage of trees in which the associated taxa clustered in the bootstrap re-analyses (1000 replicates) is shown. Nodes in less than 50% of the bootstrap trees were collapsed. All codon positions were included and gaps treated as missing data. There were 1101 positions in the alignment and 520 were parsimony informative. The tree was rooted with the arctic lamprey LWS gene. Duplication events marked with an asterix. Phylogenetic analyses utilized MEGA4 [77] and the alignment from Additional file 4.

Maximum parsimony analysis of the longer multiple sequence alignment (see Additional file 4) with the new poeciliid sequences and LWS opsins from a diversity of ray-finned fish produced a single tree (Fig. 2). The NJ tree included all of the nodes from the MP tree that had bootstrap support >65% and many of the nodes with lower support. Unlike the MP tree in Fig. 2, the NJ analysis placed the *Tomeurus *LWS opsin as the sister sequence to a clade with the *Xiphophorus *and *Poecilia LWS P180*, *LWS S180 *and *LWS A180 *genes. This reconstruction makes more sense than the MP tree with respect to poeciliid taxonomy; morphological and molecular data indicate that *Poecilia *and *Xiphophorus *are more closely related to one another than either is to *Tomeurus*. However, we present the MP tree because the neighbor-joining tree also placed the bluefin killifish LWSA gene at the base of the *LWS S180r *clade and the *Xiphophorus LWS S180 *gene at the base of the *LWS P180 *clade. The number of gene duplication events and gene losses required to reconcile such a topology with the well-supported taxonomic relationships among these species makes these components of the NJ topology very unlikely.

The MP tree indicates that the guppy LWS opsin repertoire is a consequence of gene duplication events that occurred i) prior to the divergence of families Fundulidae and Poeciliidae, ii) in the common ancestor of *Xiphophorus *and *Poecilia*, and iii) within the genus *Poecilia*. Mechanisms of LWS opsin gene duplication include retrotransposition (producing *LWS S180r*) and inverted tandem duplication (producing the gene pair, *LWS S180 *and *LWS P180*). Formation of the hybrid *LWS A180 *locus may have involved quasipalindrome correction [44]. These three duplication events have provided species in this genus with a larger repertoire of LWS opsin pigments than any other fish taxon.

### LWS gene evolution in teleosts

Relationships among higher taxonomic groups were well resolved in the tree reconstructed from LWS opsin sequences. There is high (>75%) bootstrap support for monophyly of Cyprinidontiformes (the bluefin killifish and all poeciliids), Pleuronectiformes (sea chub, turbot and flounder), Percomorpha, and the family Cyprinidae (goldfish, carp and zebrafish). One of the blind cavefish (*Astyanax mexicanus*) LWS genes (R007) is the sister sequence to those from goldfish, carp and zebrafish, which is not surprising as all species are in the taxon Ostariophysi. However, there were also two cavefish LWS genes at the base of the actinopterygian clade (G101 and G103). These genes might be derived from a gene produced during the fish-specific whole genome duplication event [45]. Long Branch Attraction (LBA) occurs when rapidly evolving sequences are attracted to the base of a tree [46] and is an alternative explanation for the position of the cavefish duplicates in our analysis. However, LBA is an artefact that is usually correlated with poor taxonomic sampling, which is not the case here.

Among LWS sequences available for all ray-finned fishes, there is much variation at the five sites that influence spectral sensitivity most (see Additional file 4). Three different amino acids were observed at position 180 (A, S, or P), two at position 277 (Y or F), and two at position 285 (T or A). SHYTA is believed to be the ancestral five-site haplotype for vertebrates [16]. Serine to alanine substitutions at position 180 are common, but only guppies, turbot, and the spotted green pufferfish have a proline in this position. Lamprey, though not a ray-finned fish, also has a proline at position 180. Amino acid variation at positions 197 and 308 are also known to influence spectral sensitivity, however, all ray-finned fish surveyed to date possess only H197 and A308. LWS opsins have been duplicated at least six times in ray-finned fishes; twice within Poeciliidae, once prior to the divergence of Fundulidae and Poeciliidae, once in medaka (*Oryzias latipes*), once in zebrafish (*Danio rerio*), and again in the blind cave fish (*Astyanax mexicanus*). Only in poeciliids and zebrafish (and humans), has the duplication been followed by a substitution at one or more of the key sites (Table 1).

### LWS opsin gene expression

**Figure 3:**
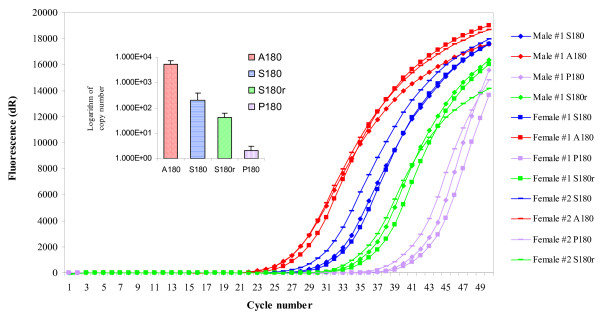
**Quantitative PCR amplification plot and logarithm histogram of original copy number**. Four LWS transcripts from the cDNA of three adult guppies were quantified using qPCR with the amplification plot shown. Ct (dR) values are given in Table 2. A histogram showing the averaged logarithm value of original transcript copy number is shown (with standard error bars included).

RT-PCR experiments (see Additional file 2) show that all four transcripts were expressed at the same time in the eyes of adult guppies. We then used qPCR to compare transcript copy numbers. In three adult guppies, *LWS A180 *was expressed at a much higher level than *LWS S180 *and *LWS S180r *(approximately 26 and 127 times greater, respectively). *LWS P180 *was expressed at very low levels, with approximately 5338 times less transcript abundance than *LWS A180 *(Fig. 3). The amount of cDNA in the three samples was estimated by comparing critical threshold (Ct (dR)) values between samples and standard curves prepared from plasmids containing each transcript (Tables 2 and 3). Standard curve log fit values are shown in Table 3.

**Table 2:** Quantitative PCR data.

Individual	Transcript	Ct (dR)	Original Copy Number
Male	*A180*	24.35	6758
Male	*S180*	29.54	121
Male	*S180r*	33.12	39
Male	*P180*	39.01	1+
Female #1	*A180*	25.46	3126
Female #1	*S180*	30.10	78
Female #1	*S180r*	33.90	24
Female #1	*P180*	39.58	1+
Female #2	*A180*	24.49	6131
Female #2	*S180*	27.93	416
Female #2	*S180r*	32.36	63
Female #2	*P180*	37.51	1+

Ct (dR) values are shown for each LWS opsin transcript from three adult guppies. Original copy number was calculated using Ct (dR) values and the standard curve data shown in Table 3.

**Table 3:** Standard curve log fit values for quantitative PCR.

(Plasmid) Standard Curve	Linear Equation	Efficiency (%)	RSq
*A180*	Y = -3.315(logX) + 37.05	100.3	0.999
*S180*	Y = -2.992(logX) + 35.77	115.9	0.996
*S180r*	Y = -3.670(logX) + 38.96	87.3	0.985
*P180*	Y = -3.319(logX) + 36.68	100.1	0.999

The linear equation, efficiency and r-squared (RSq) values for the qPCR standard curve are shown. Plasmid copy numbers used as standards were determined by calculating the number of moles in each sample multiplied by Avogadro's number. A standard curve of Ct (dR) was used to calculate original copy number of mRNA transcripts in the diluted cDNA samples.

## Discussion

### Variation at key sites among LWS opsin gene duplicates in Poeciliidae

The guppy (*Poecilia reticulata*) and species in its sister group Micropoecilia possess four LWS opsin genes that we have named *LWS S180*, *LWS S180r*, *LWS A180*, and *LWS P180*. The first two genes encode proteins with the five key-site haplotype, SHYTA, and the second two genes encode proteins with key-site haplotypes, AHYTA and PHFAA, respectively. Three of these genes; *LWS S180*, *LWS S180r*, and *LWS P180*, were also amplified and sequenced from pygmy swordtail (*Xiphophorus pygmaeus*) genomic DNA. We found only the *LWS S180 *gene in *Tomerus gracilis*. Serine (S) and alanine (A) are common residues at position 180, but proline (P) is rare. This proline residue encoded by *LWS P180 *might disrupt the transmembrane domain [47,48] and compromise opsin protein function [48,49]. However, several observations suggest that it is functional. First, *LWS P180 *is at least 44 million years old, as it evolved before the divergence of *Poecilia *and *Xiphophorus *[26], and it has no other amino acid substitutions that are expected to disrupt function. Second, the *LWS P180 *locus has diverged from paralogous LWS opsins in ways that are expected to enhance color vision; positions 277 and 285 have experienced a tyrosine to phenylalanine and threonine to alanine substitution, respectively. These are the same key-site substitutions involved in the evolution of an MWS opsin from an LWS opsin in humans. Third, this gene is expressed, albeit at very low levels. Finally, LWS opsins from arctic lamprey, turbot, and the spotted green pufferfish also have a proline at position 180 and no other substitutions likely to disrupt protein function.

All four LWS opsins uncovered in this study are predicted to have unique roles in color vision. With three different five key-site haplotypes, they are predicted to be most sensitive to three different wavelengths of light. Also, despite encoding a gene with the same key-site haplotype as *LWS S180*, the *LWS S180r *opsin differs from all other LWS opsins at amino acid positions known to play a role in binding and activating transducin [24].

Southern blot experiments in our study revealed four bands (Fig. 1) consistent with the hypothesis that Cumaná guppies have four LWS opsin loci. Hoffman et al. [23] produced a southern blot with only three bands and suggested that guppies have a minimum of two LWS opsin genes. Variation in LWS opsin gene number among populations may be another trait guppies share with humans [50-52].

Two of the four LWS opsin genes described here (*LWS S180 *and *LWS A180*) were reported in Hoffman et al.'s [23] study of guppies from the Quare and Oropuche Rivers in Trinidad. Although sequence data reported by Weadick and Chang [24] did not include all five key sites, our phylogenetic analysis indicates that Weadick and Chang [24] sequenced portions of all four loci from their Paria River guppy. The phylogenetic relationships among guppy LWS opsin paralogs reported by Weadick and Chang [24] differ from those shown here. Both topologies were produced using maximum parsimony, but by surveying more individuals, more species and by obtaining longer sequences we have produced a larger set of parsimony-informative characters. This is most evident when considering the relationship between *LWS P180 *and Weadick and Chang's variant 5, and variant 4. The large number of differences between variant 5 and variant 4 are apparent in Weadick and Chang's [24] tree where maximum likelihood branch lengths have been superimposed on the MP topology. Nonetheless, in their analysis, these two sequences form a monophyletic group. However, the sister sequence relationship between variant 5 and variant 4 disappears with the addition of *LWS P180 *genes from other *Poecilia *species and *Xiphophorus pygmaeus *because many of the unique nucleotides (autopomorphies) in variant 5 become synapomorphies (shared derived traits) in this larger dataset. Also, two of the three characters that had united variant 5 and variant 4 in Weadick and Chang's [24] MP analysis (adenines at positions 126 and 213 in their alignment), appear to be homoplasious when compared to a much larger set of *LWS P180 *and *LWS S180 *sequences. The origin of these apparent homoplasies is intriguing and is discussed below.

### Mechanisms of LWS duplication in poeciliids

The first duplication event that expanded the guppy LWS opsin repertoire produced two genes that have retained SHYTA five key-site haplotype: *LWS S180 *and *LWS S180r*. The later duplicate is missing introns II-V and is likely a product of retrotransposition. Partial cDNA sequences for each gene have been reported [24] but their intron-exon structure was unknown until now. It is not clear how *LWS S180r *has retained retinal expression, although it may occur in the vicinity of other LWS opsins (and their regulatory modules) in the guppy genome. Of interest, gene duplication by retrotransposition has also produced a pair of fish rhodopsin 1 (RH1) genes called *errlo *and single-exon *rho *[53]. *errlo *and *rho *occur on different chromosomes. Therefore, the observation that *rho *is expressed in the retina (rod cells) [53] demonstrates that upstream regulatory elements can be retained during retrotransposition.

In medaka (*Oryzias latipes*) and zebrafish (*Danio rerio*), duplicated LWS opsins are linked and oriented in a head-to-tail manner [54,55]. Phylogenetic analysis shows that independent mutations produced these gene pairs [54]. Our study also characterized an LWS opsin tandem duplication event. Duplication of the *LWS S180 *gene early during the evolution of poeciliids, produced an LWS opsin that retained the SHYTA haplotype (*LWS S180*) and an LWS opsin that evolved a PHFAA haplotype (*LWS P180*). These two genes are linked, but in an inverted (i.e., tail-to-tail) orientation. Several models have been proposed to explain the formation of inverted duplicates. Secondary rearrangement after duplication by unequal sister chromatid exchange is one. Another is intra-chromosomal replication slippage in *trans *[56]. This occurs when the DNA polymerase reverses direction using either the nascent strand (intra-molecular strand switch) or opposite strand (inter-molecular strand switch) as a template. By running backwards, a duplicate of the just-completed sequence is produced in an inverted orientation before the polymerase switches back to the correct template. The DNA downstream of *LWS S180 *has strings of adenines and thymines (data not shown) that might have facilitated strand switching by the polymerase during DNA replication [57]. The inverted arrangement of LWS opsins in the *Poecilia *genome might make them even more prone to additional duplication events [58]. Therefore, variation in LWS gene number (among species or populations) would not be surprising. *Xiphophorus pygmaeus *also has the *LWS P180 *gene. Post-duplication gene transposition or the expansion of intergenic DNA are possible explanations for our failure to amplify DNA between *LWS P180 *and *LWS S180 *in this species.

The third and most recent LWS gene duplication uncovered in our study lead to the production of *LWS S180 *and *LWS A180*. In the Cumaná guppy, the first five exons and four introns of *LWS A180 *are most similar to *LWS S180*, and the last intron, exon, and 3' UTR are identical to *LWS P180*. These data suggest that in this population *LWS A180 *is a hybrid gene; its formation might have been facilitated by the inverted tandem orientation of *LWS S180 *and *LWS P180 *[44]. As mentioned above, variant 5, reported by Weadick and Chang [24] is also a hybrid sequence, with approximately one half of the sequence identical to the *LWS S180 *gene and the other half identical to *LWS P180*. As is the case for *LWS A180 *reported here in the Cumaná guppy, the position of variant 5 in the tree based upon the 390 bp alignment depends upon which fragments are used in the phylogenetic analysis (i.e., it would occur in the *LWS S180*/*A180 *clade if only the first 220 bp were utilized). As there are many more phylogenetically informative characters in the second half of this sequence, variant 5 was placed in the *LWS P180 *clade when the entire sequence was used, despite being identical to *LWS A180 *sequences over the first 220 bp. The observation that variant 5 has not diverged from either of its progenitor sequences and that it was recovered only once by Weadick and Chang [24] leads us to the conclusion that while both halves of the sequence can be found in the guppy genome, their concatenation is an artefact produced by template switching or mismatch repair during cloning.

In the larger alignment (Fig. 2), *LWS S180 *and *LWS A180 *are not partitioned into monophyletic clades. For instance, the *P. bifurca *sequence that clusters with the *LWS S180 *genes has an alanine at position 180. One explanation for this observation is that the *P. bifurca LWS A180 *sequence is an allele of the *LWS S180 *locus. A similar situation occurs in non-African humans where a common allele of the LWS opsin locus (which typically has the SHYTA haplotype) has an alanine in position 180 and thus, an AHYTA five key-site haplotype [59,60].

### The evolutionary consequences of LWS opsin duplication in guppies

The evolutionary implications of opsin gene duplication and divergence depend largely upon the expression patterns of these genes. In several species, the possession of a large opsin family allows the retina to be spectrally tuned for different environments and/or life stages. For example, eels (*Anguilla anguilla*) have two rhodopsins, each tuned to slightly different wavelengths. They express a green-shifted locus as juveniles in fresh water and a paralogous blue-shifted locus when they return to the ocean and mature [61]. The lamprey (*G. australis*) also adjusts its spectral sensitivity by changing opsin gene expression as it moves between marine and riverine environments [62]. In cichlids, opsin gene expression varies during development [63]. Of particular note is the observation that variation in LWS opsin sequence and expression is associated with variation in water turbidity [9,64]. This has lead to the hypothesis that species- and population-level differences in opsin gene sequence and expression represent adaptations for foraging in either turbid or clear water and that these differences in spectral sensitivity may drive and/or maintain divergence in male coloration via sexual selection [11]. Guppies, however, do not move very far during their lifetime [65] and thus differential use of opsin gene duplicates in different habitats is an unlikely explanation for the evolution of LWS opsin gene diversity in this taxon.

The simultaneous expression of opsin paralogs with different sensitivities might expand the region of the spectrum where the guppy possesses high sensitivity and expand the range of detectable wavelengths. This enhancement and broadening of wavelength sensitivity can occur when individual cone cells express more than one opsin gene [66] or when adjacent cone cells express different opsins (e.g., humans and transgenic mice – see below). MSP data in guppies showing cells with a broad range of sensitivities in the long wave region of the spectrum [18,19] are consistent with the hypothesis that the sensitivity of some cones is a consequence of the co-expression of different LWS opsins. By providing guppies with a broad region of maximum wavelength sensitivity, LWS opsin gene duplication and divergence might make multi-colored male guppies appear brighter (more conspicuous) [67] to other guppies, but not to predators with wavelength sensitivity limited (by LWS opsin gene copy number) to a narrower region of the spectrum.

Expression of different LWS opsins in adjacent cones not only improves overall spectral sensitivity, but is the basis of wavelength discrimination. Observations from humans and mice are consistent with the hypothesis that opsin gene duplication and divergence can lead to better color discrimination even without any associated revisions to neuroanatomy. Among human women who are heterozygous at either the LWS or MWS locus, some appear to have a pattern of X-inactivation that leads to tetrachromacy. These women see an average of 10 colors in a spectrum, whereas trichromatic women typically see seven [[68], but see reference [69]]. In mice, the hypothesis that extra opsin genes can improve wavelength discrimination was supported by data from females expressing an SWS opsin and two LWS opsins (an endogenous LWS gene on one X chromosome and a human LWS opsin on the other). These knock-in mice performed better in wavelength discrimination tests than wild-type mice with a single LWS gene [70].

Sexual selection in guppies favors males with more red, orange, and yellow color patches [71-73] suggesting that females use color diversity (chroma) to evaluate males. This may be because males with more chroma are more conspicuous [67]. However, the 'extra' chroma is a consequence of the guppy visual system and this conspicuousness may not, therefore, apply to predators. Finally, if LWS opsin gene duplication improves motion detection, as proposed by White et al. [74] then female guppies might also be 'pre-adapted' to evaluate the well-characterized sigmoid display, a behavior that consists of the male arching its body into a S-shape and oscillating the long axis of the body both horizontally and vertically [75].

### LWS expression in the guppy

Gene expression data will help us to test alternative hypotheses about the adaptive value of LWS opsin diversity. To expand the range of maximum sensitivity and enhance wavelength discrimination, it is necessary that different opsins be expressed at the same time. All four LWS opsin gene transcripts were amplified from cDNA derived from adult eyes in our lab and by Weadick and Chang [24]. However, our qPCR experiments on three adults (1 male, 2 females) showed that most of the LWS opsin mRNA in the Cumaná guppy retinas was derived from the *LWS A180 *gene. Human SWS (blue) cone cells make up only 15% of the retina cone cell repertoire, yet play an important role in wavelength discrimination. Therefore, qPCR data showing unequal expression among LWS opsin paralogs in three adults do not rule out a role for LWS opsin gene duplication and divergence in wavelength discrimination in guppies, but does indicate the need for further investigation. We are currently using qPCR to examine LWS expression in a larger sample of adults and in fish at different stages of development. Finally, duplicated opsins are sometimes expressed in different regions of the retina [55,76]. In-situ hybridization experiments are underway to test the hypothesis that different LWS opsin paralogs have unique expression domains within the guppy retina, as is the case in zebrafish [76].

## Conclusion

Gene duplication and divergence has provided *Poecilia *and its close relatives with four distinct LWS opsins; a larger repertoire than any other fish. Phylogenetic analyses suggest that three of these LWS opsins (*LWS S180r*, *LWS P180 *and *LWS S180*) were present very early in poeciliid evolution and are predicted to occur in all of the approximately 239 species of the subfamily Poeciliinae. Adult guppies express all four LWS paralogs simultaneously, albeit at varying levels. As a consequence of these gene duplications, the potential for a broad region of high sensitivity and/or enhanced wavelength discrimination in the long-wave portion of the visible spectrum, may have facilitated a red-orange color bias for sexual selection within the guppy.

## Authors' contributions

JST supervised the study. MNW and JST designed and implemented wet-lab experimentation and data-analysis. AMC discovered the *LWS P180 *locus and carried out the template switching/mismatch repair experiment. KJD carried out the between-gene PCR and sequencing experiments. CRJL and GLO assisted with RT-PCR, qPCR and sequencing of the S180r locus. FB and JST obtained all samples. MDP and PRW assisted in RT-PCR. MNW and JST wrote the manuscript with editing assistance from FB.

## Supplementary Material

Additional file 1 List of LWS primer names and sequences used for PCR, RT-PCR and qPCR. Primer names and numbers correspond to reaction conditions shown in Additional file 2. Primer numbers corresponds to amplicons shown in Additional file 3. Sequences are given in the 5'to 3' orientation. Primers were synthesized by Operon^® ^Biotechnologies, suspended in sterile buffered TE (pH 7.0) and stored for no longer than one year at -20°C. Click here for file

Additional file 2 Sequence data and PCR conditions for specific primer combinations used for all six species of Poeciliidae. Primer names are given with corresponding primer number from Additional file 1. Amplicons are shown in Additional file 3 along with corresponding n value. 0.5 U iProof™ DNA polymerase (BioRad^®^) was used for each reaction with 5× iProof™ HF Buffer, 10 mM dNTP mix, ~100 ng template DNA, 0.5 μM of both forward and reverse primers and dH_2_O. An Eppendorf^® ^silver block thermal cycler was used for all PCR reactions. Each reaction included an initial denaturation of 94°C for 30 seconds and a final extension of 72°C for 600 seconds. If >1 bands were found after gel electrophoresis, expected sized amplicons were cut and purified using a QIAquick^® ^Gel Extraction Kit. Click here for file

Additional file 3 Primer and sequence maps for LWS opsin loci in Poeciliidae. Large colored boxes and horizontal bars represent exons and introns, respectively. Black and solid horizontal lines represent genomic sequences amplified using various PCR primer combinations (see legend; see Additional files 1 and 2). Dotted horizontal lines represent mRNA transcripts acquired through RT-PCR from cDNA samples. "n" represents the number of times a given sequence of near perfect identity had been acquired. Key site amino acids are shown within their respective exons. Inter-species and individual sequence differences due to SNP's, small strings of nucleotides, or codon selection (see discussion) are omitted and a consensus map has been created. Yellow exons and introns are highly similar to the guppy LWS S180 locus. Blue coloration represents sequences of high identity to the guppy LWS P180 locus. This use of color is exploited to highlight the hybrid nature of the LWS A180 gene in the guppy. Finally, green coloration represents the diverged, single exon LWS S180r locus (originally detected by Weadick and Chang [24]). Click here for file

Additional file 4 Exon alignment of LWS genes used in phylogenetic analysis. Common names are listed and acquisition numbers can be found in the methods section. Click here for file
