# Supplementary material for: The molecular basis of color vision in colorful fish: Four Long Wave-Sensitive (LWS) opsins in guppies (Poecilia reticulata) are defined by amino acid substitutions at key functional sites
Source: BMC Evol Biol. 2008 Jul 18;8:210. doi: 10.1186/1471-2148-8-210 (PMC2527612; doi:10.1186/1471-2148-8-210)
Supplement: Additional file 1 — List of LWS primer names and sequences used for PCR, RT-PCR and qPCR. Primer names and numbers correspond to reaction conditions shown in Additional file 2. Primer numbers corresponds to amplicons shown in Additional file 3. Sequences are given in the 5'to 3' orientation. Primers were synthesized by Operon® Biotechnologies, suspended in sterile buffered TE (pH 7.0) and stored for no longer than one year at -20°C. [file 1471-2148-8-210-S1.doc]

**Additional file 1:**

| **Primer Number** | **Primer Name** | **Sequence 5’→3’** |
| --- | --- | --- |
| 1 | ForBeg | ATGGCAGAGGAATGGGGAAAAC |
| 2 | ForEnd | TTATGCAGGAGCCACAGAGG |
| 3 | Fw100 | GATCCCTTTGAAGGACCAAACT |
| 4 | Fw1a | TCTTATCAGTCTTCACCAACGG |
| 5 | Rev8 Comp | CTTCATGAACCGACAGGTGGGC |
| 6 | Rev8 | GCCCACCTGTCGGTTCATGAAG |
| 7 | Rev4 | GACCCAGGAGAAAACTATTCCAGC |
| 8 | Rev5 | CATGACTACAACCATCCTGG |
| 9 | M13F | CAGTTTCTGTGCAGGTGACAGTAG |
| 10 | M13R | CGCTGATTGTTTATTCAGGTGC |
| 11 | LWS1 IntFor | GATAAACGGAAACTTTATGGCAAATG |
| 12 | LWS1 IntRev | CATTTGCCATAAAGTTTCCGTTTATC |
| 13 | LWS2 IntFor | GTTTGTTATTACTGCCGGGACTG |
| 14 | LWS2 IntRev | CAGTCCCGGCAGTAATAACAAAC |
| 15 | Long Intron F1 | AGGAATTGCTGGGCTTTG |
| 16 | 4KbFwdF | GCCTATTTTGATTTTATTGTTGATATTAC |
| 17 | 4KbFwdR | CCTAGTGTCATCAGAAATACTAATCCATGTCCG |
| 18 | Guppy Gap | GAATTGTCTTGACTTGGGGTTGA |
| 19 | a/sExon2 | GATGGGTTTACAACGTCTCCACAC |
| 20 | pExon2 | GATGGGTTTACGATGTCGCAACGG |
| 21 | A180SpecExon2 | GGGTTTACAACGTCTCCACTC |
| 22 | RevA | CATCCTAGATACTTCCTTCTGGG |
| 23 | Fw1a Comp | CCGTTGGTGAAGACTGATAAGA |
