# Supplementary material for: The molecular basis of color vision in colorful fish: Four Long Wave-Sensitive (LWS) opsins in guppies (Poecilia reticulata) are defined by amino acid substitutions at key functional sites
Source: BMC Evol Biol. 2008 Jul 18;8:210. doi: 10.1186/1471-2148-8-210 (PMC2527612; doi:10.1186/1471-2148-8-210)
Supplement: Additional file 2 — Sequence data and PCR conditions for specific primer combinations used for all six species of Poeciliidae. Primer names are given with corresponding primer number from Additional file 1. Amplicons are shown in Additional file 3 along with corresponding n value. 0.5 U iProof™ DNA polymerase (BioRad®) was used for each reaction with 5× iProof™ HF Buffer, 10 mM dNTP mix, ~100 ng template DNA, 0.5 μM of both forward and reverse primers and dH2O. An Eppendorf® silver block thermal cycler was used for all PCR reactions. Each reaction included an initial denaturation of 94°C for 30 seconds and a final extension of 72°C for 600 seconds. If >1 bands were found after gel electrophoresis, expected sized amplicons were cut and purified using a QIAquick® Gel Extraction Kit. [file 1471-2148-8-210-S2.doc]

**Additional file 2:**

| **Sequence Map Number** | **Species Name** | **LWS Name** | **Individual Name** | **Clone Name** | **Forward Primer** | **Reverse Primer** | **GenBank Identifier** | **Accession Number** | **PCR Conditions** |
| --- | --- | --- | --- | --- | --- | --- | --- | --- | --- |
| #1 | *Poecilia reticulata* | Genomic S180 | All-11 | 1 | (4) Fw1a | (8) Rev5 | Guppy_LWS_S180_Seq#1-#2 | EU329428 | [94°C(10s) + 60°C(15s) + 72°C(25s)] x 35 |
| #2 | *Poecilia reticulata* | Genomic S180 | All-11 | 2 | (4) Fw1a | (8) Rev5 | // | // | [94°C(10s) + 60°C(15s) + 72°C(25s)] x 35 |
| #3 | *Poecilia reticulata* | Genomic S180 | GupF | - | (1) ForBeg | (9) M13F | Guppy_LWS_S180_Seq#3 | EU329429 | [94°C(10s) + 63°C(25s) + 72°C(50s)] x 35 |
| #4 | *Poecilia reticulata* | Genomic S180 | 4 | - | (1) ForBeg | (12) LWS1 IntRev | Guppy_LWS_S180_Seq#4 | EU329430 | [94°C(10s) + 59°C(25s) + 72°C(65s)] x 35 |
| #5 | *Poecilia reticulata* | Genomic S180 | 5 | - | (1) ForBeg | (12) LWS1 IntRev | Guppy_LWS_S180_Seq#5 | EU329431 | [94°C(10s) + 59°C(25s) + 72°C(65s)] x 35 |
| #6 | *Poecilia reticulata* | Genomic S180 | 6 | - | (1) ForBeg | (12) LWS1 IntRev | Guppy_LWS_S180_Seq#6 | EU329432 | [94°C(10s) + 59°C(25s) + 72°C(65s)] x 35 |
| #7 | *Poecilia reticulata* | Genomic S180 | 10 | - | (1) ForBeg | (12) LWS1 IntRev | Guppy_LWS_S180_Seq#7 | EU329433 | [94°C(10s) + 59°C(25s) + 72°C(65s)] x 35 |
| #8 | *Poecilia reticulata* | cDNA S180 | Adult 1 | - | (4) Fw1a | (12) LWS1 IntRev | Guppy_LWS_S180_Seq#8 | EU329434 | [94°C (6s) + 55°C(12s) + 72°C(21s)] x 35 |
| #9 | *Poecilia reticulata* | cDNA S180 | Embryo 1 | - | (19) a/sExon2 | (12) LWS1 IntRev | Guppy_LWS_S180_Seq#9 | EU329435 | [94°C(10s) + 61°C(17s) + 72°C(27s)] x 35 |
| #10 | *Poecilia reticulata* | cDNA S180 | Adult 2 | - | (1) ForBeg | (12) LWS1 IntRev | Guppy_LWS_S180_Seq#10 | EU329436 | [94°C (8s) + 55°C(13s) + 72°C(25s)] x 35 |
| #11 | *Poecilia reticulata* | Genomic A180 | d31 | - | (4) Fw1a | (8) Rev5 | Guppy_LWS_A180_Seq#11 | EU329437 | [94°C(10s) + 60°C(15s) + 72°C(25s)] x 35 |
| #12 | *Poecilia reticulata* | Genomic A180 | Allison | 1 | (4) Fw1a | (8) Rev5 | Guppy_LWS_A180_Seq#12-#16 | EU329438 | [94°C(10s) + 60°C(15s) + 72°C(25s)] x 35 |
| #13 | *Poecilia reticulata* | Genomic A180 | Allison | 2 | (4) Fw1a | (8) Rev5 | // | // | [94°C(10s) + 60°C(15s) + 72°C(25s)] x 35 |
| #14 | *Poecilia reticulata* | Genomic A180 | Allison | 3 | (4) Fw1a | (8) Rev5 | // | // | [94°C(10s) + 60°C(15s) + 72°C(25s)] x 35 |
| #15 | *Poecilia reticulata* | Genomic A180 | Allison | 4 | (4) Fw1a | (8) Rev5 | // | // | [94°C(10s) + 60°C(15s) + 72°C(25s)] x 35 |
| #16 | *Poecilia reticulata* | Genomic A180 | Allison | 5 | (4) Fw1a | (8) Rev5 | // | // | [94°C(10s) + 60°C(15s) + 72°C(25s)] x 35 |
| #17 | *Poecilia reticulata* | Genomic A180 | 6 | - | (3) Fw100 | (14) LWS2 IntRev | Guppy_LWS_A180_Seq#17 | EU329439 | [94°C(10s) + 59°C(25s) + 72°C(65s)] x 35 |
| #18 | *Poecilia reticulata* | Genomic A180 | 4 | - | (3) Fw100 | (14) LWS2 IntRev | Guppy_LWS_A180_Seq#18 | EU329440 | [94°C(10s) + 59°C(25s) + 72°C(65s)] x 35 |
| #19 | *Poecilia reticulata* | Genomic A180 | Pam | - | (3) Fw100 | (14) LWS2 IntRev | Guppy_LWS_A180_Seq#19 | EU329441 | [94°C(10s) + 59°C(25s) + 72°C(65s)] x 35 |
| #20 | *Poecilia reticulata* | Genomic A180 | 10 | - | (3) Fw100 | (14) LWS2 IntRev | Guppy_LWS_A180_Seq#20 | EU329442 | [94°C(10s) + 59°C(25s) + 72°C(65s)] x 35 |
| #21 | *Poecilia reticulata* | Genomic A180 | 5 | - | (3) Fw100 | (14) LWS2 IntRev | Guppy_LWS_A180_Seq#21 | EU329443 | [94°C(10s) + 59°C(25s) + 72°C(65s)] x 35 |
| #22 | *Poecilia reticulata* | Genomic A180 | 4 | - | (3) Fw100 | (14) LWS2 IntRev | Guppy_LWS_A180_Seq#22 | EU329444 | [94°C(10s) + 59°C(25s) + 72°C(65s)] x 35 |
| #23 | *Poecilia reticulata* | Genomic A180 | 6 | - | (1) ForBeg | (14) LWS2 IntRev | Guppy_LWS_A180_Seq#23 | EU329445 | [94°C(10s) + 59°C(25s) + 72°C(65s)] x 35 |
| #24 | *Poecilia reticulata* | cDNA A180 | Adult 1 | 1 | (3) Fw100 | (6) Rev8 | Guppy_LWS_A180_Seq#24-#27 | EU329446 | [98°C (7s) + 60°C(20s) + 72°C(28s)] x 35 |
| #25 | *Poecilia reticulata* | cDNA A180 | Adult 1 | 2 | (3) Fw100 | (6) Rev8 | // | // | [98°C (7s) + 60°C(20s) + 72°C(28s)] x 35 |
| #26 | *Poecilia reticulata* | cDNA A180 | Adult 1 | 3 | (3) Fw100 | (6) Rev8 | // | // | [98°C (7s) + 60°C(20s) + 72°C(28s)] x 35 |
| #27 | *Poecilia reticulata* | cDNA A180 | Adult 1 | 4 | (3) Fw100 | (6) Rev8 | // | // | [98°C (7s) + 60°C(20s) + 72°C(28s)] x 35 |
| #28 | *Poecilia reticulata* | Genomic P180 | Gupfb | - | (4) Fw1a | (8) Rev5 | Guppy_LWS_P180_Seq#28 | EU329447 | [94°C(10s) + 60°C(15s) + 72°C(25s)] x 35 |
| #29 | *Poecilia reticulata* | Genomic P180 | Gup100A | - | (3) Fw100 | (10) M13R | Guppy_LWS_P180_Seq#29 | EU329448 | [94°C(10s) + 63°C(25s) + 72°C(45s)] x 35 |
| #30 | *Poecilia reticulata* | Genomic P180 | 4 | - | (3) Fw100 | (14) LWS2 IntRev | Guppy_LWS_P180_Seq#30 | EU329449 | [94°C(10s) + 59°C(25s) + 72°C(65s)] x 35 |
| #31 | *Poecilia reticulata* | Genomic P180 | 7 | - | (3) Fw100 | (14) LWS2 IntRev | Guppy_LWS_P180_Seq#31 | EU329450 | [94°C(10s) + 59°C(25s) + 72°C(65s)] x 35 |
| #32 | *Poecilia reticulata* | Genomic P180 | 8 | - | (3) Fw100 | (14) LWS2 IntRev | Guppy_LWS_P180_Seq#32 | EU329451 | [94°C(10s) + 59°C(25s) + 72°C(65s)] x 35 |
| #33 | *Poecilia reticulata* | Genomic P180 | 10 | - | (3) Fw100 | (14) LWS2 IntRev | Guppy_LWS_P180_Seq#33 | EU329452 | [94°C(10s) + 59°C(25s) + 72°C(65s)] x 35 |
| #34 | *Poecilia reticulata* | Genomic P180 | Pam | - | (3) Fw100 | (14) LWS2 IntRev | Guppy_LWS_P180_Seq#34 | EU329453 | [94°C(10s) + 59°C(25s) + 72°C(65s)] x 35 |
| #35 | *Poecilia reticulata* | cDNA P180 | Adult 1 | - | (4) Fw1a | (14) LWS2 IntRev | Guppy_LWS_P180_Seq#35 | EU329454 | [94°C (6s) + 54°C(12s) + 72°C(21s)] x 35 |
| #36 | *Poecilia reticulata* | cDNA P180 | Embryo 1 | - | (20) pExon2 | (14) LWS2 IntRev | Guppy_LWS_P180_Seq#36 | EU329455 | [94°C(10s) + 55°C(17s) + 72°C(27s)] x 35 |
| #37 | *Poecilia reticulata* | cDNA P180 | Adult 2 | - | (3) Fw100 | (14) LWS2 IntRev | Guppy_LWS_P180_Seq#37 | EU329456 | [94°C (6s) + 50°C(12s) + 72°C(21s)] x 35 |
| #38 | *Poecilia reticulata* | Genomic S180r | NWA | - | (3) Fw100 | (22) RevA | Guppy_LWS_S180r_Seq#38 | EU329457 | [98°C (8s) + 60°C(20s) + 72°C(30s)] x 35 |
| #39 | *Poecilia reticulata* | cDNA S180r | JuvQ | - | (3) Fw100 | (22) RevA | Guppy_LWS_S180r_Seq#39 | EU329458 | [98°C (8s) + 60°C(20s) + 72°C(30s)] x 35 |
| #40 | *Poecilia bifurca* | Genomic S180 | 2 | - | (4) Fw1a | (8) Rev5 | Bifurca_LWS_S180_Seq#40 | EU329459 | [94°C(10s) + 60°C(15s) + 72°C(25s)] x 35 |
| #41 | *Poecilia bifurca* | Genomic S180 | 1 | - | (1) ForBeg | (12) LWS1 IntRev | Bifurca_LWS_S180_Seq#41 | EU329460 | [94°C(10s) + 59°C(25s) + 72°C(65s)] x 35 |
| #42 | *Poecilia bifurca* | Genomic A180 | 2 | 1 | (4) Fw1a | (8) Rev5 | Bifurca_LWS_A180_Seq#42-#45 | EU329461 | [94°C(10s) + 60°C(15s) + 72°C(25s)] x 35 |
| #43 | *Poecilia bifurca* | Genomic A180 | 2 | 2 | (4) Fw1a | (8) Rev5 | // | // | [94°C(10s) + 60°C(15s) + 72°C(25s)] x 35 |
| #44 | *Poecilia bifurca* | Genomic A180 | 2 | 3 | (4) Fw1a | (8) Rev5 | // | // | [94°C(10s) + 60°C(15s) + 72°C(25s)] x 35 |
| #45 | *Poecilia bifurca* | Genomic A180 | 2 | 4 | (4) Fw1a | (8) Rev5 | // | // | [94°C(10s) + 60°C(15s) + 72°C(25s)] x 35 |
| #46 | *Poecilia bifurca* | Genomic P180 | 1 | - | (15) Long Intron F1 | (8) Rev5 | Bifurca_LWS_P180_Seq#46 | EU329462 | [94°C(10s) + 66°C(13s) + 72°C(25s)] x 35 |
| #47 | *Poecilia bifurca* | Genomic P180 | 3 | - | (4) Fw1a | (14) LWS2 IntRev | Bifurca_LWS_P180_Seq#47 | EU329463 | [94°C(10s) + 59°C(25s) + 72°C(60s)] x 35 |
| #48 | *Poecilia bifurca* | Genomic P180 | 3 | - | (3) Fw100 | (14) LWS2 IntRev | Bifurca_LWS_P180_Seq#48 | EU329464 | [94°C(10s) + 59°C(25s) + 72°C(65s)] x 35 |
| #49 | *Poecilia bifurca* | Genomic P180 | 1 | - | (1) ForBeg | (14) LWS2 IntRev | Bifurca_LWS_P180_Seq#49 | EU329465 | [94°C(10s) + 59°C(25s) + 72°C(65s)] x 35 |
| #50 | *Poecilia bifurca* | Genomic S180r | 3 | - | (3) Fw100 | (22) RevA | Bifurca_LWS_S180r_Seq#50 | EU329466 | [98°C (8s) + 60°C(20s) + 72°C(30s)] x 35 |
| #51 | *Poecilia parae* | Genomic S180 | 1 | - | (3) Fw100 | (12) LWS1 IntRev | Parae_LWS_S180_Seq#51 | EU329467 | [94°C(10s) + 60°C(25s) + 72°C(55s)] x 35 |
| #52 | *Poecilia parae* | Genomic S180 | 1 | - | (1) ForBeg | (12) LWS1 IntRev | Parae_LWS_S180_Seq#52 | EU329468 | [94°C(10s) + 59°C(25s) + 72°C(65s)] x 35 |
| #53 | *Poecilia parae* | Genomic P180 | 1 | - | (3) Fw100 | (14) LWS2 IntRev | Parae_LWS_P180_Seq#53 | EU329469 | [94°C(10s) + 59°C(25s) + 72°C(65s)] x 35 |
| #54 | *Poecilia parae* | Genomic P180 | 1 | - | (1) ForBeg | (10) M13R | Parae_LWS_P180_Seq#54 | EU329470 | [94°C(10s) + 63°C(25s) + 72°C(50s)] x 35 |
| #55 | *Poecilia parae* | Genomic S180r | 2 | - | (3) Fw100 | (22) RevA | Parae_LWS_S180r_Seq#55 | EU329471 | [98°C (8s) + 60°C(20s) + 72°C(30s)] x 35 |
| #56 | *Poecilia picta* | Genomic S180 | 2 | - | (4) Fw1a | (8) Rev5 | Picta_LWS_S180_Seq#56 | EU329472 | [94°C(10s) + 60°C(15s) + 72°C(25s)] x 35 |
| #57 | *Poecilia picta* | Genomic S180 | 1 | - | (3) Fw100 | (12) LWS1 IntRev | Picta_LWS_S180_Seq#57 | EU329473 | [94°C(10s) + 60°C(25s) + 72°C(55s)] x 35 |
| #58 | *Poecilia picta* | Genomic A180 | 2 | - | (4) Fw1a | (8) Rev5 | Picta_LWS_A180_Seq#58 | EU329474 | [94°C(10s) + 60°C(15s) + 72°C(25s)] x 35 |
| #59 | *Poecilia picta* | Genomic P180 | 2 | - | (15) Long Intron F1 | (8) Rev5 | Picta_LWS_P180_Seq#59 | EU329475 | [94°C(10s) + 66°C(13s) + 72°C(25s)] x 35 |
| #60 | *Poecilia picta* | Genomic P180 | 1 | - | (3) Fw100 | (14) LWS2 IntRev | Picta_LWS_P180_Seq#60 | EU329476 | [94°C(10s) + 59°C(25s) + 72°C(65s)] x 35 |
| #61 | *Poecilia picta* | Genomic S180r | 3 | - | (3) Fw100 | (22) RevA | Picta_LWS_S180r_Seq#61 | EU329477 | [98°C (8s) + 60°C(20s) + 72°C(30s)] x 35 |
| #62 | *Xiphophorus pygmaeus* | Genomic S180 | 1 | - | (4) Fw1a | (12) LWS1 IntRev | Xiphophorus_LWS_S180_Seq#62 | EU329478 | [94°C(10s) + 59°C(25s) + 72°C(60s)] x 35 |
| #63 | *Xiphophorus pygmaeus* | Genomic P180 | 1 | 1 | (4) Fw1a | (14) LWS2 IntRev | Xiphophorus_LWS_S180_Seq#63-#64 | EU329479 | [94°C(10s) + 59°C(25s) + 72°C(60s)] x 35 |
| #64 | *Xiphophorus pygmaeus* | Genomic P180 | 1 | 2 | (4) Fw1a | (14) LWS2 IntRev | // | // | [94°C(10s) + 59°C(25s) + 72°C(60s)] x 35 |
| #65 | *Xiphophorus pygmaeus* | Genomic P180 | 1 | - | (3) Fw100 | (14) LWS2 IntRev | Xiphophorus_LWS_S180_Seq#65 | EU329480 | [94°C(10s) + 59°C(25s) + 72°C(65s)] x 35 |
| #66 | *Xiphophorus pygmaeus* | Genomic S180r | 1 | - | (3) Fw100 | (22) RevA | Xiphophorus_LWS_S180_Seq#66 | EU329481 | [98°C (8s) + 60°C(20s) + 72°C(30s)] x 35 |
| #67 | *Tomeurus gracilis* | Genomic S180 | 1 | - | (4) Fw1a | (12) LWS1 IntRev | Tomeurus_LWS_S180_Seq#67 | EU329482 | [94°C(10s) + 59°C(25s) + 72°C(60s)] x 35 |
| qPCR #S1 | *Poecilia reticulata* | cDNA S180 | Juv1 | - | (19) a/sExon2 | (12) LWS1 IntRev | Guppy_LWS_S180_qPCRS1 | EU329483 | [98°C (8s) + 60°C(20s) + 72°C(30s)] x 35 |
| qPCR #S2 | *Poecilia reticulata* | cDNA A180 | Juv1 | - | (21) A180SpecExon2 | (6) Rev8 | Guppy_LWS_A180_qPCRS2 | EU329484 | [98°C (8s) + 60°C(20s) + 72°C(30s)] x 35 |
| qPCR #S3 | *Poecilia reticulata* | cDNA P180 | Juv1 | - | (20) pExon2 | (14) LWS2 IntRev | Guppy_LWS_P180_qPCRS3 | EU329485 | [98°C (8s) + 60°C(20s) + 72°C(30s)] x 35 |
| qPCR #S4 | *Poecilia reticulata* | cDNA S180r | Juv1 | - | (3) Fw100 | (22) RevA | Guppy_LWS_S180r_qPCRS4 | EU329486 | [98°C (8s) + 60°C(20s) + 72°C(30s)] x 35 |
| - | *Poecilia reticulata* | Intergenic S180 and P180 | Gup4Kb | - | (5) Rev8 Comp | (5) Rev8 Comp | - | - | [94°C(10s) + 60°C(20s) + 72°C(95s)] x 35 |
| - | *Poecilia reticulata* | ^†^Intergenic S180 and P180 | Gup4Kb | - | (11) LWS1 IntFor | (13) LWS2 IntFor | - | - | [94°C(10s) + 59°C(20s) + 72°C(85s)] x 35 |
| - | *Poecilia reticulata* | ^§^Intergenic S180 and P180 | Gup4Kb | - | (16) 4KbFwdF | - | - | - | Direct sequencing from † clone |
| - | *Poecilia reticulata* | ^§^Intergenic S180 and P180 | Gup4Kb | - | (17) 4KbFwdR | - | - | - | Direct sequencing from † clone |
| - | *Poecilia reticulata* | ^§^Intergenic S180 and P180 | Gup4Kb | - | (18) Guppy Gap | - | - | - | Direct sequencing from † clone |
| - | *Poecilia parae* | Intergenic S180 and P180 | Par4Kb | - | (5) Rev8 Comp | (5) Rev8 Comp | - | - | [94°C(10s) + 60°C(20s) + 72°C(95s)] x 35 |
| - | *Poecilia picta* | Intergenic S180 and P180 | Pic4Kb | - | (5) Rev8 Comp | (5) Rev8 Comp | - | - | [94°C(10s) + 60°C(20s) + 72°C(95s)] x 35 |

^†^ Cloned using a pGEM®-T easy Vector System II kit (Promega).

^§^ Sequenced off of the ^†^ clone using a dye terminator cycle sequencer on a Beckman Coulter CEQ 8000 genetic analysis system (160 minutes).
