# Supplementary figures and images for: The molecular basis of color vision in colorful fish: Four Long Wave-Sensitive (LWS) opsins in guppies (Poecilia reticulata) are defined by amino acid substitutions at key functional sites
Source: BMC Evol Biol. 2008 Jul 18;8:210. doi: 10.1186/1471-2148-8-210 (PMC2527612; doi:10.1186/1471-2148-8-210)

**Additional file 3:**


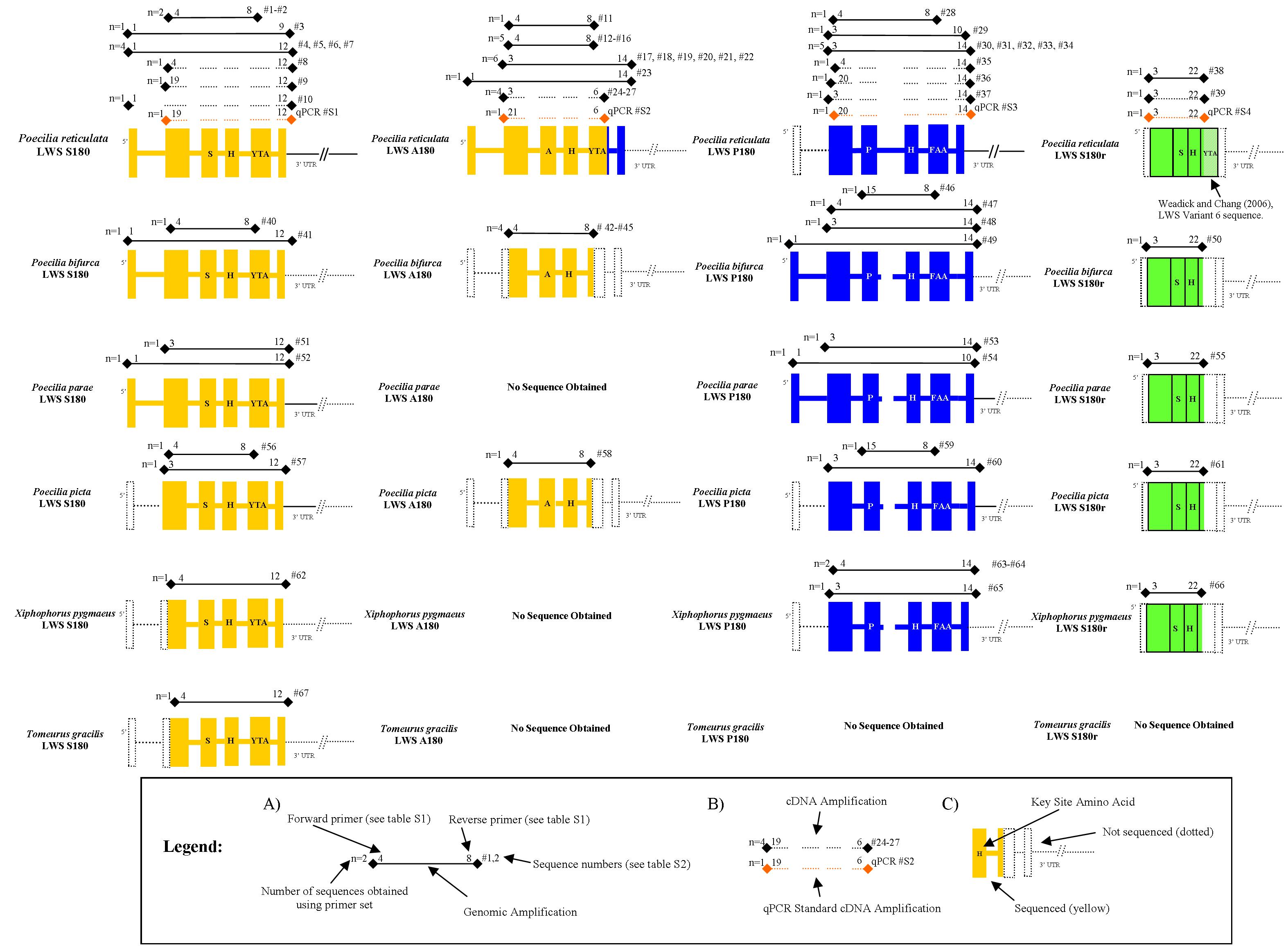

Supplement: Additional file 3 — Primer and sequence maps for LWS opsin loci in Poeciliidae. Large colored boxes and horizontal bars represent exons and introns, respectively. Black and solid horizontal lines represent genomic sequences amplified using various PCR primer combinations (see legend; see Additional files 1 and 2). Dotted horizontal lines represent mRNA transcripts acquired through RT-PCR from cDNA samples. "n" represents the number of times a given sequence of near perfect identity had been acquired. Key site amino acids are shown within their respective exons. Inter-species and individual sequence differences due to SNP's, small strings of nucleotides, or codon selection (see discussion) are omitted and a consensus map has been created. Yellow exons and introns are highly similar to the guppy LWS S180 locus. Blue coloration represents sequences of high identity to the guppy LWS P180 locus. This use of color is exploited to highlight the hybrid nature of the LWS A180 gene in the guppy. Finally, green coloration represents the diverged, single exon LWS S180r locus (originally detected by Weadick and Chang [24]). [file 1471-2148-8-210-S3.doc]
