# Supplementary material for: The molecular basis of color vision in colorful fish: Four Long Wave-Sensitive (LWS) opsins in guppies (Poecilia reticulata) are defined by amino acid substitutions at key functional sites
Source: BMC Evol Biol. 2008 Jul 18;8:210. doi: 10.1186/1471-2148-8-210 (PMC2527612; doi:10.1186/1471-2148-8-210)
Supplement: Additional file 4 — Exon alignment of LWS genes used in phylogenetic analysis. Common names are listed and acquisition numbers can be found in the methods section. [file 1471-2148-8-210-S4.doc]

**Additional file 4:**

**10 20 30 40 50 60 70 80 90 100**

**....|....|....|....|....|....|....|....|....|....|....|....|....|....|....|....|....|....|....|....|**

**Guppy LWS S180**  1 **ATGGCAGAGGAATGGGGAAAACAAGTGTTTGCTGCCAGGCGT---CACGAAGATACAACAAGAGGC---------GCTGCATTCACATACACAAACAGCA** 88

**Guppy LWS A180**  1 **ATGGCAGAGGAATGGGGAAAACAGGTGTTTGCTGCCAGGCGG---CACGAAGATACAACAAGAGGC---------GCCGCATTCACATACACAAACAGCA** 88

**Guppy LWS P180**  1 **----------------------------------------------------------------------------------------------------** 1

**Guppy LWS S180r**  1 **----------------------------------------------------------------------------------------------------** 1

**P. bifurca LWS S180**  1 **ATGGCAGAGGAATGGGGAAAACAGGTGTTTGCTGCCAGGCGG---CATGAAGATACAACAAGAGGC---------TCTGCATTCACATACACAAACAGCA** 88

**P. bifurca LWS A180**  1 **----------------------------------------------------------------------------------------------------** 1

**P. bifurca LWS P180**  1 **ATGGCAGAGGAATGGGGAAAACAGGTGTTTGCTGCCAGGCGG---CATGAAGATACAACAAGAGGC---------TCTGCATTCACATACACAAACAGCA** 88

**P. bifurca LWS S180r**  1 **----------------------------------------------------------------------------------------------------** 1

**P. parae LWS S180**  1 **ATGGCAGAGGAATGGGGAAAACAGGTGTTTGCTGCCAGGCGG---CACGAAGATACAACAAGAGGC---------TCTGCATTCACATACACAAACAGCA** 88

**P. parae LWS P180**  1 **ATGGCAGAGGAATGGGGAAAACAGGTGTTTGCTGCCAGGCGG---CACGAAGATACAACAAGAGGC---------TCTGCATTCACATACACAAACAGCA** 88

**P. Parae LWS S180r**  1 **----------------------------------------------------------------------------------------------------** 1

**P. picta LWS S180**  1 **----------------------------------------------------------------------------------------------------** 1

**P. picta LWS A180**  1 **----------------------------------------------------------------------------------------------------** 1

**P. picta LWS P180**  1 **----------------------------------------------------------------------------------------------------** 1

**P. Picta LWS S180r**  1 **----------------------------------------------------------------------------------------------------** 1

**Xiphophorus LWS S180**  1 **----------------------------------------------------------------------------------------------------** 1

**Xiphophorus LWS P180**  1 **----------------------------------------------------------------------------------------------------** 1

**Xiphophorus LWS S180r**  1 **----------------------------------------------------------------------------------------------------** 1

**Tomeurus LWS S180**  1 **----------------------------------------------------------------------------------------------------** 1

**Zebrafish LWS 1 (A180)**  1 **ATGGCAGAGCATTGGGGAGATGCAATTTATGCAGCCCGGCGAAAGGGAGATGAAACCACAAGGGAA---------GCAATGTTCACATATACCAACAGTA** 91

**Zebrafish LWS-2 (A180)**  1 **ATGGCAGAG---TGGGCCAATGCGGCATTTGCCGCGAGACGGCGAGGGGACGAAACAACAAGGGAC---------AACGCTTTCTCATATACCAACAGCA** 88

**Jap. rice fish M/LWSA (S180)**  1 **ATGGCAGAGGAGTGGGGAAAACAGGTTTTTGCTGCGAGGCGACACAATGAAGACACAACAAGAGGC---------TCTGCTTTTACTTACACAAACAGCA** 91

**Jap. rice fish M/LWSB (S180)**  1 **ATGGCAGAGCAGTGGGGAAAACAGGTTTTTGCTGCGAGGCGACAAAATGAAGACACAACAAGAGGC---------TCTGCTTTTACTTACACAAACAGCA** 91

**Bluefin killifish LWSA (S180)**  1 **ATGGCGGAGCAATGGGAAAAACAGGCTTTTGCTGCCAGGCGGTACAACGAAGAGACAACGAGGGGC---------TCTGTCTTCACATACACAAACAGTA** 91

**Bluefin killifish LWSB (S180)**  1 **ATGCTTCCAG------------------------------------------------------------------------------------------** 10

**Human LWS (S180)**  1 **ATGGCCCAGCAGTGGAGCCTCCAAAGGCTCGCAGGCCGCCATCCGCAGGACAGCTATGAGGACAGCACCCAGTCCAGCATCTTCACCTACACCAACAGCA** 100

**Human MWS (A180)**  1 **ATGGCCCAGCAGTGGAGCCTCCAAAGGCTCGCAGGCCGCCATCCGCAGGACAGCTATGAGGACAGCACCCAGTCCAGCATCTTCACCTACACCAACAGCA** 100

**Ayu smelt LWS AYU-R (S180)**  1 **ATGCAGGATGAATGGGGAAATGCAGCATTTGCTGCAAGACGGCGCAATGAAGACACAACAAGAGAG---------TCTTCATTCACTTACACCAACAGCA** 91

**Ayu smelt LWS Red-sens. (S180)** 1 **ATGACAGATGAGTGGGGAAATGCAGTGTTTGCTGCAAGACGGCGCAATGAAGACACAACAAGAGAG---------TCTTCATTCACTTACACCAACAGCA** 91

**Blind cave fish R007 (S180)**  1 **ATGGGCGACCAGTGGGGAGATGCTGTTTTCGCAGCCAGGCGACGGGGCGACGACACGACGCGAGAG---------GCAGCTTTCACCTACACCAACAGCA** 91

**Blind cave fish G101 (A180)**  1 **ATGGCCGCACACGAGCCTGTG------TTCGCCGCCCGGCGCCACAATGAAGACACCACAAGGGAG---------TCTGCATTTGTCTACACAAATGCTA** 85

**Blind cave fish G103 (A180)**  1 **ATGGCCGCACATGCCGATGAGCCTGTGTTCGCTGCCCGGCGCTACAATGAGGAAACCACAAGGGAG---------TCTGCGTTTGTTTACACAAATGCCA** 91

**Nile tilapia LWS (S180)**  1 **ATGGCAGAAGAGTGGGGAAAACAGAGTTTTGCTGCAAGGCGGTATCATGAAGATACAACCAGGGGA---------TCAGCCTTCACTTACACAAACAGCA** 91

**Fugu LWS (A180)**  1 **ATGGCAGAGGAATGGGGAAAACAGTCGTTTGCTGCCAGGCGGTACCACGAGGACACAACGAGAGGA---------TCCGCTTTCGTTTACACAAACAGCA** 91

**Pufferfish LWS (P180)**  1 **ATGGCAGAGGAATGGGGAAAACAGTCTTTCGCTGCCAGGCGGTACCATGAAGACTCAACGAGAGGA---------TCTGCTTTCGTTTACACAAACAGCA** 91

**Turbot LWS (P180)_**  1 **ATGGCAGAAGATTGGGGAAAACCGGCATTTGCTGCCAGGCGGTACCATGAGGACACAACAAGGGGG---------TCGGCCTTCATGTACACAAACAGCA** 91

**Winter flounder LWS (S180)**  1 **ATGGCAGAAGAGTGGGGGAAACAGGCGTTTGCTGCCGGGCGGTACCATGAAGATACGACAAGGGGA---------GCTGCCTTTACATATACAAACAGCA** 91

**Goldfish LWS (S180)**  1 **ATGGCAGAGCAGTGGGGAGATGCAATCTTCGCAGCTAGGCGAAGGGGAGATGAAACGACGAGGGAA---------TCAATGTTTGTATATACAAACAGCA** 91

**Coho salmon LWS (A180)**  1 **ATGGCAGAAAGCTGGGGAAGTGCTGCTTATGCAGCCAGGCGACAAAACCAAGATACAACGAGAGAA---------TCTTCCTTTACCTTCACCAACAGCA** 91

**Atlantic halibut LWS (S180)**  1 **ATGGCAGGAGCGTGGGGAAAACAGGCGTTTGCTGCCAGGCGGTACCATGAAGATACGACAAGGGGA---------TCTGCCTTTGTATACACAAACAGCA** 91

**Carp LWS (S180)**  1 **ATGGCAGAGCAGTGGGGAGACGCAATCTTTGCAGCCAGGCGAAGGGGAGATGAAACGACGAGGGAA---------ACAATGTTTGTATATACAAACAGCA** 91

**Sea Chub LWS (S180)**  1 **----------------------------------------------------------------------------------------------------** 1

**Arctic lamprey LWS (P180)**  1 **ATGACGGCGTCCTGGCATGGGGCGGTGTTCGCGGCTCGCAGGCGCAACGATGACGAAGACACAACG---AAGGATAGCATCTTTAGATACACGAACGAGA** 97

**110 120 130 140 150 160 170 180 190 200**

**....|....|....|....|....|....|....|....|....|....|....|....|....|....|....|....|....|....|....|....|**

**Guppy LWS S180**  89 **ATCATACAAAAGATCCCTTTGAAGGACCAAACTACCACATCGCTCCTCGATGGGTTTACAACGTCTCCACACTCTGGATGTGTATCGTGGTCGTTTTATC** 188

**Guppy LWS A180**  89 **ATCATACAAAAGATCCCTTTGAAGGACCAAACTACCACATCGCTCCTCGATGGGTTTACAACGTCTCCACTCTCTGGATGTGTATCGTGGTCGTTTTATC** 188

**Guppy LWS P180**  1 **------------ATCCCTTTGAAGGACCAAACTACCACATCGCTCCTCGATGGGTTTACGATGTCGCAACGGTCTGGATGTGTATCGTGGTCGTTTTATC** 88

**Guppy LWS S180r**  1 **-----------GATCCCTTTGAAGGACCAAACTACCATATCGCTCCTCGATGGGTTTACAACATCACAACAGTCTGGATGTGTTTTGTGGTCGTCTTAGC** 89

**P. bifurca LWS S180**  89 **ATCATACAAAAGATCCCTTTGAAGGACCAAACTACCACATCGCTCCTCGATGGGTTTACAACCTCTCCACACTTTGGATGTGTATCGTGGTCGTTTTATC** 188

**P. bifurca LWS A180**  1 **---------------------------------------------------------------------------------------------TCTTATC** 7

**P. bifurca LWS P180**  89 **ATCATACAAAAGATCCCTTTGAAGGACCAAACTACCACATCGCTCCTCGATGGGTTTACAACCTCTCCACACTTTGGATGTGTATCGTGGTCGTTTTATC** 188

**P. bifurca LWS S180r**  1 **-----------GATCCCTTTGAAGGACCAAACTACCACATCGCTCCTCGATGGGTTTACAACATCACAACAGTCTGGATGTGTTTTGTGGTCGTCTTATC** 89

**P. parae LWS S180**  89 **ATCATACAAAAGATCCCTTTGAAGGACCAAACTACCACATCGCTCCTCGATGGGTTTACAACCTCTCCACACTCTGGATGTGTATCGTGGTTGTTTTATC** 188

**P. parae LWS P180**  89 **ATCATACAAAAGATCCCTTTGAAGGACCAAACTACCACATCGCTCCTCGATGGGTTTACAACCTCTCCACACTCTGGATGTGTATCGTGGTTGTTTTATC** 188

**P. Parae LWS S180r**  1 **-----------GATCCCTTTGAAGGACCAAACTACCACATCGCTCCTCGATGGGTTTACAACATCACAACAGTCTGGATGTGTTTTGTGGTCGTCTTATC** 89

**P. picta LWS S180**  1 **------------ATCCCTTTGAAGGACCAAACTACCACATCGCTCCTCGATGGGTTTACAACCTCTCCACACTTTGGATGTGTATTGTGGTCGTTTTATC** 88

**P. picta LWS A180**  1 **-------------------------------------------------------------------------------------------------ATC** 3

**P. picta LWS P180**  1 **------------ATCCCTTTGAAGGACCAAACTACCACATTGCTCCTCGATGGGTTTATGATGTTGCAACAGTCTGGATGTGTATTGTGGTCGTTTTATC** 88

**P. Picta LWS S180r**  1 **-----------GATCCCTTTGAAGGACCAAACTACCACATCGCTCCTCGATGGGTTTACAACATCTCAACAGTCTGGATGTGTTTTGTGGTCGTTTTATC** 89

**Xiphophorus LWS S180**  1 **---------------------------------------------------------------------------------------------TCTTATC** 7

**Xiphophorus LWS P180**  1 **---------------------------------------------------------------------------------------------TCTTATC** 7

**Xiphophorus LWS S180r**  1 **-----------GATCCCTTTGAAGGACCAAACTACCACATCGCTCCTCGATGGGTTTACAATATAACAACAGTTTGGATGTGTTTTGTGGTCGTCTTATC** 89

**Tomeurus LWS S180**  1 **---------------------------------------------------------------------------------------------TCTTATC** 7

**Zebrafish LWS 1 (A180)**  92 **ATAACACCAAGGATCCCTTTGAGGGTCCCAATTACCACATTGCCCCTCGATGGGTGTACAATGTTGCAACAGTCTGGATGTTCTTTGTGGTTGTCGCCTC** 191

**Zebrafish LWS-2 (A180)**  89 **ATAACACAAGGGATCCCTTTGAGGGTCCCAATTACCACATTGCCCCTCGATGGGTGTACAATGTTGCAACAGTCTGGATGTTCTTTGTGGTTGTCGCCTC** 188

**Jap. rice fish M/LWSA (S180)**  92 **ATCACACCCGAGATCCTTTTGAGGGTCCTAACTACCACATTGCTCCTCGATGGGTCTACAACGTTGCCACAGTTTGGATGTTTTTTGTGGTGGTTCTGTC** 191

**Jap. rice fish M/LWSB (S180)**  92 **ATCACACTCGAGATCCCTTTGAGGGTCCTAACTACCACATTGCTCCTCGATGGGTCTACAACCTTGCCACACTTTGGATGTTTTTTGTGGTGGTTCTGTC** 191

**Bluefin killifish LWSA (S180)**  92 **ATCATACGAGAGATCCTTTTGAGGGACCAAACTACCACATCGCTCCTCGATGGGTTTACAATGTTTCGACTGTCTGGATGTTTTTCGTGGTGGTTTTATC** 191

**Bluefin killifish LWSB (S180)**  10 **------------ATCCTTTTGAGGGACCAAACTACCACATCGCTCCTCGATGGGTTTACAATGTCTCGACAGTCTGGATGTTTTTCGTGGTCATTTTATC** 98

**Human LWS (S180)**  101 **ACTCCACCAGAGGCCCCTTCGAAGGCCCGAATTACCACATCGCTCCCAGATGGGTGTACCACCTCACCAGTGTCTGGATGATCTTTGTGGTCACTGCATC** 200

**Human MWS (A180)**  101 **ACTCCACCAGAGGCCCCTTCGAAGGCCCGAATTACCACATCGCTCCCAGATGGGTGTACCACCTCACCAGTGTCTGGATGATCTTTGTGGTCATTGCATC** 200

**Ayu smelt LWS AYU-R (S180)**  92 **ACAACACTAAAGATCCGTTTGAGGGCCCTAACTACCACATTGCTCCTCGATGGGTGTACAACATCTCAACAATGTGGATGATATTCGTGGTTATTGCATC** 191

**Ayu smelt LWS Red-sens. (S180)** 92 **ACAACACTAAAGATCCATTTGAGGGCCCTAACTACCACATTGCTCCTCGATGGGTGTACAATATCTCAACAATGTGGATGATATTTGTGGTTATTGCATC** 191

**Blind cave fish R007 (S180)**  92 **ACAACACGAAAGATCCCTTTGAGGGACCCAATTACCACATTGCCCCAAGATGGGTGTACAATCTGGCTACATGCTGGATGTTCTTTGTGGTCGTCGCCTC** 191

**Blind cave fish G101 (A180)**  86 **ATAATACAAGAGATCCTTTTGAAGGACCAAACTATCACATTGCCCCTCGATGGGTCTACAACGTATCATCCTTATGGATGATCTTTGTTGTCATTGCATC** 185

**Blind cave fish G103 (A180)**  92 **ACAATACAAGAGATCCATTTGAGGGACCCAACTATCACATTGCCCCTCGATGGGTCTACAACCTAGCATCCTTATGGATGATCATTGTTGTTATCGCATC** 191

**Nile tilapia LWS (S180)**  92 **ATAACACCAGAGATCCCTTTGAGGGTCCCAATTACCACATCGCACCTCGATGGGTTTACAATCTTGCAACAGTCTGGATGTTTTTTGTGGTGATCGCGTC** 191

**Fugu LWS (A180)**  92 **ATCATACCAGAGATCCCTTCGAGGGGCCCAACTACCACATCGCTCCACGATGGGTTTACAACGTCGCGACAGTCTGGATGTTTATTGTGGTCGTCTTATC** 191

**Pufferfish LWS (P180)**  92 **ATCATACCAGAGATCCCTTCGAGGGTCCCAACTACCACATCGCTCCACGATGGGTTTACAATCTCGCCACACTCTGGATGTTTTTTGTGGTGGTCTTATC** 191

**Turbot LWS (P180)_**  92 **ACCACACCAAAGATCCCTTTGAAGGTCCCAACTACCACATTGCCCCTCGATGGATTTACAACCTTGCAACACTCTGGATGTTTGTTGTGGTCGTCGCTTC** 191

**Winter flounder LWS (S180)**  92 **ATAACACCAGAGATCCCTTTGAGGGTCCCAATTACCACATTGCTCCTCGATGGGTTTACAACCTTGCAACAGTCTGGATGTTTTTTGTGGTCGTTGCATC** 191

**Goldfish LWS (S180)**  92 **ATAACACCAGGGATCCCTTTGAGGGACCCAACTACCACATTGCTCCTCGATGGGTGTACAACCTAGCAACAGTGTGGATGTTCTTTGTGGTCGTTGCGTC** 191

**Coho salmon LWS (A180)**  92 **ATAACACCAAAGATCCCTTTGAGGGCCCCAACTACCACATTGCTCCAAGATGGGTGTACAATGTTTCAACACTTTGGATGATCATTGTGGTCATCCTCTC** 191

**Atlantic halibut LWS (S180)**  92 **ACCACACCAGAGATCCCTTTGAGGGTCCCAATTACCACATTGCTCCTCGATGGGTTTACAACATGGCAACCCTCTGGATGTTTTTTGTGGTCATTGCCTC** 191

**Carp LWS (S180)**  92 **ATAACACCAGGGATCCCTTTGAGGGACCCAACTACCACATTGCCCCTCGATGGGTGTACAACCTAGCAACAGTATGGATGTTCTTTGTGGTCATTGCCTC** 191

**Sea Chub LWS (S180)**  1 **----------------------------------------------------------------------------------------------------** 1

**Arctic lamprey LWS (P180)**  98 **ACAACACGAGAGATCCCTTCGAAGGCCCCAACTATCATATCGCCCCTCGCTGGATGTTCAACCTCACGTCCGTGTGGATGATCATCGTCGTCGTCCTCTC** 197

**210 220 230 240 250 260 270 280 290 300**

**....|....|....|....|....|....|....|....|....|....|....|....|....|....|....|....|....|....|....|....|**

**Guppy LWS S180**  189 **AGTCTTCACCAACGGCCTCGTCTTGGTGGCCACAGCAAAGTTCAAGAAACTTCGTCATCCTCTCAACTGGATCTTGGTCAACCTTGCCATTGCTGATCTT** 288

**Guppy LWS A180**  189 **AGTCTTCACCAACGGCCTCGTCTTGGTGGCCACAGCAAAGTTCAAGAAACTTCGTCATCCTCTCAACTGGATCTTGGTCAACCTTGCCATTGCTGATCTT** 288

**Guppy LWS P180**  89 **AGTCTTCACCAATGGCCTCGTTTTGGTGGCAACAGCAAAGTTCAAGAAACTTCGTCATCCTCTCAACTGGATCTTGGTCAACCTTGCCATTGCTGATCTT** 188

**Guppy LWS S180r**  90 **AGTCTTCACAAATGGTCTGGTCTTGGTAGCCACAGCAAGGTTCAAGAAACTCCGTCATCCCCTGAACTGGATCTTAGTCAACCTTGCCATTGCCGACCTC** 189

**P. bifurca LWS S180**  189 **AGTCTTCACCAATGGTCTCGTTTTGGTGGCCACAGCAAAGTTCAAGAAACTTCGTCATCCTCTCAACTGGATCTTGGTCAACCTTGCCATCGCTGATCTT** 288

**P. bifurca LWS A180**  8 **AGTCTTCACCAACGGCCTCGTTTTGGTGGCCACAGCAAAGTTCAAGAAACTTCGTCATCCTCTCAACTGGATCTTGGTCAACCTTGCCATCGCCGATCTT** 107

**P. bifurca LWS P180**  189 **AGTCTTCACCAATGGTCTCGTTTTGGTGGCCACAGCAAAGTTCAAGAAACTTCGTCATCCTCTCAACTGGATCTTGGTCAACCTTGCCATCGCTGATCTT** 288

**P. bifurca LWS S180r**  90 **AGTTTTCACAAATGGTCTGGTCTTGGTAGCCACAGCAAGGTTCAAGAAACTCCGTCATCCCCTCAACTGGATCTTAGTCAACCTTGCCATTGCCGACCTC** 189

**P. parae LWS S180**  189 **AGTCTTCACCAACGGCCTCGTTTTGGTGGCCACAGCAAAGTTCAAGAAACTTCGTCATCCTCTCAACTGGATCTTGGTCAACCTTGCCATCGCCGATCTT** 288

**P. parae LWS P180**  189 **AGTCTTCACCAACGGCCTCGTTTTGGTGGCCACAGCAAAGTTCAAGAAACTTCGTCATCCTCTCAACTGGATCTTGGTCAACCTTGCCATCGCCGATCTT** 288

**P. Parae LWS S180r**  90 **AGTCTTCACAAATGGTCTGGTCTTGGTAGCCACAGCAAGGTTTAAGAAACTCCGTCATCCCCTGAACTGGATCTTAGTCAACCTTGCCATTGCCGACCTC** 189

**P. picta LWS S180**  89 **AGTCTTCACCAACGGCCTCGTTTTGGTGGCCACAGCAAAGTTCAAGAAACTTCGTCATCCTCTCAACTGGATCTTGGTCAACCTTGCCATCGCCGATCTT** 188

**P. picta LWS A180**  4 **AGTCTTCACCAACGGCCTCGTCTTGGTGGCCACAGCAAAGTTCAAGAAACTTCGTCATCCTCTCAACTGGATCTTGGTCAACCTTGCCATTGCTGATCTT** 103

**P. picta LWS P180**  89 **AGTCTTCACCAACGGCCTCGTTTTGGTGGCCACAGCAAAGTTCAAGAAACTTCGTCATCCTCTCAACTGGATCTTGGTCAACCTTGCCATTGCTGATCTT** 188

**P. Picta LWS S180r**  90 **AGTCTTCACAAATGGTCTGGTCTTGGTAGCCACAGCAAGGTTCAAGAAACTCCGTCATCCCCTGAACTGGATCTTAGTCAACCTTGCCATTGCCGACCTC** 189

**Xiphophorus LWS S180**  8 **AGTCTTCACCAACGGCCTCGTCTTGGTGGCCACAGCAAAGTTCAAGAAACTTCGTCATCCTCTCAACTGGATCTTGGTCAACCTTGCCATTGCTGATCTT** 107

**Xiphophorus LWS P180**  8 **AGTCTTCACCAACGGCCTCGTCTTGGTGGCCACAGCAAAGTTCAAGAAACTTCGTCATCCTCTCAACTGGATCTTGGTCAACCTTGCCGTTGCTGATCTT** 107

**Xiphophorus LWS S180r**  90 **AGTCTTCACAAATGGCCTGGTCTTGGCAGCCACAGCAAAGTTCAAGAAACTCCGTCATCCCCTCAACTGGATCTTAGTCAACCTTGCCATTGCAGATCTC** 189

**Tomeurus LWS S180**  8 **AGTCTTCACCAACGGCCTCGTCTTGGTGGCCACGGCCAAGTTCAAGAAACTTCGTCACCCTCTCAATTGGATCTTGGTCAACCTTGCCATTGCTGATCTC** 107

**Zebrafish LWS 1 (A180)**  192 **AACCTTCACCAATGGCCTGGTACTGGTGGCCACGGCCAAATTCAAGAAGCTCCGTCACCCTCTCAACTGGATCTTGGTCAACCTTGCTATAGCTGATCTG** 291

**Zebrafish LWS-2 (A180)**  189 **AACCTTCACCAATGGCCTGGTACTGGTGGCCACGGCCAAATTTAAGAAGCTCCGTCACCCTCTCAATTGGATCTTGGTCAACCTTGCTATAGCTGATCTG** 288

**Jap. rice fish M/LWSA (S180)**  192 **CGTCTTCACCAACGGCCTGGTCTTGGTGGCCACAGCAAAGTTCAAGAAACTGCGCCACCCACTAAACTGGATTTTGGTCAATCTTGCTATAGCTGACCTG** 291

**Jap. rice fish M/LWSB (S180)**  192 **CGTCTTCACCAACGGCCTGGTCTTGGTGGCCACAGCAAAGTTCAAGAAACTGCGCCACCCACTAAACTGGATTTTGGTCAATCTTGCCATAGCTGACCTG** 291

**Bluefin killifish LWSA (S180)**  192 **AGTCTTCACCAATGGCCTTGTCTTGGTGGCCACGGCAAAATTCGAGAAGCTTCGTCATCCCCTCAACTGGATCTTGGTCAACCTTGCCATTGCTGATCTT** 291

**Bluefin killifish LWSB (S180)**  99 **AGTCTTCACCAACGGCCTGGTCTTAGTAGCCACAGCAAGGTTCAAGAAACTCCGTCACCCTCTCAACTGGATCCTGGTCAACCTTGCCATTGCTGATCTC** 198

**Human LWS (S180)**  201 **CGTCTTCACAAATGGGCTTGTGCTGGCGGCCACCATGAAGTTCAAGAAGCTGCGCCACCCGCTGAACTGGATCCTGGTGAACCTGGCGGTCGCTGACCTA** 300

**Human MWS (A180)**  201 **CGTTTTCACAAATGGGCTTGTGCTGGCGGCCACCATGAAGTTCAAGAAGCTGCGCCACCCGCTGAACTGGATCCTGGTGAACCTGGCGGTCGCTGACCTG** 300

**Ayu smelt LWS AYU-R (S180)**  192 **CGTCTTCACCAACGGCCTGGTACTGGTGGCCACAGCTAAATTCAAGAAGCTTCAGCACCCTCTGAACTGGATCTTGGTCAACCTGGCTATTGCTGATCTT** 291

**Ayu smelt LWS Red-sens. (S180)** 192 **CGTCTTCACCAACGGCCTGGTATTGGTGGCCACAGCTAAATTCAAGAAGCTTCAGCACCCTCTGAACTGGATCTTGGTCAACCTGGCTATTGCTGATCTT** 291

**Blind cave fish R007 (S180)**  192 **AACCGTGACCAACGGCCTGGTGCTGGTGGCCTCAGCCAAATTCAAGAAACTGCGTCACCCTCTGAACTGGATCCTGGTGAATCTCGCTATAGCTGATCTG** 291

**Blind cave fish G101 (A180)**  186 **AGTCTTCACTAATGGTTTGGTAATTGTAGCAACAGCAAAGTTCAAGAAGCTGCAACACCCTCTAAACTGGATTCTGGTAAACCTGGCTATAGCCGATCTC** 285

**Blind cave fish G103 (A180)**  192 **AATCTTCACTAACAGTCTGGTAATTGTAGCTACAGCAAAGTTCAAGAAGCTGCGACACCCTCTAAACTGGATTCTGGTAAACCTGGCTATAGCCGATCTT** 291

**Nile tilapia LWS (S180)**  192 **AGTCTTCACCAACGGTCTCGTCTTAGTGGCCACATGGAAGTTCAAGAAACTCCGTCACCCTCTAAACTGGATCTTGGTCAATCTAGCCATTGCTGATCTG** 291

**Fugu LWS (A180)**  192 **TGTCTTCACCAACGGTCTCGTGTTGGTGGCCACTGCAAAGTTCAAGAAACTCCGTCATCCTCTAAACTGGATCTTGGTCAATCTTGCCATTGCTGATCTT** 291

**Pufferfish LWS (P180)**  192 **CGTCTTCACCAACGGGCTGGTGTTGGTGGCCACCGCAAAGTTCAAGAAACTGCGTCATCCTCTAAACTGGATCTTGGTCAACCTCGCCGTTGCTGATCTT** 291

**Turbot LWS (P180)_**  192 **CGTCTTCACCAACGGTCTCGTCTTGGTGGCCACGGCAAAGTTCAAGAAACTCCGACACCCACTGAACTGGATCTTGGTAAATCTTGCAATTGCTGATCTC** 291

**Winter flounder LWS (S180)**  192 **AGTCTTCACAAATGGTCTTGTCTTGGTGGCCACAGCTAAGTTCAAGAAACTACGTCACCCACTGAACTGGATCTTGGTCAATCTTGCAATCGCTGATCTT** 291

**Goldfish LWS (S180)**  192 **GACCTTCACCAATGGCCTGGTGCTGGTGGCCACAGCCAAATTTAAGAAGCTCCGTCACCCTCTTAACTGGATCCTGGTCAACCTCGCTGTAGCTGATCTA** 291

**Coho salmon LWS (A180)**  192 **AGTCTTCACCAATGGCCTGGTACTGGTGGCCACTGCAAAATTCGAGAAGCTCCAACACCCTCTGAACTGGATCTTGGTCAACCTTGCTATTGCTGACATT** 291

**Atlantic halibut LWS (S180)**  192 **AGTCTTCACAAATGGTCTTGTCTTGGTGGCCACAGCTAAGTTCAAGAAACTCCGTCACCCACTGAACTGGATCTTGGTCAATCTGGCAATCGCTGATCTT** 291

**Carp LWS (S180)**  192 **AACCTTCACCAATGGCCTGGTGCTGGTGGCCACAGCCAAATTTAAGAAGCTCCGTCACCCTCTTAACTGGATCCTGGTCAACCTCGCTATAGCTGATCTA** 291

**Sea Chub LWS (S180)**  1 **--------------------------------------------------------------------GGATCTTGGTGAATCTTGCCATTGCCGATCTT** 32

**Arctic lamprey LWS (P180)**  198 **GCTGTTCACCAACGGCCTGGTGCTCGTGGCCACCATGAAGTTCAAGAAGCTGCGGCATCCGCTCAACTGGATCCTCGTCAACCTGGCCATTGCCGACATC** 297

**310 320 330 340 350 360 370 380 390 400**

**....|....|....|....|....|....|....|....|....|....|....|....|....|....|....|....|....|....|....|....|**

**Guppy LWS S180**  289 **GGAGAGACTGTCTTTGCCAGTACCATCAGTGTGTGCaACCAGTTCTTTGGATATTTCATTCTGGGACACCCAATGTGTGTCTTTGAAGGCTTTGTTGTCT** 388

**Guppy LWS A180**  289 **GGAGAGACAGTCTTTGCCAGCACCATCAGTGTGTGCAACCAGTTCTTTGGATATTTCATTCTGGGACACCCAATGTGTGTCTTTGAAGGCTATGTTGTCT** 388

**Guppy LWS P180**  189 **GGAGAGACTGTCTTTGCCAGCACCATCAGTGTGTGCAACCAGTTCTTTGGATATTTCATTCTGGGACACCCAATGTGTGTCTTTGAAGGCTATGTTGTCT** 288

**Guppy LWS S180r**  190 **GGAGAGACGGTCTTTGCCAGCACCATCAGCGTGTGCAACCAGTTTTTTGGATATTTTATTTTGGGACATCCAATGTGCGTCTTTGAAGGCTACGTTGTCT** 289

**P. bifurca LWS S180**  289 **GGAGAGACAGTCTTTGCCAGTACCATCAGTGTGTGC-ACCAGTTCTTTGGATATTTCATTCTGGGACACCCAATGTGTGTCTTTGAAGGCTTTACTGTCT** 387

**P. bifurca LWS A180**  108 **GGAGAGACAGTCTTTGCCAGTACCATCAGTGTGTGCAACCAGTTCTTTGGATATTTCATTCTGGGACACCCAATGTGTGTCTTTGAAGGGTATACAGTCT** 207

**P. bifurca LWS P180**  289 **GGAGAGACAGTCTTTGCCAGTACCATCAGTGTGTGCaACCAGTTCTTTGGATATTTCATTCTGGGACACCCAATGTGTGTCTTTGAAGGCTATGTTGTCT** 388

**P. bifurca LWS S180r**  190 **GGAGAGACGGTCTTTGCCAGCACCATCAGTGTGTGCAACCAGTTTTTTGGATATTTTATTTTGGGACATCCAATGTGCGTCTTTGAAGGCTACGTCGTCT** 289

**P. parae LWS S180**  289 **GGAGAGACAGTCTTTGCCAGTACCATCAGTGTGTGCaACCAGTTCTTTGGATATTTCATTCTGGGACACCCAATGTGTGTCTTTGAAGGCTTTACTGTCT** 388

**P. parae LWS P180**  289 **GGAGAGACAGTCTTTGCCAGTACTATCAGTGTGTGCAACCAGGTCTTTGGATATTTCATTCTGGGACACCCAATGTGTGTCTTTGAAGGCTATGTTGTCT** 388

**P. Parae LWS S180r**  190 **GGAGAGACGGTCTTTGCCAGCACCATCAGCGTGTGCAACCAGTTTTTTGGATATTTTATTTTGGGACATCCAATGTGCGTCTTTGAAGGCTACATCGTCT** 289

**P. picta LWS S180**  189 **GGAGAGACAGTCTTTGCCAGTACCATCAGTGTGTGCAACCAGTTCTTTGGATATTTCATTCTGGGACACCCAATGTGTGTCTTTGAAGGCTTTACTGTCT** 288

**P. picta LWS A180**  104 **GGAGAGACAGTCTTTGCCAGCACCATCAGTGTGTGCAACCAGTTCTTTGGATATTTCATTCTGGGACACCCAATGTGTGTCTTTGAAGGCTATGTTGTCT** 203

**P. picta LWS P180**  189 **GGAGAGACAGTCTTTGCCAGTACCATCAGTGTGTGCAACCAGGTCTTTGGATATTTCATTCTGGGACACCCAATGTGTGTCTTTGAAGGCTATGTTGTCT** 288

**P. Picta LWS S180r**  190 **GGAGAGACGGTCTTTGCCAGCACCATCAGCGTGTGCAACCAGTTTTTTGGATATTTTATTTTGGGACATCCAATGTGTGTCTTTGAAGGCTACGTCGTCT** 289

**Xiphophorus LWS S180**  108 **GGAGAGACAGTCTTTGCCAGCACCATCAGTGTGTGCAACCAGTTCTTTGGATATTTTATTCTGGGACACCCGATGTGTGTCTTCGAAGGCTATGTTGTCT** 207

**Xiphophorus LWS P180**  108 **GGAGAGACAGTCTTTGCCAGCACCATCAGTGTGTGCAACCAGTTCTTTGGATATTTTATTCTGGGACACCCGATGTGTGTCTTCGAAGGCTATGTTGTCT** 207

**Xiphophorus LWS S180r**  190 **GGAGAGACGGTTTTTGCCAGCACCATAAGCGTGTGCAACCAGTTTTTTGGATACTTTATTTTGGGACATCCAATGTGCGTCTTTGAAGGCTACATTGTCT** 289

**Tomeurus LWS S180**  108 **GGGGAGACGGTCTTTGCCAGCACCATCAGTGTGTGCAACCAGTTCTTTGGCTACTTCATCCTGGGACACCCAATGTGTGTCTTTGAAGGCTTTGTTGTCT** 207

**Zebrafish LWS 1 (A180)**  292 **GGAGAGACTCTGTTTGCCAGCACAATCAGTGTCATTAACCAATTTTTCGGCTACTTTATCCTAGGACATCCCATGTGTATTTTTGAAGGCTACACTGTGT** 391

**Zebrafish LWS-2 (A180)**  289 **GGAGAGACTCTGTTTGCCAGCACAATCAGCGTCATCAATCAGGTTTTCGGCTACTTTATCCTCGGACATCCCATGTGTATTTTTGAAGGCTACACTGTGT** 388

**Jap. rice fish M/LWSA (S180)**  292 **GGAGAGACGGTGTTTGCCAGCACCATCAGTGTGTGCAACCAGTTCTTTGGTTACTTCATTCTGGGACACCCCATGTGCGTGTTTGAGGGCTATGTGGTCT** 391

**Jap. rice fish M/LWSB (S180)**  292 **GGAGAGACGGTGTTTGCCAGCACCATCAGTGTGTGCAACCAGTTCTTTGGTTACTTCATTCTGGGACACCCCATGTGCGTGTTTGAGGGCTATGTGGTCT** 391

**Bluefin killifish LWSA (S180)**  292 **GGAGAGACAGTCTTTGCCAGCACCATCAGTGTATGCAACCAGTTTTTTGGATATTTTATTCTGGGTCATCCAATGTGCGTCTTTGAAGGCTTCATTGTCT** 391

**Bluefin killifish LWSB (S180)**  199 **GGAGAGACAGTCTTAGCCAGCACCATCAGCGTGTGCAACCAATTTTTTGGATATTTCATTTTGGGACATCCAATGTGCGTCTTTGAAGGCTACATTGTCT** 298

**Human LWS (S180)**  301 **GCAGAGACCGTCATCGCCAGCACTATCAGCATTGTGAACCAGGTCTCTGGCTACTTCGTGCTGGGCCACCCTATGTGTGTCCTGGAGGGCTACACCGTCT** 400

**Human MWS (A180)**  301 **GCAGAGACCGTCATCGCCAGCACTATCAGCGTTGTGAACCAGGTCTATGGCTACTTCGTGCTGGGCCACCCTATGTGTGTCCTGGAGGGCTACACCGTCT** 400

**Ayu smelt LWS AYU-R (S180)**  292 **GGAGAGACTGTCTTGGCCAGCACCATCAGCGTTTGCAATCAAACATTTGGATATTTCATTCTGGGACACCCTATGTGTGTCTTTGAAGGCTACACTGTCT** 391

**Ayu smelt LWS Red-sens. (S180)** 292 **GGAGAGACTGTCTTGGCCAGCACCATCAGCGTTTGCAATCAAACATTTGGATATTTCATTCTGGGACACCCTATGTGTGTCTTTGAAGGCTACACTGTCT** 391

**Blind cave fish R007 (S180)**  292 **TTAGAGACACTATTGGCCAGCACCATCAGCGTCTGCAACCAGTTCTTCGGCTACTTCATCCTCGGACATCCAATGTGTGTATTTGAAGGCTTTACAGTTG** 391

**Blind cave fish G101 (A180)**  286 **GGGGAGACAGTTCTTGCCAGCACAATCAGTGTCATCAACCAGATCTTCGGCTACTTCATCCTTGGACACCCAATGTGCGTTTTTGAGGGGTGGACGGTGT** 385

**Blind cave fish G103 (A180)**  292 **GGGGAGACAGTTCTTGCCAGCACAATCAGTGTGTTCAACCAGGTCTTCGGCTACTTTGTCCTTGGACACCCAATGTGCATTTTTGAGGGATGGACGGTGT** 391

**Nile tilapia LWS (S180)**  292 **GGAGAGACTGTTTTTGCCAGCACCATCAGTGTATGCAACCAGTTTTTTGGCTACTTCATTCTGGGACACCCAATGTGTGTCTTTGAAGGCTTTACTGTCT** 391

**Fugu LWS (A180)**  292 **GGAGAGACAGTTTTTGCCAGCACTATTAGCGTATGCAATCAGTTTTTTGGCTACTTTATTCTGGGACATCCAATGTGCGTCTTTGAGGGTTACACTGTCT** 391

**Pufferfish LWS (P180)**  292 **GGGGAGACACTTTTTGCCAGCACCATTAGCGTGTGCAATCAGTTTTTCGGCTACTTCATCCTGGGACACCCAATGTGCATCTTCGAGGGCTACGTGGTCT** 391

**Turbot LWS (P180)_**  292 **GGAGAAACCGTTTTTGCCAGCACCATCAGTGTATGCAATCAGTTCTTTGGTTACTTCATTCTGGGACACCCAATGTGCGTCTTTGAGGGCTATACTGTCT** 391

**Winter flounder LWS (S180)**  292 **GGAGAGACAGTTTTTGCCAGCACCATTAGTGTATGCAACCAGTTTTTTGGTTACTTCATTCTGGGACACCCGATGTGCGTCTTTGAGGGCTATACTGTCT** 391

**Goldfish LWS (S180)**  292 **GCAGAGACACTTTTGGCCAGCACCATCAGTGTCACCAACCAGTTTTTTGGCTACTTTATCCTCGGACACCCCATGTGTATTTTTGAAGGCTTCACCGTGT** 391

**Coho salmon LWS (A180)**  292 **GGAGAAACACTTTTGGCAAGCACCATCAGCGTTTGCAACCAGTTTTTTGGCTACTTCATTCTGGGACATCCAATGTGTGTATTTGAGGGATACACTGTCT** 391

**Atlantic halibut LWS (S180)**  292 **GGAGAGACAGTTTTTGCCAGCACCATTAGTGTATGCAACCAGTTTTTTGGTTACTTCATTCTGGGACACCCAATGTGCATCTTTGAGGGCTATACTGTCT** 391

**Carp LWS (S180)**  292 **GCAGAGACTCTTTTGGCCAGCACCATCAGTGTCATCAACCAGATTTTTGGCTACTTTATCCTCGGACACCCCATGTGTATTTTTGAAGGCTACACCGTGT** 391

**Sea Chub LWS (S180)**  33 **GGAGAGACAGTTTTTGCCAGCACCATCAGTGTATGCAACCAGTTTTTTGGTTACTTCATTCTGGGACACCCAATGTGTGTCTTTGAGGGCTTCACTGTCT** 132

**Arctic lamprey LWS (P180)**  298 **CTAGAGACCATCTTCGCCTCCACCATCAGCGTGTGCAACCAGGTCTTCGGATACTTCATCCTGGGTCACCCGATGTGCGTCTTCGAGGGCTACGTTGTGT** 397

**410 420 430 440 450 460 470 480 490 500**

**....|....|....|....|....|....|....|....|....|....|....|....|....|....|....|....|....|....|....|....|**

**Guppy LWS S180**  389 **CAACTTGTGGTATTGCTGCTCTATGGTCCCTGACTATCATCTCTTGGGAGAGATGGATAGTTGTGTGCAA-CCTTTTGGAAATGTCAAGTTCGATGCCAA** 487

**Guppy LWS A180**  389 **CAACTTGTGGTATTGCTGCTCTATGGTCCCTGACTATTATCTCTTGGGAGAGATGGATAGTTGTGTGCAAACCTTTTGGAAATGTCAAGTTCGATGCCAA** 488

**Guppy LWS P180**  289 **CAATTTGTGGAATTGCTGGGCTTTGGTCCCTGACTATCATCTCTTGGGAGAGATGGATAGTTGTGTGCAAACCCTTTGGAAATGTCAAGTTTGATGCCAA** 388

**Guppy LWS S180r**  290 **CGACTTGTGGTATTGCTGCTCTTTGGTCCCTGACTGTCATCTCTTGGGAGAGATGGATTGTTGTGTGCAAACCTTTTGGAAATACAAAGTTTGATGCCAA** 389

**P. bifurca LWS S180**  388 **CAACTTGTGGTATTGCTGCTCTGTGGTCCCTGACCATCATCTCTTGGGAGAGATGGGTAGTTGTTTGCAAACCtTTTGGAAATGTCAAGTTTGATGAAAA** 487

**P. bifurca LWS A180**  208 **CAACTTGTGGTATTGCTGCTCTGTGGTCCCTGACCATCATTTCTTGGGAGAGATGGGTAGTTGTGTGCAAACCATTTGGAAATGTCAAGTTTGATGAAAA** 307

**P. bifurca LWS P180**  389 **CAATTTGTGGAATTGCTGGGCTTTGGTCCCTGACCATCATCTCTTGGGAAAGATGGATAGTTGTGTGCAAACCCTTTGGAAATGTCAAGTTTGATTCCAA** 488

**P. bifurca LWS S180r**  290 **CGACTTGTGGCATTGCTGCTCTTTGGTCCCTGACCGTCATTTCTTGGGAGAGATGGATTGTTGTATGCAAGCCTTTTGGAAATACCAAGTTTGATGCCAA** 389

**P. parae LWS S180**  389 **CAACTTGTGGTATTGCTGCTCTGTGGTCCCTGACCATCATCTCTTGGGAGAGATGGGTAGTTGTGTGCAAACcTTTGGGAAATGTCAAGTTTGACGAAAA** 488

**P. parae LWS P180**  389 **CAATTTGTGGAATTGCTGGGCTTTGGTCCCTGACCATCATCTCTTGGGAGAGATGGATAGTTGTGTGCAAACCcTTTGGAAATGTCAAGTTTGATTCCAA** 488

**P. Parae LWS S180r**  290 **CGACTTGTGGTATTGCTGCTCTTTGGTCCCTGACCGTCATTTCTTGGGAGAGATGGATTGTTGTATGCAAGCCTTTCGGAAATACCAAGTTTGATGCCAA** 389

**P. picta LWS S180**  289 **CAACTTGTGGTATTGCTGCTCTGTGGTCCCTGACCATCATCTCTTGGGAGAGATGGGTAGTTGTGTGCAAACCTTTTGGAAATGTCAAGTTTGATGAAAA** 388

**P. picta LWS A180**  204 **CAACTTGTGGTATTGCTGCTCTATGGTACCTGACTATTATCTCTTGGGAGAGATGGATAGTTGTGTGCAAACCTTTTGGAAATGTCAAGTTCGATGCCAA** 303

**P. picta LWS P180**  289 **CAATTTGTGGAATTGCTGGGCTTTGGTCCCTGACCATCATCTCTTGGGAGAGATGGATAGTTGTGTGCAAACCCTTTGGAAATGTCAAGTTTGATTCCAA** 388

**P. Picta LWS S180r**  290 **CGACTTGTGGTATTGCTGCTCTTTGGTCCCTGACTGTCATCTCTTGGGAGAGATGGATTGTTGTATGCAAACCTTTTGGAAATACCAAGTTTGATGCCAA** 389

**Xiphophorus LWS S180**  208 **CAACTTGTGGTATTGCTGCTCTTTGGTCCCTGACTATCATCTCTTGGGAGAGATGGATAGTTGTGTGCAAACCCTTTGGAAATGTCAAGTTCGATGCCAA** 307

**Xiphophorus LWS P180**  208 **CAATTTGTGGAATTGCTGGGCTTTGGTCCCTGACTATCATCTCTTGGGAGAGATGGATAGTTGTGTGCAAACCCTTTGGAAATGTCAAGTTCGATGCCAC** 307

**Xiphophorus LWS S180r**  290 **CAACTTGTGGTATTGCTGCTCTTTGGTCTCTGACTGTCATCTCTTGGGAGAGATGGATTGTTGTATGCAAACCTTTTGGAAATACCAAGTTTGATGCCAA** 389

**Tomeurus LWS S180**  208 **CGACGTGTGGTATTGCTGCTCTTTGGTCCCTGACTATCATCTCTTGGGAAAGATGGATAGTTGTGTGCAAACCCTTTGGAAACGTCAAGTTCGATGCCAA** 307

**Zebrafish LWS 1 (A180)**  392 **CAGTATGTGGTATTGCTGCACTGTGGTCGTTGACTGTCATCTCTTGGGAAAGATGGGTGGTTGTCTGTAAACCATTTGGAAATGTCAAGTTTGATGCTAA** 491

**Zebrafish LWS-2 (A180)**  389 **CAGTATGTGGTATTGCTGGACTGTGGTCGTTGACTGTCATCTCTTGGGAAAGATGGGTGGTTGTCTGTAAACCATTTGGAAATGTCAAGTTTGATGGTAA** 488

**Jap. rice fish M/LWSA (S180)**  392 **CCACTTGTGGTATTGCTGCTCTTTGGTCTCTGACCATAATCTCCTGGGAAAGATGGGTAGTCGTGTGCAAACCATTTGGAAATGTCAAGTTTGATGCCAA** 491

**Jap. rice fish M/LWSB (S180)**  392 **CCACTTGTGGTATTGCTGCTCTTTGGTCTCTGACCATAATCTCCTGGGAAAGATGGGTAGTCGTGTGCAAACCTTTTGGAAATGTCAAGTTTGATGCCAA** 491

**Bluefin killifish LWSA (S180)**  392 **CAACTTGTGGTATTGCTGCTCTTTGGTCCCTGACTATCATCTCGTGGGAGAGGTGGATAGTTGTGTGCAAACCTTTTGGAAATGTCAAGTTTGATGCCAA** 491

**Bluefin killifish LWSB (S180)**  299 **CCACTTGTGGTATAGCTGCACTTTGGTCCCTGACTATCATCTCTTGGGAGAGATGGATAGTTGTATGCAAACCTTTTGGAAATGCCAAGTTCGATGGCAC** 398

**Human LWS (S180)**  401 **CCCTGTGTGGGATCACAGGTCTCTGGTCTCTGGCCATCATTTCCTGGGAGAGGTGGCTGGTGGTGTGCAAGCCCTTTGGCAATGTGAGATTTGATGCCAA** 500

**Human MWS (A180)**  401 **CCCTGTGTGGGATCACAGGTCTCTGGTCTCTGGCCATCATTTCCTGGGAGAGATGGATGGTGGTCTGCAAGCCCTTTGGCAATGTGAGATTTGATGCCAA** 500

**Ayu smelt LWS AYU-R (S180)**  392 **CTACTTGTGGTATTGCTGCTCTGTGGTCCCTAACTGTGATTTCCTGGGAGAGATGGGTGGTTGTGTGCAAGCCTTTTGGAAATGTCAAGTTTGATGCCAA** 491

**Ayu smelt LWS Red-sens. (S180)** 392 **CTACTTGTGGTATTGCTGCTCTGTGGTCCCTAACTGTGATTTCCTGGGAGAGATGGGTGGTTGTGTGCAAGCCTTTTGGAAATGTCAAGTTTGATGCCAA** 491

**Blind cave fish R007 (S180)**  392 **CTACCTGTGGTATTGCTGGCCTGTGGTCTCTGACTGTTATCTCATGGGAGAGATGGGTGGTGGTGTGCAAGCCTTTTGGAAATGTGAAGTTTGACGGGAA** 491

**Blind cave fish G101 (A180)**  386 **CTGTCTGTGGTATCACAGCTCTGTGGTCTCTGACTATAATCTCCTGGGAGCGCTGGGTGGTTGTGTGCAAGCCATTTGGAAATGTTAAATTCGATGGCAA** 485

**Blind cave fish G103 (A180)**  392 **CTGTCTGTGGTATCACAGCTCTGTGGTCTCTGACTATAATCTCCTGGGAGCGCTGGGTGGTTGTGTGCAAGCCATTTGGTAATGTTAAATTTGATGGCAA** 491

**Nile tilapia LWS (S180)**  392 **CAACTTGTGGTATTGCTGCTCTTTGGTCCCTGACAATCATCTCCTGGGAAAGATGGATAGTTGTGTGCAAACCTTTTGGAAATGTCAAATTTGATGCCAA** 491

**Fugu LWS (A180)**  392 **CCACTTGTGGTATCGCTGCTCTCTGGTCGCTGACCATCATCTCCTGGGAGAGATGGGTCGTCGTGTGCAAGCCTTTTGGAAACGTCAAGTTCGATGCCAA** 491

**Pufferfish LWS (P180)**  392 **CCGTTTGCGGTATCGCCGGTCTCTGGTCGCTGACTATCATCTCCTGGGAGAGGTGGATAGTCGTGTGCAAGCCTTTTGGAAACGTCAAGTTTGATGCCAA** 491

**Turbot LWS (P180)_**  392 **CAGTTTGTGGAATCGCTGGTCTCTGGTCCCTCACTATTATCTCCTGGGAGAGATGGATAGTTGTGTGCAAACCTTTTGGAAACATCAAGTTTGACGCTAA** 491

**Winter flounder LWS (S180)**  392 **CAGTGTGTGGAATTGCTGCTCTCTGGTCCCTGTCTATCATCTCCTGGGAGAGATGGGTAGTTGTGTGCAAACCTTTTGGAAACGTTAAGTTTGATGCCAA** 491

**Goldfish LWS (S180)**  392 **CTGTATGTGGTATCGCTGGTCTGTGGTCTTTGACTGTCATCTCTTGGGAAAGATGGGTGGTTGTCTGCAAACCATTTGGAAATGTCAAGTTTGATGCTAA** 491

**Coho salmon LWS (A180)**  392 **CCACTTGCGGAATTGCTGCTCTGTGGTCCCTGGCTGTCATCTCTTGGGAGAGATGGGTGGTGGTGTGCAAGCCCTTTGGAAGTGTCAAGTTTGATGCCAA** 491

**Atlantic halibut LWS (S180)**  392 **CAGTGTGTGGAATTGCTGCTCTCTGGTCCCTGTCCATCATCTCCTGGGAGAGATGGGTAGTTGTATGCAAACCTTTTGGAAACGTCAAGTTTGATGCCAA** 491

**Carp LWS (S180)**  392 **CTGTATGTGGTATCGCTGGTCTGTGGTCTTTGACTGTCATCTCTTGGGAAAGATGGGTGGTCGTCTGCAAACCATTTGGAAATGTCAAGTTTGATGCTAA** 491

**Sea Chub LWS (S180)**  133 **CAACTTGTGGTATTGCTGCTCTCTGGTCCCTGACCATCATCTCCTGGGAGAGATGGATAGTTGTGTGCAAACCTTTTGGAAATGTCAAGTTTGGTGCCAA** 232

**Arctic lamprey LWS (P180)**  398 **CCACTTGCGGCATTGCCGGTCTGTGGTCGCTAGCCATCATCTCGTGGGAGCGCTGGATGGTCGTCTGCAAACCCTTCGGCAACATCAAGTTCGACGGCAA** 497

**510 520 530 540 550 560 570 580 590 600**

**....|....|....|....|....|....|....|....|....|....|....|....|....|....|....|....|....|....|....|....|**

**Guppy LWS S180**  488 **GTGGGCCACAGGTGGAATAGTTTTCTCCTGGGTCTGGTCTGCAGCGTGGTGCGCACCTCCCATCTTTGGATGGAGCAGGTTTTGGCCTCATGGACTGAAA** 587

**Guppy LWS A180**  489 **GTGGGCCACAGCTGGAATAGTTTTCTCCTGGGTCTGGGCTGCAGTGTGGTGCGCTCCTCCCATCTTTGGATGGAGCAGGTATTGGCCTCATGGACTGAAA** 588

**Guppy LWS P180**  389 **GTGGGCCACAGCTGGAATACTTTTCTCCTGGGTCTGGCCTGCAGTGTGGTGCGCTCCTCCCATCTTTGGATGGAGCAGGTATTGGCCTCATGGACTGAAA** 488

**Guppy LWS S180r**  390 **ATGGGCGGCAGCTGGGATCATGTTCTCCTGGGTCTGGTCGGCAGTGTGGTGTGCTCCTCCCGTCTTTGGATGGAGCAGGTACTGGCCCCATGGGTTGAAA** 489

**P. bifurca LWS S180**  488 **GTGGGCCACGGGTGGAATAGTTTTCTCCTGGGTCTGGTCTGCAGCGTGGTGCGCTCCTCCCATCTTTGGATGGAGCAGGTTTTGGCCTCATGGACTGAAA** 587

**P. bifurca LWS A180**  308 **GTGGGCCACGGCTGGAATAGTTTTCTCCTGGGTCTGGGCTGCAGCGTGGTGCGCTCCTCCCATCTTTGGATGGAGCAGGTA-TGGCCTCATGGACTGAAA** 406

**P. bifurca LWS P180**  489 **GTGGGCCACAGCTGGAATACTTTTCTCCTGGGTCTGGCCTGCAGTGTGGTGCGCTCCTCCCATCTTTGGATGGAGCAGGTATTGGCCTCATGGACTGAAA** 588

**P. bifurca LWS S180r**  390 **ATGGGCCACAGCTGGGATCGTGTTCTCCTGGGTCTGGTCGGCAGTGTGGTGTGCTCCTCCCGTCTTTGGATGGAGCAGGTACTGGCCCCATGGGTTGAAA** 489

**P. parae LWS S180**  489 **GTGGGCCACGGCTGGAATAGTTTTCTCCTGGGTCTGGTCTGCAGCGTGGTGCGCTCCTCCCATCTTTGGATGGAGCAGGTTTTGGCCTCATGGACTGAAA** 588

**P. parae LWS P180**  489 **GTGG-CCTCAGCTGGAATACTTTTCTCCTGGGTCTGGCCTGCAGTGTGGTGCGCTCCTCCCATCTTTGGATGGAGCAGGTATTGGCCTCATGGACTAAAA** 587

**P. Parae LWS S180r**  390 **ATGGGCCGCAGCTGGGATCGTGTTCTCCTGGGTCTGGTCGGCAGTGTGGTGTGCTCCTCCCGTCTTTGGATGGAGCAGGTACTGGCCCCATGGGTTGAAA** 489

**P. picta LWS S180**  389 **GTGGGCCACAGCTGGAATAGTTTTCTCCTGGGTCTGGTCTGCAGCATGGTGCGCTCCTCCCATCTTTGGATGGAGCAGGTTTTGGCCTCATGGACTGAAA** 488

**P. picta LWS A180**  304 **GTGGGCCACAGCTGGAATAGTTTTCTCCTGGGTCTGGGCTGCAGTGTGGTGCGCTCCTCCCATCTTTGGATGGAGCAGGTA-TGGCCTCATGGACTGAAA** 402

**P. picta LWS P180**  389 **GTGGGCCACAGCTGGAATACTTTTCTCCTGGGTCTGGCCTGCAGTGTGGTGCGCTCCTCCCATCTTTGGATGGAGCAGGTATTGGCCTCATGGACTGAAA** 488

**P. Picta LWS S180r**  390 **ATGGGCCACAGGTGGGATCGTGTTCTCCTGGGTCTGGTCGGCAGTGTGGTGTGCTCCTCCCGTCTTTGGATGGAGCAGGTACTGGCCCCATGGGTTGAAA** 489

**Xiphophorus LWS S180**  308 **GTGGGCCACAGCTGGAATAGTTTTCTCCTGGGTCTGGTCTGCGGTGTGGTGTGCTCCTCCCATCTTTGGATGGAGCAGGTATTGGCCTCATGGACTGAAA** 407

**Xiphophorus LWS P180**  308 **GTGGGCCACAGCTGGAATAGTTTTCTCCTGGGTCTGGCCTGCAGTGTGGTGTGCTCCTCCCATCTTTGGATGGAGCAGGTATTGGCCTCATGGACTGAAA** 407

**Xiphophorus LWS S180r**  390 **ATGGGCCACAGCTGGGATCATGTTCTCCTGGGTCTGGTCGGCAGTGTGGTGTGCTCCTCCTGTCTTTGGATGGAGCAGGTACTGGCCTCATGGATTGAAA** 489

**Tomeurus LWS S180**  308 **GTGGGCCACAGCCGGCATAGTCTTCTCCTGGGTCTGGTCCGCCGTGTGGTGTGCTCCACCCATCTTTGGATGGAGCAGGTACTGGCCTCACGGACTGAAA** 407

**Zebrafish LWS 1 (A180)**  492 **ATGGGCTTCTGCTGGCATTATCTTCTCCTGGGTTTGGGCTGCTGCTTGGTGTGCACCTCCCATCTTTGGCTGGAGCAGATACTGGCCTCATGGTCTGAAG** 591

**Zebrafish LWS-2 (A180)**  489 **ATGGGCATCTGCTGGCATTATCTTCTCCTGGGTTTGGGCTGCTGTTTGGTGTGCACCTCCCATCTTTGGCTGGAGCAGGTATTGGCCTCATGGTCTGAAG** 588

**Jap. rice fish M/LWSA (S180)**  492 **GTGGGCCATAGGTGGAATCGTCTTCTCCTGGGTCTGGTCAGCAGTTTGGTGTGCACCTCCCGTCTTTGGATGGAGCAGGTACTGGCCTCATGGACTAAAA** 591

**Jap. rice fish M/LWSB (S180)**  492 **GTGGGCCATAGGTGGAATCGTCTTCTCCTGGGTCTGGTCTGCAGTTTGGTGTGCACCTCCCGTCTTTGGATGGAGCAGGTACTGGCCTCATGGACTAAAA** 591

**Bluefin killifish LWSA (S180)**  492 **ATGGGCCACAGCTGGAATCGTGTTCTCCTGGGTCTGGTCTGCAGTGTGGTGTGCTCCTCCCATCTTTGGATGGAGCAGGTATTGGCCCCATGGACTGAAA** 591

**Bluefin killifish LWSB (S180)**  399 **ATGGGCCACCGCAGGAATTATGTTCTCCTGGCTCTGGTCTGCAGTGTGGTGTGCTCCTCCCATCTTTGGATGGAGCAGGTATTGGCCCCATGGACTGAAA** 498

**Human LWS (S180)**  501 **GCTGGCCATCGTGGGCATTGCCTTCTCCTGGATCTGGTCTGCTGTGTGGACAGCCCCGCCCATCTTTGGTTGGAGCAGGTACTGGCCCCACGGCCTGAAG** 600

**Human MWS (A180)**  501 **GCTGGCCATCGTGGGCATTGCCTTCTCCTGGATCTGGGCTGCTGTGTGGACAGCCCCGCCCATCTTTGGTTGGAGCAGGTACTGGCCCCACGGCCTGAAG** 600

**Ayu smelt LWS AYU-R (S180)**  492 **ATGGGCAACTGGCGGCATTGTCTTCTCCTGGGTCTGGTCAGCAGCCTGGTGTGCTCCCCCAGTCTTTGGCTGGAGCAGGTATTGGCCTCATGGCTTGAAG** 591

**Ayu smelt LWS Red-sens. (S180)** 492 **ATGGGCCACTGCCGGCATTGTCTTCTCCTGGGTCTGGTCAGCAGCTTGGTGTGCTCCCCCAATCTTTGGCTGGAGCAGGTATTGGCCTCATGGCTTGAAG** 591

**Blind cave fish R007 (S180)**  492 **AATGGCAACTGCTGGAATTGTCTTCACCTGGGTCTGGTCTGCAGTCTGGTGCGCACCTCCCATCTTTGGATGGAGCAGGTACTGGCCTCACGGTCTGAAG** 591

**Blind cave fish G101 (A180)**  486 **ATGGGCAGCAGGTGGCATCATCTTCTCCTGGGTTTGGGCCATCATCTGGTGCACCCCTCCAATCTTTGGCTGGAGCAGGTACTGGCCCCATGGTCTGAAG** 585

**Blind cave fish G103 (A180)**  492 **ATGGGCAGCAGGAGGCATTATTTTTGCCTGGACTTGGGCCATTATCTGGTGCACCCCTCCAATCTTTGGCTGGAGCAGGTACTGGCCCCATGGTCTGAAG** 591

**Nile tilapia LWS (S180)**  492 **ATGGGCCACAGGTGGAATTGTCTTCTCCTGGGTCTGGTCTGCATTTTGGTGTGCTCCTCCCATATTTGGATGGAGCAGGTTCTGGCCTCATGGACTGAAG** 591

**Fugu LWS (A180)**  492 **ATGGGCCACAGGGGGAATTGTGTTCTCCTGGGTGTGGGCAGCAGTCTGGTGTGCCCCCCCCATCTTCGGATGGAGCAGGTACTGGCCTCATGGACTGAAG** 591

**Pufferfish LWS (P180)**  492 **ATGGGCCACGGCTGGAATCGTGTTCTCCTGGATCTGGCCCATTTGCTGGTGTGCTCCTCCAATCTTCGGATGGAGCAGGTACTGGCCTCACGGGCTGAAG** 591

**Turbot LWS (P180)_**  492 **ATGGGCCACAGGTGGAATCTTGTTCTCCTGGATCTGGCCAATAGTGTGGTGTGCACCCCCAATCTTTGGCTGGAGCAGGTACTGGCCTCATGGACTTAAG** 591

**Winter flounder LWS (S180)**  492 **ATGGGCCATGGGTGGAATTCTGTTCTCCTGGATCTGGTCAGCAGCGTGGTGTGCTCCCCCAATCTTTGGCTGGAGCAGGTACTGGCCTCATGGACTGAAG** 591

**Goldfish LWS (S180)**  492 **ATGGGCATCTGCTGGTATCATCTTCTCCTGGGTTTGGTCTGCTATCTGGTGTGCACCACCCATCTTTGGCTGGAGCAGATTCTGGCCTCATGGTCTAAAG** 591

**Coho salmon LWS (A180)**  492 **ATGGGCCATGGGAGGCATTATCTTCTCCTGGGTCTGGGCTGCTTTCTGGTGTGCCCCCCCCATCTTTGGCTGGAGCAGGTACTGGCCTCACGGCCTGAAG** 591

**Atlantic halibut LWS (S180)**  492 **ATGGGCCACAGGTGGAATTTTGTTCTCCTGGATCTGGTCAGCAGTGTGGTGTGCTCCCCCAATCTTTGGCTGGAGCAGGTACTGGCCTCATGGACTGAAG** 591

**Carp LWS (S180)**  492 **ATGGGCATCTGCTGGTATCATCTTCTCCTGGGTTTGGTCTGCTTTCTGGTGTGCACCTCCCATCTTTGGCTGGAGCAGATTCTGGCCTCACGGTCTAAAG** 591

**Sea Chub LWS (S180)**  233 **ATGGGCCACAGGTGGAATAGTGTTCTCCTGGGTCTGGTCAGCAGCGTGGTGTGCTCCCCCCATCTTTGGATGGAGCAGGTACTGGCCTCATGGACTGAAG** 332

**Arctic lamprey LWS (P180)**  498 **AATTGCCATAATCCTCATCGTCTTCTCGTGGGTCTGGCCCGCGTGTTGGTGTTCGCTTCCCATATTCGGCTGGAGCAGGTACTGGCCGCACGGGCTGAAG** 597

**610 620 630 640 650 660 670 680 690 700**

**....|....|....|....|....|....|....|....|....|....|....|....|....|....|....|....|....|....|....|....|**

**Guppy LWS S180**  588 **ACATCcTGCGGACCTGATGTGTTCAGTGGAAGCGATGACCCAGGGGTCCTGTCCTACATGATTGTCCTCATGATTACATGCTGCATCATTCCTCTGGCTA** 687

**Guppy LWS A180**  589 **ACATCCTGCGGACCTGATGTGTTCAGTGGAAGCGATGACCCAGGGGTCCTGTCCTACATGATTGTCCTCATGATTACATGCTGCATCATTCCTCTGGCTA** 688

**Guppy LWS P180**  489 **ACATCCTGTGGACCTGATGTGTTCAGTGGAAGTGAAGACCCTGGAGTCCAGTCCTACATGATTGTCCTCGTGATTACATGCTGCATCATTCCTCTGTCTA** 588

**Guppy LWS S180r**  490 **ACGTCCTGTGGACCCGATGTGTTCAGTGGAAGTGAGGACCCCGGTGTCAAGTCCTACATGATTGTCCTCATGATTACATGCTGCATCACTCCTCTGGCTG** 589

**P. bifurca LWS S180**  588 **AACtGCTGCGGACCTGATGTGTTCAGTGGAAGCGACGACCCAGGGGTCCTGTCCTACATGATTGTCCTCATGATTACTTGTTGCTTTATTCCTCTGGCTA** 687

**P. bifurca LWS A180**  407 **ACGTCCTGCGGACCTGATGTGTTCAGTGGAAGCGATGACCCAGGGGTCCTGTCCTACATGATTGTCCTCATGATTACATGCTGCATCATTCCTCTGGCTA** 506

**P. bifurca LWS P180**  589 **ACGtcCTGCGGACCTGATGTGTTCAGTGGAAGTGAAGACCCTGGAGTCCAGTCCTACATGATGGTCCTCATAATTACATGCTGCTTCATTCCTCTGGCTA** 688

**P. bifurca LWS S180r**  490 **ACATCCTGTGGACCCGATGTGTTCAGTGGAAGTGAGGACCCTGGTGTCAAGTCCTACATGATTGTCCTCATGATTACATGCTGCGTCATTCCTCTGGCTG** 589

**P. parae LWS S180**  589 **ACGtcCTGCGGACCTGATGTGTTCAGTGGAAGCGATGACCCAGGGGTCCTGTCCTACATGATTGTCCTCATGATTACTTGCTGCTTTATTCCTCTGGCTA** 688

**P. parae LWS P180**  588 **ACGTCCTGTGGACCTGATGTGTTCAGTGGAAGTGAAGACCCTGGAGTCCAGTCCTACATGATTGTCCTCATAATTACATGCTGCATCATTCCTCTGGCTA** 687

**P. Parae LWS S180r**  490 **ACATCCTGTGGACCCGATGTGTTCAGTGGAAGTGAGGACCCTGGTGTCAAGTCCTACATGATTGTCCTCATGATTACATGCTGCGTCATTCCTCTGGCTG** 589

**P. picta LWS S180**  489 **ACGTCCTGCGGACCTGATGTGTTCAGTGGAAGCGATGACCCAGGGGTCCTGTCCTACATGATTGTTCTCATGATTACATGCTGCTTCATTCCTCTGGCTA** 588

**P. picta LWS A180**  403 **ACATCCTGCGGACCTGATGTGTTCAGTGGAAGCGATGACCCAGGGGTCCTGTCCTACATGATTGTCCTCATGATTACATGCTGCATCATTCCTCTGGCTA** 502

**P. picta LWS P180**  489 **ACGTCCTGCGGACCTGATGTGTTCAGTGGAAGTGAAGACCCTGGAGTCCAGTCCTACATGATTGTCCTCATAATTACATGCTGCCTCATTCCTCTGGCTA** 588

**P. Picta LWS S180r**  490 **ACGTCCTGTGGACCCGATGTGTTCAGTGGAAGTGAGGACCCCGGTGTCAAGTCCTACATGATTGTCCTCATGATTACATGCTGCATCATTCCTCTGGCTG** 589

**Xiphophorus LWS S180**  408 **ACGTCCTGCGGGCCTGATGTGTTCAGTGGAAGTGAAGACCCTGGAGTCCAGTCCTACATGGTTGTCCTCATGATTACATGCTGCATCATTCCTCTGGCTA** 507

**Xiphophorus LWS P180**  408 **ACGTCCTGCGGGCCTGATGTGTTCAGTGGAAGTGAAGACCCTGGAGTCCAGTCCTACATGATTGTCCTCATAGTTACATGCTGCCTCACTCCTCTGGCTA** 507

**Xiphophorus LWS S180r**  490 **ACTTCCTGTGGACCCGATGTGTTCAGTGGAAGTGAGGACGCTGGTGTCAAGTCCTACATGATTGTCCTCATGATTACATGCTGCATCATTCCTCTGGCTG** 589

**Tomeurus LWS S180**  408 **ACCTCCTGCGGACCCGATGTGTTCAGCGGAAGCGATGACCCTGGAGTCCTGTCCTACATGATTGTGCTCATGATCACATGCTGCATCATTCCTCTGGCTA** 507

**Zebrafish LWS 1 (A180)**  592 **ACCTCCTGTGGCCCTGATGTCTTCAGTGGAAGCGAGGACCCTGGAGTTCAGTCCTACATGGTGGTGCTCATGATCACCTGCTGTATCATCCCTCTAGCTA** 691

**Zebrafish LWS-2 (A180)**  589 **ACCTCCTGTGGACCTGATGTGTTTGGAGGAAACGAGGACCCCGGAGTCCAGTCCTACATGCTGGTCCTAATGATCACCTGTTGCATCCTTCCTCTTGCTA** 688

**Jap. rice fish M/LWSA (S180)**  592 **ACCTCCTGTGGACCTGATGTGTTCAGTGGAAGCGATGACCCTGGAGTGCAGTCCTACATGATTGTTCTGATGATCACATGCTGCATCATTCCCCTGGCCA** 691

**Jap. rice fish M/LWSB (S180)**  592 **ACCTCCTGTGGACCTGATGTGTTCAGTGGAAGCGATGACCCTGGAGTGCAGTCCTACATGATTGTTCTGATGATCACATGCTGCATCATTCCCCTGGCCA** 691

**Bluefin killifish LWSA (S180)**  592 **ACTTCTTGTGGACCTGATGTGTTCAGTGGAAGTGAAGACCCTGGGGTCCAGTCCTACATGGTCGTCCTTATGATTACATGCTGCATCATTCCTCTGGCTA** 691

**Bluefin killifish LWSB (S180)**  499 **ACCTCCTGTGGACCTGATGTCTTCAGTGGAAGTGATGACCTTGGTGTCCTCTCCTACATGATTGTCCTCATGATTACATGCTGCATCCTTCCTTTGGCTA** 598

**Human LWS (S180)**  601 **ACTTCATGCGGCCCAGACGTGTTCAGCGGCAGCTCGTACCCCGGGGTGCAGTCTTACATGATTGTCCTCATGGTCACCTGCTGCATCATCCCACTCGCTA** 700

**Human MWS (A180)**  601 **ACTTCATGCGGCCCAGACGTGTTCAGCGGCAGCTCGTACCCCGGGGTGCAGTCTTACATGATTGTCCTCATGGTCACCTGCTGCATCACCCCACTCAGCA** 700

**Ayu smelt LWS AYU-R (S180)**  592 **ACTTCCTGTGGACCTGATGTGTTCAGTGGAAGTGATGACCCTGGAGTCAAGTCTTACATGATTGTTCTGATGGTTACCTGCTGCTTCCTTCCTCTGGCTA** 691

**Ayu smelt LWS Red-sens. (S180)** 592 **ACTTCCTGTGGACCTGATGTGTTCAGTGGAAGTGACGACCCTGGAGTCAAGTCTTACATGATTGTTCTGATGATTACCTGCTGCTTCCTTCCTCTGGCTA** 691

**Blind cave fish R007 (S180)**  592 **ACCTCCTGTGGACCTGATGTGTTCAGTGGAAGCGAAGACCCTGGAGTTCAGTCCTACATGATTGTTCTGATGATCACCTGCTGCTTTATTCCCCTGGGCA** 691

**Blind cave fish G101 (A180)**  586 **ACATCCTGTGGCCCTGATGTGTTCAGTGGCAGTGAGGATCCAGGAGTGGCCTCCTACATGATCACCCTAATGCTTACCTGCTGTATTCTTCCTCTGTCCA** 685

**Blind cave fish G103 (A180)**  592 **ACATCTTGTGGCCCTGATGTGTTCAGCGGCAGTGAGGATCCAGGAGTGGCCTCCTACATGGTCACACTGCTGCTTACCTGCTGTATTCTTCCTCTCTCCG** 691

**Nile tilapia LWS (S180)**  592 **ACTTCCTGTGGGCCTGATGTGTTCAGTGGAAGTGAAGACCCTGGAGTACAGTCCTACATGATTGTTCTCATGATTACTTGCTGTATCATCCCCCTGGGTA** 691

**Fugu LWS (A180)**  592 **ACCTCCTGTGGACCTGATGTGTTCAGCGGCAGCGAGGACCCAGGAGTCCAGTCCTACATGATCGTTCTCATGATCACATGCTGTATAATTCCTCTGGCTA** 691

**Pufferfish LWS (P180)**  592 **ACCTCCTGCGGACCTGATGTGTTCAGCGGCAGCGAGGACCCAGGCGTCCAGTCCTACATGATCGTTCTCATGATTACCTGCTGTATAATCCCTCTGGCCA** 691

**Turbot LWS (P180)_**  592 **ACCTCCTGTGGACCTGATGTATTTAGCGGAAGTGAAGACCCTGGAGTCCAGTCCTACATGATTGTTCTTATGGTTACATGTTGTTTCCTTCCCCTGGCTA** 691

**Winter flounder LWS (S180)**  592 **ACCTCCTGTGGACCTGATGTATTTAGTGGAAGTGAAGACCCTGGAGTCCAGTCCTACATGATTGTTCTTATGCTTACATGTTGTATTCTTCCCCTGGGTG** 691

**Goldfish LWS (S180)**  592 **ACCTCCTGTGGGCCTGATGTCTTCAGCGGAAGTGAGGACCCCGGTGTCCAGTCCTACATGATTGTCCTGATGATCACCTGCTGTATCATCCCTCTGGCCA** 691

**Coho salmon LWS (A180)**  592 **ACTTCCTGCGGACCTGATGTGTTCGGAGGCAATGAGGATCCTGGAGTCAAGTCCTACATGATTACTCTCATGATTACATGCTGCTTCTTCCCCCTGTTCG** 691

**Atlantic halibut LWS (S180)**  592 **ACTTCCTGTGGACCTGATGTATTTAGTGGAAGTGGAGACCCCGGAGTCCAGTCCTACATGATTGTTCTTATGGTTACATGTTGTTTCCTTCCCCTGTCTG** 691

**Carp LWS (S180)**  592 **ACCTCCTGTGGGCCTGATGTCTTCAGTGGAAGCGAGGACCCTGGTGTCCAGTCCTACATGATTGTCCTGATGATCACCTGCTGTATCATCCCTCTGGCTA** 691

**Sea Chub LWS (S180)**  333 **ACTTCCTGTGGACCTGATGTATTCAGTGGAAGTGAAGACCCCGGAGTTCAGTCCTACATGATTGTTCTTATGATCACATGTTGTATCATTCCTCTGGCTA** 432

**Arctic lamprey LWS (P180)**  598 **ACGTCGTGCGGGCCGGACGTGTTCAGCGGCAGCTCGGACCCCGGCGTGCAGTCCTACATGGTGGTGCTCATGGTCACCTGCTGCTTCCTCCCCCTCTCCG** 697

**710 720 730 740 750 760 770 780 790 800**

**....|....|....|....|....|....|....|....|....|....|....|....|....|....|....|....|....|....|....|....|**

**Guppy LWS S180**  688 **TCATCATCTTGTGCTACCTGGCTGTGTGGTTGGCCATCCATGCTGTTGCTATGCAGCAGAAGGAATCTGAGTCGACCCAGAAGGCTGAGAGAGAAGTGTC** 787

**Guppy LWS A180**  689 **TCATCATCTTGTGCTACCTGGCTGTGTGGTTGGCCATCCGTGCTGTTGCTATGCAGCAGAAGGAATCTGAGTCGACCCAGAAGGCTGAGAGAGAAGTGTC** 788

**Guppy LWS P180**  589 **TCATCATCTTGTGCTACCTGGCTGTATGGTTGGCCATCCGTGCTGTTGCTATGCAGCAGCTAGATAGTGAATCAACCCAGAAGGCTGAAAGAGAAGTGTC** 688

**Guppy LWS S180r**  590 **TCATCATCTTGTGCTACCTGGCAGTGTGGTTGGCCATCCGTGACATTGCTATGCAGCAGAAGGAATGCGAGTCGACCCAGAACGCCCAGAAGGAAGTATC** 689

**P. bifurca LWS S180**  688 **TCATCATCTTGTGCTACCTGGCTGTGTGGTTGGCCATCCGTGCTGTTGCTATGCAGCAGAAGGAATCCGAGTCGACCCAGAAGGCTGAGAGAGAAGTGTC** 787

**P. bifurca LWS A180**  507 **TCATCATCTTGTGCTACCTGGCTGTGTGGTTGGCCATCCGTGCTGT-GCTATGCAGCAGAAGGAATCCGAGTCGACCCAGAAGGCTGAGAGAGAAGTGTC** 605

**P. bifurca LWS P180**  689 **TCATCATCTTGTGCTACCTGGCTGTGTGGTTGGCCATCCGTGCTGTTGCTATGCAGCAGCTAGATAGTGAATCAACTCAGAAGGCTGAGAGAGAAGTGTC** 788

**P. bifurca LWS S180r**  590 **TCATCGTCTTGTGCTACCTGGCAGTGTGGTTGGCCATCCGCGACATTGCTATGCAGCAGAAGGAATGCGAGTCGACCCAGAACGCCCAGAAGGAAGTATC** 689

**P. parae LWS S180**  689 **TCATCATCTTGTGCTACCTGGCTGTGTGGTTGGCCATCCGTGCTGTTGCTATGCAGCAGAAGGAATCCGAGTCGACCCAGAAGGCTGAGAGGGAAGTGTC** 788

**P. parae LWS P180**  688 **TCATCATCTTGTGCTACCTGGCTGTGTGGTTGGCCATCCGTGCTGTTGCTATGCAGCAGCTAGATAGTGAATCAACCCAGAAGGCTGAGAGAGAAGTGTC** 787

**P. Parae LWS S180r**  590 **TCATCGTCTTGTGCTACCTGGCAGTGTGGTTGGCCATCCGTGACATTGCTATGCAGCAGAAGGAATGCGAGTCAACCCAGAATGCCCAGAAGGAAGTATC** 689

**P. picta LWS S180**  589 **TCATCATCTTGTGCTACCTGGCTGTGTGGTTGGCCATCCGTGCTGTTGCTATGCAGCAGAAGGAATCCGAGTCGACCCAGAAGGCTGAGAGAGAAGTGTC** 688

**P. picta LWS A180**  503 **TCATCATCTTGTGCTACCTGGCTGTGTGGTTGGCCATCCGTGCTGT-GCTATGCAGCAGAAGGAATCTGAGTCGACCCAGAAGGCTGAGAGAGAAGTGTC** 601

**P. picta LWS P180**  589 **TCATCATCTTGTGCTACCTGGCTGTGTGGTTGGCCATCCGTGCTGTTGCTATGCAGCAGCTAGATAGTGAATCAACCCAGAAGGCTGAGAGAGAAGTGTC** 688

**P. Picta LWS S180r**  590 **TCATCTTCTTGTGCTACCTGGCAGTGTGGTTGGCTATCCGTGACATTGCTATGCAGCAGAAGGAATGCGAGTCGACCCAGAATGCCCAGAAGGAAGTATC** 689

**Xiphophorus LWS S180**  508 **TCATCATCTTGTGCTATCTGGCTGTGTGGTTGGCCATCCGTGCTGTTGCTATGCAGCAGAAGGAATCTGAGTCGACCCAGAAGGCTGAGAGAGAAGTGTC** 607

**Xiphophorus LWS P180**  508 **TCATCATCTTGTGCTACCTGGCTGTGTGGTTGGCCATCCATGCTGTTGCTATGCAGCAGCTAGATAGTGAAACAACCCAGAAGGCTGAGAGAGAAGTGAC** 607

**Xiphophorus LWS S180r**  590 **TCATCATCTTGTGCTACCTGGCAGTGTGGTTGGCCATTCGTGACATTGCTATGCAGCAGAAGGAATGCGAGTCAACCCAGAATGCCCAGAAGGAAGTATC** 689

**Tomeurus LWS S180**  508 **TCATCGTCTTGTGCTACCTGGCTGTATGGTTGGCCATCCGCGCTGTTGCTATGCAGCAGAAGGAATCCGAGTCGACCCAGAAGGCTGAGAGGGAAGTGTC** 607

**Zebrafish LWS 1 (A180)**  692 **TTATCATTCTCTGCTACATTGCTGTGTACCTGGCCATCCATGCTGTTGCCCAGCAGCAGAAGGATTCTGAGTCCACACAGAAGGCCGAGAAGGAAGTGTC** 791

**Zebrafish LWS-2 (A180)**  689 **TCATCATTCTCTGCTACATTGCTGTGTTCCTGGCCATCCATGCTGTTGCCCAGCAGCAGAAGGATTCTGAGTCCACACAGAAGGCCGAGAAGGAAGTGTC** 788

**Jap. rice fish M/LWSA (S180)**  692 **TCATCATCCTGTGTTATCTTGCTGTCTGGCTGGCTATCCGTGCTGTTGCCATGCAGCAGAAGGAATCAGAGTCAACCCAGAAGGCTGAAAAAGAAGTGTC** 791

**Jap. rice fish M/LWSB (S180)**  692 **TCATCATCCTGTGTTACCTTGCTGTCTGGCTGGCTATCCGTGCTGTTGCCATGCAGCAGAAGGAATCAGAGTCAACCCAGAAGGCTGAAAGAGAAGTGTC** 791

**Bluefin killifish LWSA (S180)**  692 **TAATCATCCTGTGTTACCTGGCTGTATGGTTGGCCATCCGAGCTGTTGCTATGCAACAGAAGGAATCCGAGTCAACCCAGAAGGCTGAGAGGGAAGGGTC** 791

**Bluefin killifish LWSB (S180)**  599 **TCATCATCCTGTGCTACCTGGCGGTGTGGTTGGCCATCCGTGATATTGCTCAGCAGCAGAAGGAATGTGAGACAACTCAGAATGCTCAGAAGGAAGTTTC** 698

**Human LWS (S180)**  701 **TCATCATGCTCTGCTACCTCCAAGTGTGGCTGGCCATCCGAGCGGTGGCAAAGCAGCAGAAAGAGTCTGAATCCACCCAGAAGGCAGAGAAGGAAGTGAC** 800

**Human MWS (A180)**  701 **TCATCGTGCTCTGCTACCTCCAAGTGTGGCTGGCCATCCGAGCGGTGGCAAAGCAGCAGAAAGAGTCTGAATCCACCCAGAAGGCAGAGAAGGAAGTGAC** 800

**Ayu smelt LWS AYU-R (S180)**  692 **TCATCATCCTTTGCTACATTGCAGTGTGGCTGGCCATCCGTGCTGTGGCCCAGCAGCAGAAAGACTCTGAGTCTACACAGAAAGCTGAGAAGGAAGTGTC** 791

**Ayu smelt LWS Red-sens. (S180)** 692 **TCATCATCCTTTGCTACATTGCAGTGTGGCTGGCCATCCGTGCTGTGGCCCAGCAGCAGAAAGACTCTGAGTCTACACAGAAAGCTGAGAAGGAAGTGTC** 791

**Blind cave fish R007 (S180)**  692 **TTATCATTCTCTGCTACATCGCTGTGTGGTGGGCCATTCGCACTGTCGCTCAGCAGCAGAAAGACTCAGAATCAACACAGAAAGCAGAGAAGGAAGTGTC** 791

**Blind cave fish G101 (A180)**  686 **TCATTATCATTTGCTACATTTTTGTCTGGAGTGCCATCCACCAGGTCGCCCAGCAGCAGAAAGACTCAGAGTCCACTCAGAAGGCAGAGAAGGAAGTGTC** 785

**Blind cave fish G103 (A180)**  692 **TCATTATCATTTGCTACATCTTCGTCTGGAATGCCATCCACCAGGTCGCCCAGCAGCAGAAAGACTCAGAGTCTACCCAGAAGGCAGAGAAGGAAGTGTC** 791

**Nile tilapia LWS (S180)**  692 **TCATCATCCTGTGCTACCTTGCTGTGTGGCTGGCCATCCGTGCTGTTGCCATGCAGCAAAAGGAGTCAGAGTCAACCCAGAAGGCTGAGAAGGAAGTCTC** 791

**Fugu LWS (A180)**  692 **TCATCATCTTGTGCTACCTTGCAGTGTGGCTGGCTATCAGATCAGTTGCCATGCAACAGAAGGAATCAGAATCCACCCAGAAAGCTGAGAAAGAAGTGTC** 791

**Pufferfish LWS (P180)**  692 **TCATCGTCCTGTGCTACCTTGCAGTGTGGATGGCCATCAGAGCAGTCGCCATGCAGCAGAAGGAGTCAGAATCCACCCAGAAAGCTGAGAGGGAAGTGTC** 791

**Turbot LWS (P180)_**  692 **TTATCATCTTGTGCTACCTTGCCGTCTGGTGGGCTATCCATTCAGTTGCATTGCAGCAGAAGGAATCAGAGTCAACCCAGAAAGCTGAGAAAGATGTATC** 791

**Winter flounder LWS (S180)**  692 **TTATCATCTTGTGCTACTTGGCCGTCTGGTGGGCCATCCATTCTGTTGCGATGCAGCAGAAGGAATCGGAGTCAACCCAGAAAGCTGAGAGAGAAGTGTC** 791

**Goldfish LWS (S180)**  692 **TTATCATTCTCTGCTACATTGCTGTGTGGCTTGCCATTCGTACTGTCGCCCAGCAGCAGAAGGATTCTGAGTCCACACAGAAAGCAGAGAAGGAAGTGTC** 791

**Coho salmon LWS (A180)**  692 **TGATCATCTTCTGCTACATTTTCGTGTGGCTAGCCATTCGTGCTGTTGCTCAACAGCAAAAAGACTCTGAGTCAACACGGAAGGCCGAGAAAGAAGTGTC** 791

**Atlantic halibut LWS (S180)**  692 **TTATCATCTTGTGCTACCTTGCCGTCTGGTGGGCCATCCATTCTGTTGCGCTGCAGCAGAAGGAATCGGAGTCAACCCAGAAAGCTGAGAAAGAAGTGTC** 791

**Carp LWS (S180)**  692 **TTATCATTCTCTGCTACATTGCTGTGTGGCTTGCCATTCGTGCTGTCGCCCAGCAGCAGAAGGATTCTGAGTCCACACAGAAAGCAGAGAAGGAAGTGTC** 791

**Sea Chub LWS (S180)**  433 **TCATCATCTTGTGCTACCTTGCTGTCTGGTTGGCTATCCGTGCCGTTGCCATGCAGCAGAAGGAATCAGAGTCAACCCAGAAAGCTGAGAGAGAAGTATC** 532

**Arctic lamprey LWS (P180)**  698 **TCATCATCCTCTGCTACTTACAAGTCTGGCTCGCCATACACTCTGTGGCGCAGCAGCAGAAGGAGTCTGAGACGACACAGAAGGCGGAGCGCGACGTCTC** 797

**810 820 830 840 850 860 870 880 890 900**

**....|....|....|....|....|....|....|....|....|....|....|....|....|....|....|....|....|....|....|....|**

**Guppy LWS S180**  788 **CAGGATGGTTGTAGTCATGATCATAGCTTACTGTGTCTGCTGGGGACCCTACACCTTCTTCGCCTGCTTTGCCGCAGCCAACCCCGGATACGCCTTCCAT** 887

**Guppy LWS A180**  789 **CAGGATGGTTGTAGTCATGATCATAGCTTACTGTGTCTGCTGGGGACCCTACACCTTCTTCGCCTGCTTTGCCGCAGCCAACCCTGGATACGCCTTCCAT** 888

**Guppy LWS P180**  689 **CAGGATGGTTATAGTCATGATCCTGGCTTTCTGTCTCTGTTGGGGACCATATGCCACTTTCGCCTGCTTTGCCGCAGCCAACCCCGGATACGCCTTCCAT** 788

**Guppy LWS S180r**  690 **TAGGATG---------------------------------------------------------------------------------------------** 696

**P. bifurca LWS S180**  788 **CAGGATGGTTGTAGTCATGATCGTAGCTTACTGTGTCTGCTGGGGACCCTACACCTTCTTCGCCTGCTTTGCCGCAGCCAACCCCGGATACGCCTTCCAT** 887

**P. bifurca LWS A180**  606 **CAGGATGGTTGTAGTCATG---------------------------------------------------------------------------------** 624

**P. bifurca LWS P180**  789 **CAGGATGGTTGTAGTCATGATCCTGGCTTTCTGTCTCTGCTGGGGACCATATGCCACTTTCGCCTGCTTTGCCGCAGCCAACCCCGGATATGCATTCCAT** 888

**P. bifurca LWS S180r**  690 **TAGGATG---------------------------------------------------------------------------------------------** 696

**P. parae LWS S180**  789 **CAGGATGGTTGTAGTCATGATCGTAGCTTACTGTGTCTGCTGGGGACCCTACACCTTCTTCGCCTGCTTTGCCGCAGCCAACCCCGGATACGCCTTCCAT** 888

**P. parae LWS P180**  788 **CAGGATGGTTGTAGTCATGATCCTGGCTTTCTGTCTCTGCTGGGGACCTTATGCCACTTTCGCCTGCTTTGCCGCAGCCAACCCCGGATACGCATTCCAT** 887

**P. Parae LWS S180r**  690 **TAGGATG---------------------------------------------------------------------------------------------** 696

**P. picta LWS S180**  689 **CAGGATGGTTGTAGTCATGATCGTAGCTTACTGTGTCTGCTGGGGACCCTACACCTTCTTCGCCTGCTTTGCCGCAGCCAACCCCGGATACGCCTTCCAT** 788

**P. picta LWS A180**  602 **CAGGATGGTTGTAGTCAT----------------------------------------------------------------------------------** 619

**P. picta LWS P180**  689 **CAGGATGGTTGTAGTCATGATCCTAGCTTTCTGTCTCTGCTGGGGACCATATGCCACTTTCGCCTGCTTTGCCGCAGCCAACCCCGGATACGCCTTCCAT** 788

**P. Picta LWS S180r**  690 **TAGGATG---------------------------------------------------------------------------------------------** 696

**Xiphophorus LWS S180**  608 **CAGGATGGTTGTAGTCATGATCATAGCTTACTGTGTCTGCTGGGGACCCTACACCTTTTTCGCCTGCTTTGCCGCAGCCAACCCCGGATACGCCTTCCAT** 707

**Xiphophorus LWS P180**  608 **CAGGATGGTTGTAGTCATGATCCTGGCTTTCTGTCTCTGCTGGGGACCATATGCTACTTTTGCCTGCTTTGCCGCGGCCAACCCCGGATACGCCTTCCAT** 707

**Xiphophorus LWS S180r**  690 **TAGGATG---------------------------------------------------------------------------------------------** 696

**Tomeurus LWS S180**  608 **CAGGATGGTTGTGGTCATGATCCTAGCCTACTGCGTCTGCTGGGGACCTTACACCTTCTTCGCCTGCTTTGCCGCCGCCAACCCCGGATACGCCTTCCAT** 707

**Zebrafish LWS 1 (A180)**  792 **CAGAATGGTGGTTGTCATGATCTTCGCCTACTGTTTCTGCTGGGGTCCTTACACGTTCTTCGCCTGCTTTGCAGCTGCAAACCCAGGCTATGCCTTCCAC** 891

**Zebrafish LWS-2 (A180)**  789 **CAGAATGGTGGTTGTCATGATCCTTGCTTTCTGCCTTTGTTGGGGTCCGTACACGGCCTTTGCCTGCTTTGCAGCTGCAAACCCAGGCTATGCCTTCCAC** 888

**Jap. rice fish M/LWSA (S180)**  792 **CAGGATGGTGGTGGTCATGATCGTTGCTTACTGTGTGTGCTGGGGACCCTACACCTTTTTCGCCTGCTTTGCTGCAGCCAACCCCGGATATGCCTTCCAT** 891

**Jap. rice fish M/LWSB (S180)**  792 **CAGGATGGTGGTGGTCATGATCGTTGCTTACTGTGTGTGCTGGGGACCCTACACCTTTTTCGCCTGCTTTGCTGCAGCCAACCCCGGATATGCCTTCCAT** 891

**Bluefin killifish LWSA (S180)**  792 **CAGGATGGTTGTAGTCATGATCCTGGCTTACTGTGTCTGCTGGGGACCTTACACCTTTTTCGCCTGCTTCGCCGCAGCCAACCCCGGATATGCCTTCCAT** 891

**Bluefin killifish LWSB (S180)**  699 **CCGGATGGTCGTTGTCATGATCTTGGCTTACTGCGTCTGCTGGGGACCTTACACCTTTGTTGCCTGCTTTGCCGCGGSCAACCCCGGATATGCTTTCCAC** 798

**Human LWS (S180)**  801 **GCGCATGGTGGTGGTGATGATCTTTGCGTACTGCGTCTGCTGGGGACCCTACACCTTCTTCGCATGCTTTGCTGCTGCCAACCCTGGTTACGCCTTCCAC** 900

**Human MWS (A180)**  801 **GCGCATGGTGGTGGTGATGGTCCTGGCATTCTGCTTCTGCTGGGGACCATACGCCTTCTTCGCATGCTTTGCTGCTGCCAACCCTGGCTACCCCTTCCAC** 900

**Ayu smelt LWS AYU-R (S180)**  792 **CAGGATGGTTGTTGTGATGATCATTGCTTACATAGTTTGCTGGGGACCTTACACAGTCTTCGCCTGCTTTGCTGCTGCTAACCCAGGATATGCCTTCCAT** 891

**Ayu smelt LWS Red-sens. (S180)** 792 **CAGGATGGTTGTTGTGATGATCCTTGCTTACATAGTTTGCTGGGGACCTTACACAGTCTTCGCCTGCTTTGCTGCTGCTAACCCAGGATATGCCTTCCAT** 891

**Blind cave fish R007 (S180)**  792 **CAGGATGGTGGTGGTCATGATCATGGCTTATTGCTTCTGCTGGGGTCCTTACACTTTCTTTGCCTGCTTTGCGGCGGCTAACCCCGGCTACGCCTTCCAT** 891

**Blind cave fish G101 (A180)**  786 **CAGGATGGTGGTAGTGATGATCCTTGCCTTTATTGTGTGCTGGGGACCATATGCCTCCTTTGCCACCTTCTCTGCAGTGAACCCAGGTTATGCCTGGCAC** 885

**Blind cave fish G103 (A180)**  792 **CAGGATGGTGGTAGTGATGATCCTTGCCTTTATCCTGTGCTGGGGACCATATGCCTCCTTTGCCACCTTTTCTGCATTGAACCCTGGTTATGCCTGGCAC** 891

**Nile tilapia LWS (S180)**  792 **CAGGATGGTCGTTGTCATGATCGTGGCTTATTGTGTCTGCTGGGGACCTTACACCTTCTTTGCCTGCTTTGCTGCAGCCAACCCTGGATATGCCTTCCAC** 891

**Fugu LWS (A180)**  792 **CAGGATGGTGGTTGTCATGATTGTGGCATACTGTGTGTGTTGGGGACCGTACACCTTCTTTGCCTGTTTCGCTGCGGCTAACCCGGGATATGCCTTCCAT** 891

**Pufferfish LWS (P180)**  792 **CAGGATGGTGGTGGTCATGATTCTGGCCTACTGCGTCTGCTGGGGGCCGTACACCTTCTTTGCCTGTTTTGCTGCGGCTAACCCGGGATATGCCTTCCAT** 891

**Turbot LWS (P180)_**  792 **CAGAATGGTTGTTGTCATGATCTTGGCATATTGCGTATGCTGGGGACCTTACACCTTTTTTGCATGCTTTGCTGCGGCCAACCCAGGATATGCCTTCCAT** 891

**Winter flounder LWS (S180)**  792 **CAGAATGGTTGTTGTCATGATCGTGGCATATTGTGTCTGCTGGGGACCTTATACAGGCTTTGCCTGCTTCGCTGCAGCCAACCCTGGATATGCCTTCCAC** 891

**Goldfish LWS (S180)**  792 **CAGAATGGTGGTTGTCATGATCTTTGCCTACTGTTTTTGCTGGGGACCATACACATTCTGTGCATGCTTTGCTGCTGCAAACCCAGGCTATGCCTTCCAC** 891

**Coho salmon LWS (A180)**  792 **CAGGATGGTTGTTGTCATGATTATAGCTTACTGTGTATGCTGGGGACCTTATACCTGCTTTGCCTGCTTTGCTGCGGCTAACCCTGGGTATGCTTTCCAC** 891

**Atlantic halibut LWS (S180)**  792 **CAGAATGGTCGTTGTCATGATCGTGGCATATTGTGTCCGCTGGGGACCTTATACAGCCTTTGCCTGCTTTGCTGCGGCCAACCCTGGATATGCCTTCCAC** 891

**Carp LWS (S180)**  792 **CAGAATGGTGGTTGTCATGATTTTTGCATACTGTTTTTGCTGGGGACCTTACACGTTCTTCGCATGCTTTGCTGCTGCAAACCCAGGCTATGCCTTCCAC** 891

**Sea Chub LWS (S180)**  533 **CAGGATGGTGGTTGTCATGATCATCGCATATTGTGTCTGCTGGGGACCTTATACCTTTTTTGCCTGCTTTGCTGCGGCTAACCCTGGATATGCCTTCCAT** 632

**Arctic lamprey LWS (P180)**  798 **GCGCATGGTGGTCGTCATGATCTTAGCCTACATCTTCTGCTGGGGACCCTACACATTCTTCGCGTGCTATGCGGCCGCGAACCCGGGCTACGCGTTCCAC** 897

**910 920 930 940 950 960 970 980 990 1000**

**....|....|....|....|....|....|....|....|....|....|....|....|....|....|....|....|....|....|....|....|**

**Guppy LWS S180**  888 **CCTTTGGCCGCTGCCATGCCTGCATACTTTGCCAAAAGCGCCACCATCTACAACCCTGTTATCTATGTCTTCATGAACCGACAGTTCCGCACATGCATCA** 987

**Guppy LWS A180**  889 **CCTTTGGCCGCTGCCATGCCTGCATACTTTGCCAAAAGCGCCACCATCTACAACCCTGTTATCTATGTCTTCATGAACCGACAG----------------** 972

**Guppy LWS P180**  789 **CCTGTAGCCGCTGCCATGCCTGCATACTTTGCCAAAAGCGCCACCATCTACAACCCTGTTATCTATGTCTTCATGAACCGACAGTTTCGCACATGCATCA** 888

**Guppy LWS S180r**  696 **----------------------------------------------------------------------------------------------------** 696

**P. bifurca LWS S180**  888 **CCTTTGGCCGCTGCCATGCCTGCATACTTTGCCAAAAGTGCCACCATCTACAACCCTATTATCTATGTCTTCATGAACCGACAGTTCCGCACATGCATCA** 987

**P. bifurca LWS A180**  624 **----------------------------------------------------------------------------------------------------** 624

**P. bifurca LWS P180**  889 **CCTTTGGCTGCTGCCATCCCTGCGTATTTAGCCAAAAGCGCTACCATCTATAACCCTGTTATCTATGTCTTCATGAATCGACAGTTTCGCACATGCATCA** 988

**P. bifurca LWS S180r**  696 **----------------------------------------------------------------------------------------------------** 696

**P. parae LWS S180**  889 **CCTTTGGCCGCTGCCATGCCTGCATACTTTGCCAAAAGTGCCACCATCTACAACCCTATTATCTATGTCTTCATGAACCGACAGTTCCGCACATGCATCA** 988

**P. parae LWS P180**  888 **CCTTTGGCTGCTGCCATTCCTGCGTATTTAGCCAAAAGCGCCACCATCTATAACCCTGTTATCTATGTCTTCATGAATCGACAGTTTCGCACATGCATCA** 987

**P. Parae LWS S180r**  696 **----------------------------------------------------------------------------------------------------** 696

**P. picta LWS S180**  789 **CCTTTGGCCGCTGCCATGCCTGCATACTTTGCCAAAAGTGCCACCATCTACAACCCTATTATCTATGTCTTCATGAACCGACAGTTCCGCACATGCATCA** 888

**P. picta LWS A180**  619 **----------------------------------------------------------------------------------------------------** 619

**P. picta LWS P180**  789 **CCTTTGGCTGCTGCCATCCCTGCGTATTTAGCCAAAAGCGCCACCATCTATAACCCTGTTATCTATGTCTTCATGAATCGACAGTTTCGCACATGCATCA** 888

**P. Picta LWS S180r**  696 **----------------------------------------------------------------------------------------------------** 696

**Xiphophorus LWS S180**  708 **CCTTTGGCCGCTGCCATGCCTGCATACTTTGCCAAAAGCGCCACCATCTACAACCCTGTTATCTATGTCTTCATGAACCGACAGTTCCGCACATGCATCA** 807

**Xiphophorus LWS P180**  708 **CCTTTGGCCGCTGCCATACCTGCATATTTAGCCAAAAGCGCCACCATCTACAACCCTGTTATCTATGTCTTCATGAACCGACAGTTCCGCACATGCATCA** 807

**Xiphophorus LWS S180r**  696 **----------------------------------------------------------------------------------------------------** 696

**Tomeurus LWS S180**  708 **CCGTTGGCCGCTGCCATGCCCGCGTACTTTGCCAAAAGCGCCACAATCTACAATCCGGTTATCTACGTCTTCATGAACCGACAGTTCCGCACATGCATCA** 807

**Zebrafish LWS 1 (A180)**  892 **CCACTGGCAGCAGCCATGCCTGCCTACTTTGCCAAGAGCGCCACCATCTACAACCCCGTCATTTATGTCTTCATGAACCGACAGTTCCGCGTATGCATCA** 991

**Zebrafish LWS-2 (A180)**  889 **CCACTGGCAGCAGCCATGCCTGCCTACTTTGCCAAGAGCGCCACCATCTACAACCCCATCATTTATGTCTTCATGAACCGACAGTTCCGCGTATGCATCA** 988

**Jap. rice fish M/LWSA (S180)**  892 **CCTCTGGCTGCTGCCATGCCTGCTTATTTTGCAAAGAGCGCCACAATCTACAACCCCATCATCTATGTCTTCATGAACAGACAGTTCCGCACATGCATCA** 991

**Jap. rice fish M/LWSB (S180)**  892 **CCTCTGGCTGCTGCCATGCCTGCTTATTTTGCAAAGAGCGCCACAATCTACAACCCCGTCATCTATGTCTTCATGAACAGACAGTTCCGCACATGCATCA** 991

**Bluefin killifish LWSA (S180)**  892 **CCTTTGGCTGCTGCCATGCCTGCATACTTTGCCAAGAGCGCCACCATTTACAACCCAGTTATCTACGTCTTTATGAACCGACAGTCCCGCACATGCATCA** 991

**Bluefin killifish LWSB (S180)**  799 **CCTTTGGGCGCTGCCATGCCTGCATACTTTGCCAAGAGCGCCACCATCTACAACCCAGTCATCTATGTCTTCATGAACCGACAGTTTCGCACATGCATCA** 898

**Human LWS (S180)**  901 **CCTTTGATGGCTGCCCTGCCGGCCTACTTTGCCAAAAGTGCCACTATCTACAACCCCGTTATCTATGTCTTTATGAACCGGCAGTTTCGAAACTGCATCT** 1000

**Human MWS (A180)**  901 **CCTTTGATGGCTGCCCTGCCGGCCTTCTTTGCCAAAAGTGCCACTATCTACAACCCCGTTATCTATGTCTTTATGAACCGGCAGTTTCGAAACTGCATCT** 1000

**Ayu smelt LWS AYU-R (S180)**  892 **CCTCTGGCTGCAGCACTACCAGCCTACTTTGCCAAGAGCGCCACTATCTACAATCCAGTCATCTATGTCTTCATGAACCGACAGTTCCGTGTTTGCATCA** 991

**Ayu smelt LWS Red-sens. (S180)** 892 **CCTCTGGCTGCAGCACTACCAGCCTACTTTGCCAAGAGCGCCACTATCTACAATCCAGTCATCTATGTCTTCATGAACCGACAGTTCCGTGTTTGCATCA** 991

**Blind cave fish R007 (S180)**  892 **CCACTAGCAGCAGCCATGCCTGCCTACTTCGCCAAGAGCGCCACCATCTACAACCCCGTCATCTACGTCTTCATGAACAGACAATTCCGCGTATGCATCA** 991

**Blind cave fish G101 (A180)**  886 **CCACTGGCAGCCGCTATGCCCGCTTACTTCGCCAAGAGTGCCACCATCTACAATCCCATCATTTACGTCTTCATGAACCGCCAGTTCCGGAGCTGTATCA** 985

**Blind cave fish G103 (A180)**  892 **CCACTGGCAGCTGCTCTGCCCGCTTACTTCGCCAAGAGTGCCACCATCTACAATCCCATCATTTATGTCTTCATGAACCGCCAGTTCCGGAGCTGTATCA** 991

**Nile tilapia LWS (S180)**  892 **CCTCTGGCTGCTGCTATGCCTGCATACTTTGCCAAGAGCGCTACTATCTACAACCCAATCATCTATGTCTTCATGAACAGACAGTTCCGCACATGCATCA** 991

**Fugu LWS (A180)**  892 **CCTCTGGCTGCTGCCATGCCTGCGTACTTTGCCAAGAGCGCCACCATTTACAATCCAGTTATCTACGTCTTCATGAACCGACAGTTCCGCGTTTGCATTA** 991

**Pufferfish LWS (P180)**  892 **CCTCTGGCTGCTGCCATGCCTGCTTACTTTGCCAAGAGCGCCACCATTTACAATCCAATTATCTACGTCTTCATGAACCGACAGTTCCGTGTTTGCATTA** 991

**Turbot LWS (P180)_**  892 **CCTCTGGCTGCTGCCATGCCCGCATACTTTGCCAAGAGCGCCACCATATACAACCCAATCATCTATGTCTTCATGAACCGACAGTTCCGCACATGCATCA** 991

**Winter flounder LWS (S180)**  892 **CCTCTGGCTGCTGCCATGCCTGCATACTTTGCTAAGAGTGCGACCATTTACAACCCTGTCATCTATGTATTCATGAACCGACAGTTCCGCCCATGCATCA** 991

**Goldfish LWS (S180)**  892 **CCACTGGCAGCAGCCATGCCTGCCTACTTTGCCAAGAGCGCCACCATCTACAACCCCATTATTTATGTCTTCATGAACCGACAGTTCCGTGTATGCATCA** 991

**Coho salmon LWS (A180)**  892 **CCTCTGGCAGCCGCAATTCCTGCCTACTTTGCCAAGAGCGCCACCATCTACAATCCAGTTATATATGTCTTCATGAACAAACAGTTCCGTACTTGCATCA** 991

**Atlantic halibut LWS (S180)**  892 **CCTCTGGCTGCTGCCATGCCTGCATACTTTGCCAAGAGCGCCACCATTTACAACCCTGTCATCTATGTATTCATGAACCGACAGTTCCGCACATGCATCA** 991

**Carp LWS (S180)**  892 **CCACTGGCAGCAGCCATGCCTGCCTACTTTGCCAAGAGCGCCACCATCTACAACCCCATCATTTATGTCTTCATGAACCGACAGTTCCGTGTATGCATCA** 991

**Sea Chub LWS (S180)**  633 **CCTCTGGCTGCTGCTATGCCTGCATACTTTGCCAAGAGCGCCACCATC----------------------------------------------------** 680

**Arctic lamprey LWS (P180)**  898 **CCGCTCACCGCCGCCCTCCCGGCGTACTTCGCCAAGAGCGCCACCATCTACAATCCCGTCATCTACGTGTTCATGAACAGACAGTTCCGCAACTGCATCA** 997

**1010 1020 1030 1040 1050 1060 1070 1080 1090 1100**

**....|....|....|....|....|....|....|....|....|....|....|....|....|....|....|....|....|....|....|....|**

**Guppy LWS S180**  988 **TGCAGCTCTTTGGCAAACAGGTGGATGATGGTTCTGAAGTGTCCACA---TCAAAGACAGAGGTCTCCTCT---------------GTGGCTCCTGCATA** 1069

**Guppy LWS A180**  972 **----------------------------------------------------------------------------------------------------** 972

**Guppy LWS P180**  889 **TGAGACTCTTTGGCAAACAGATGGATGATGATTCCGAAGTGTCCACA---TCGAAGACAGAGGTCTCGTCT---------------GTTGCACCTGAATA** 970

**Guppy LWS S180r**  696 **----------------------------------------------------------------------------------------------------** 696

**P. bifurca LWS S180**  988 **TGCAGCTCTTTGGCAAACAGGTGGATGATGGTTCTGAAGTGTCCACA---TCAAAGACAGAGGTCTCCTCT---------------GTGGCTCCTGCATA** 1069

**P. bifurca LWS A180**  624 **----------------------------------------------------------------------------------------------------** 624

**P. bifurca LWS P180**  989 **TGAAACTCTTTGGCAAACAGATGGATGATGATTCCGAAGTGTCCACA---TCGAAGACAGAGGTCTCATCT---------------GTTGCACCTGAATA** 1070

**P. bifurca LWS S180r**  696 **----------------------------------------------------------------------------------------------------** 696

**P. parae LWS S180**  989 **TGCAGCTCTTTGGCAAACAGGTGGATGATGGTTCTGAAGTGTCCACA---TCAAAGACAGAGGTCTCCTCT---------------GTGGCTCCTGCATA** 1070

**P. parae LWS P180**  988 **TGAAACTCTTTGGCAAACAGATGGATGATGATTCCGAAGTGTCCACA---TCAAAGACAGAGGTCTCATCT---------------GT-GCACCTGAATA** 1068

**P. Parae LWS S180r**  696 **----------------------------------------------------------------------------------------------------** 696

**P. picta LWS S180**  889 **TGCAGCTCTTTGGCAAACAGGTGGATGATGGTTCTGAAGTGTCCACA---TCAAAAACAGAGGTCTCCTCT---------------GTGGCTCCTGCATA** 970

**P. picta LWS A180**  619 **----------------------------------------------------------------------------------------------------** 619

**P. picta LWS P180**  889 **TGAAACTCTTTGGCAAACAGATGGATGATGATTCCGAAGTGTCCACA---TAGAAGACAGAGGTCTCGTCT---------------GTTGCACCTGAATA** 970

**P. Picta LWS S180r**  696 **----------------------------------------------------------------------------------------------------** 696

**Xiphophorus LWS S180**  808 **TGCAGCTCTTTGGCAAACAGGTGGATGATGGTTCTGAAGTGTCCACA---TCCAAGACAGAAGTCTCCTCT---------------GTGGCTCCTGCATA** 889

**Xiphophorus LWS P180**  808 **TGAAACTCCTTGGCAAAGAGACGGATGATAATTCCGAAGTGTCCACA---TCGAAGACAGAGGTCTCATCT---------------GTTGCACCTGAATA** 889

**Xiphophorus LWS S180r**  696 **----------------------------------------------------------------------------------------------------** 696

**Tomeurus LWS S180**  808 **TGCAGCTCTTTGGCAAACAGGTGGATGATGGTTCTGAAGTGTCCACA---TCAAAGACAGAGGTCTCCTCT---------------GTGGCTCCTGCATA** 889

**Zebrafish LWS 1 (A180)**  992 **TGCAGCTCTTTGGAAAGAAGGTGGATGATGGCTCTGAGGTGTCCACA---TCCAAAACAGAAGTGTCTTCT---------------GTGGCTCCTGCATA** 1073

**Zebrafish LWS-2 (A180)**  989 **TGCAGCTCTTTGGAAAGAAGGTGGATGATGGCTCTGAGGTGTCCACA---TCCAAAACAGAAGTGTCTTCT---------------GTGGCTCCTGCATA** 1070

**Jap. rice fish M/LWSA (S180)**  992 **TGCAGCTCTTTGGCAAGCAGGTGGATGATGGTTCTGAAGTTTCTACA---TCAAAGACAGAGGTCTCCTCT---------------GTGGCTCCTGCATA** 1073

**Jap. rice fish M/LWSB (S180)**  992 **TGCAGCTCTTTGGCAAACAGGTGGATGATGGTTCTGAAGTTTCTACA---TCAAAGACAGAGGTCTCCTCT---------------GTGGCTCCTGCATA** 1073

**Bluefin killifish LWSA (S180)**  992 **TGCAGCTCTTTGGCAAACAGGTGGACGATGGTTCTGAAGTGTCCACA---TCAAAGACAGAGGTCTCCTCT---------------GTGGCTCCTGCATA** 1073

**Bluefin killifish LWSB (S180)**  899 **TGCGGCTCTTTGGCAAAGAGACGGATGATGGTTCTGAAATCTCCACG---TCAAAGACAGAGGTCTCCTCT---------------GTGGCTCCTGAATA** 980

**Human LWS (S180)**  1001 **TGCAGCTTTTCGGGAAGAAGGTTGACGATGGCTCTGAACTCTCCAGCGCCTCCAAAACGGAGGTCTCATCTGTGTCCTCG------GTATCGCCTGCATG** 1094

**Human MWS (A180)**  1001 **TGCAGCTTTTCGGGAAGAAGGTTGACGATGGCTCTGAACTCTCCAGCGCCTCCAAAACGGAGGTCTCATCTGTGTCCTCG------GTATCGCCTGCATG** 1094

**Ayu smelt LWS AYU-R (S180)**  992 **TGCAGCTGTTTGGAAAGAAGGTTGATGATGGGTCTGAAGTATCCACA---TCCAAAACAGAAGTCTCATCT---------------GTGGCACCTGCATA** 1073

**Ayu smelt LWS Red-sens. (S180)** 992 **TGCAGCTGTTTGGCAAAAAGGTTGATGATGGGTCTGAAGTATCCACA---TCCAAGACTGAGGTGTCCTCT---------------GTTGCACCTGCATA** 1073

**Blind cave fish R007 (S180)**  992 **TGCAGCTCTTTGGGAAGAAGGTGGATGATGGATCTGAGGTGTCTACA---TCCAAGACAGAAGTCTCCTCT---------------GTGGCACCTGCATA** 1073

**Blind cave fish G101 (A180)**  986 **TGCAGCTGTTTGGAAAGAAGGTGGAGGATGCATCAGAGGTTTCCGGC---TCTACCACAGAAGTTTCTACA---------------------GCCTCGTA** 1061

**Blind cave fish G103 (A180)**  992 **TGCAGCTGTTTGGAAAGAAGGTGGAGGATGCATCAGAGGTTTCTGGC---TCTACCACAGAAGTGTCTACG---------------------GCTTCATA** 1067

**Nile tilapia LWS (S180)**  992 **TGCAGCTCTTTGGCAAACAAGTGGATGATGGCTCTGAAGTCTCCACA---TCAAAGACAGAGGTCTCCTCT---------------GTGGCTCCTGCATA** 1073

**Fugu LWS (A180)**  992 **TGAAGCTCTTTGGCAAAGAAGTGGATGATGGCTCTGAAGTATCTACA---TCGAAGACAGAGGTTTCCTCT---------------GTGGCTCCTGCATA** 1073

**Pufferfish LWS (P180)**  992 **TGAAGCTCTTTGGCAAAGAGGTGGACGATGGCTCCGAAGTATCCACA---TCAAAGACAGAGGTCTCCTCT---------------GTGGCCCCTGCATA** 1073

**Turbot LWS (P180)_**  992 **TGCAGCTCTTTGGCAAAGAAGTGGATGATGGCTCTGAAGTATCCACA---TCAAAGACAGAAGTCTCCTCT---------------GTGGCTCCGGCATA** 1073

**Winter flounder LWS (S180)**  992 **TGCAACTCTTTGGCAAAGAAGTGGAAGATGCTTCTGAGGTATCCTCA---TCAAAGACAGAGGTCTCATCT---------------GTGGCTCCTGCATA** 1073

**Goldfish LWS (S180)**  992 **TGCAGCTCTTTGGAAAGAAGGTAGATGATGGTTCCGAGGTGTCTACA---TCCAAGACAGAAGTGTCCTCT---------------GTGGCTCCTGCATA** 1073

**Coho salmon LWS (A180)**  992 **TGCAGCTCTTTGGAAAGGCAGAAGATGATGGCACTGAAGTGTCTACA---TCAAAAACAGAGGTTTCCTCT---------------GTGGCACCTGCATA** 1073

**Atlantic halibut LWS (S180)**  992 **TGCAACTCTTTGGCAAAGAAGTGGATGATGGTTCTGAAGTATCCACA---TCAAAGACAGAGGTCTCATCT---------------GTGGCTCCTGCATA** 1073

**Carp LWS (S180)**  992 **TGCAGCTCTTTGGAAAGAAGGTGGATGATGGCTCCGAGGTGTCCACA---TCCAAGACAGAAGTGTCCTCT---------------GTGGCTCCTGCATA** 1073

**Sea Chub LWS (S180)**  680 **----------------------------------------------------------------------------------------------------** 680

**Arctic lamprey LWS (P180)**  998 **TGCAGCTGTTTGGCAAGAAGGTGGACGACGGCTCCGAGGTGTCCAGCGCGTCCCGCACCGAAGTCTCGTCCGTCTCCAACTCCTCCATCAGCCCCGCGTG** 1097

**.**

**Guppy LWS S180**  1070 **A** 1070

**Guppy LWS A180**  972 **-** 972

**Guppy LWS P180**  971 **A** 971

**Guppy LWS S180r**  696 **-** 696

**P. bifurca LWS S180**  1070 **A** 1070

**P. bifurca LWS A180**  624 **-** 624

**P. bifurca LWS P180**  1071 **A** 1071

**P. bifurca LWS S180r**  696 **-** 696

**P. parae LWS S180**  1071 **A** 1071

**P. parae LWS P180**  1069 **A** 1069

**P. Parae LWS S180r**  696 **-** 696

**P. picta LWS S180**  971 **A** 971

**P. picta LWS A180**  619 **-** 619

**P. picta LWS P180**  971 **A** 971

**P. Picta LWS S180r**  696 **-** 696

**Xiphophorus LWS S180**  890 **G** 890

**Xiphophorus LWS P180**  890 **A** 890

**Xiphophorus LWS S180r**  696 **-** 696

**Tomeurus LWS S180**  890 **A** 890

**Zebrafish LWS 1 (A180)**  1074 **A** 1074

**Zebrafish LWS-2 (A180)**  1071 **A** 1071

**Jap. rice fish M/LWSA (S180)**  1074 **A** 1074

**Jap. rice fish M/LWSB (S180)**  1074 **A** 1074

**Bluefin killifish LWSA (S180)**  1074 **A** 1074

**Bluefin killifish LWSB (S180)**  981 **A** 981

**Human LWS (S180)**  1095 **A** 1095

**Human MWS (A180)**  1095 **A** 1095

**Ayu smelt LWS AYU-R (S180)**  1074 **A** 1074

**Ayu smelt LWS Red-sens. (S180)** 1074 **A** 1074

**Blind cave fish R007 (S180)**  1074 **A** 1074

**Blind cave fish G101 (A180)**  1062 **A** 1062

**Blind cave fish G103 (A180)**  1068 **A** 1068

**Nile tilapia LWS (S180)**  1074 **A** 1074

**Fugu LWS (A180)**  1074 **A** 1074

**Pufferfish LWS (P180)**  1074 **A** 1074

**Turbot LWS (P180)_**  1074 **A** 1074

**Winter flounder LWS (S180)**  1074 **A** 1074

**Goldfish LWS (S180)**  1074 **A** 1074

**Coho salmon LWS (A180)**  1074 **A** 1074

**Atlantic halibut LWS (S180)**  1074 **A** 1074

**Carp LWS (S180)**  1074 **A** 1074

**Sea Chub LWS (S180)**  680 **-** 680

**Arctic lamprey LWS (P180)**  1098 **A** 1098
